# Supplementary figures and images for: Uncovering population contributions to the extracellular potential in the mouse visual system using Laminar Population Analysis
Source: PLoS Comput Biol. 2024 Dec 12;20(12):e1011830. doi: 10.1371/journal.pcbi.1011830 (PMC11670981; doi:10.1371/journal.pcbi.1011830)

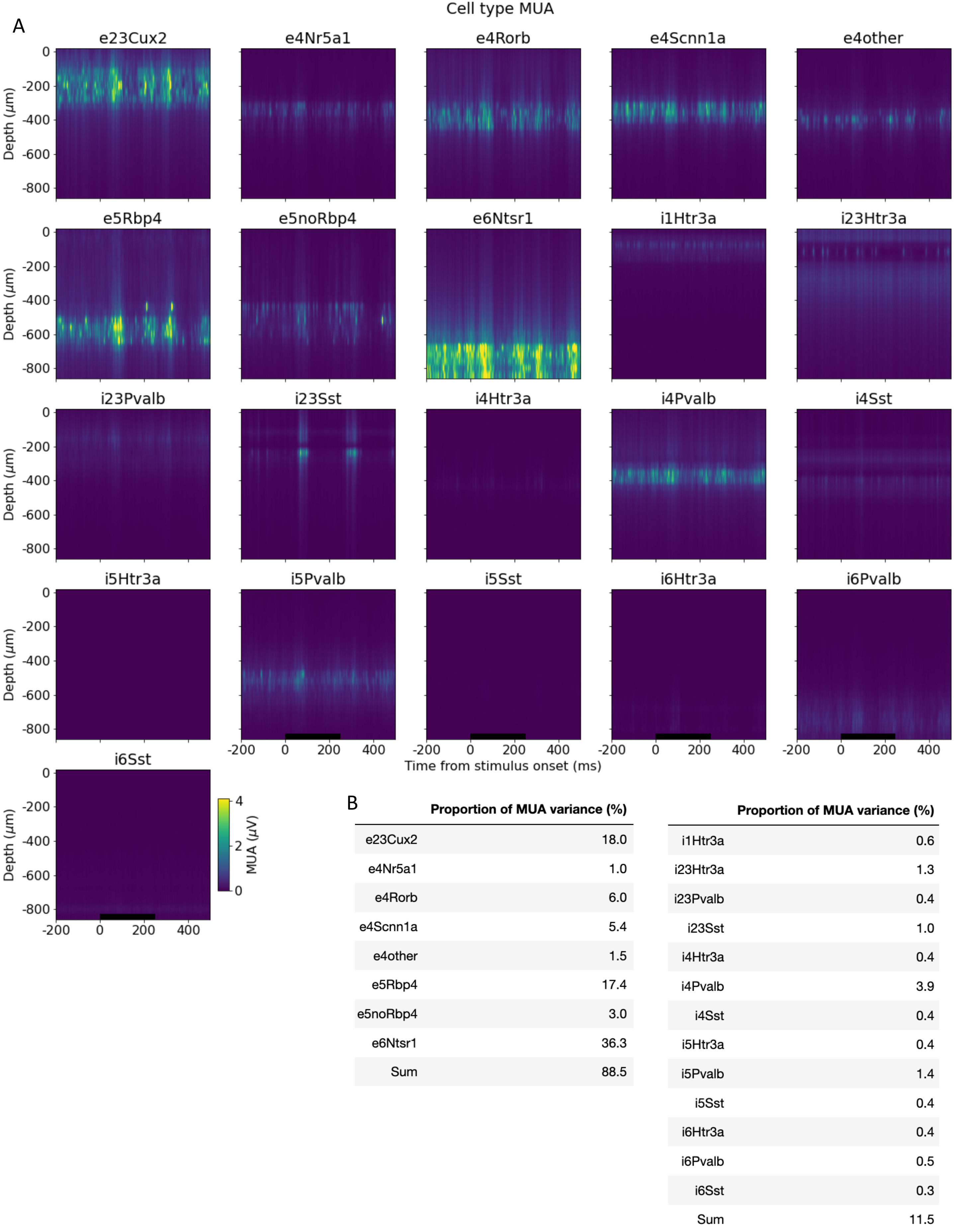

Supplement: S1 Fig — (A) MUA contribution of cell type families in the model. (B) Proportion of variance explained by excitatory (left) and inhibitory (right) cell type families. (TIF) [file pcbi.1011830.s001.tif]

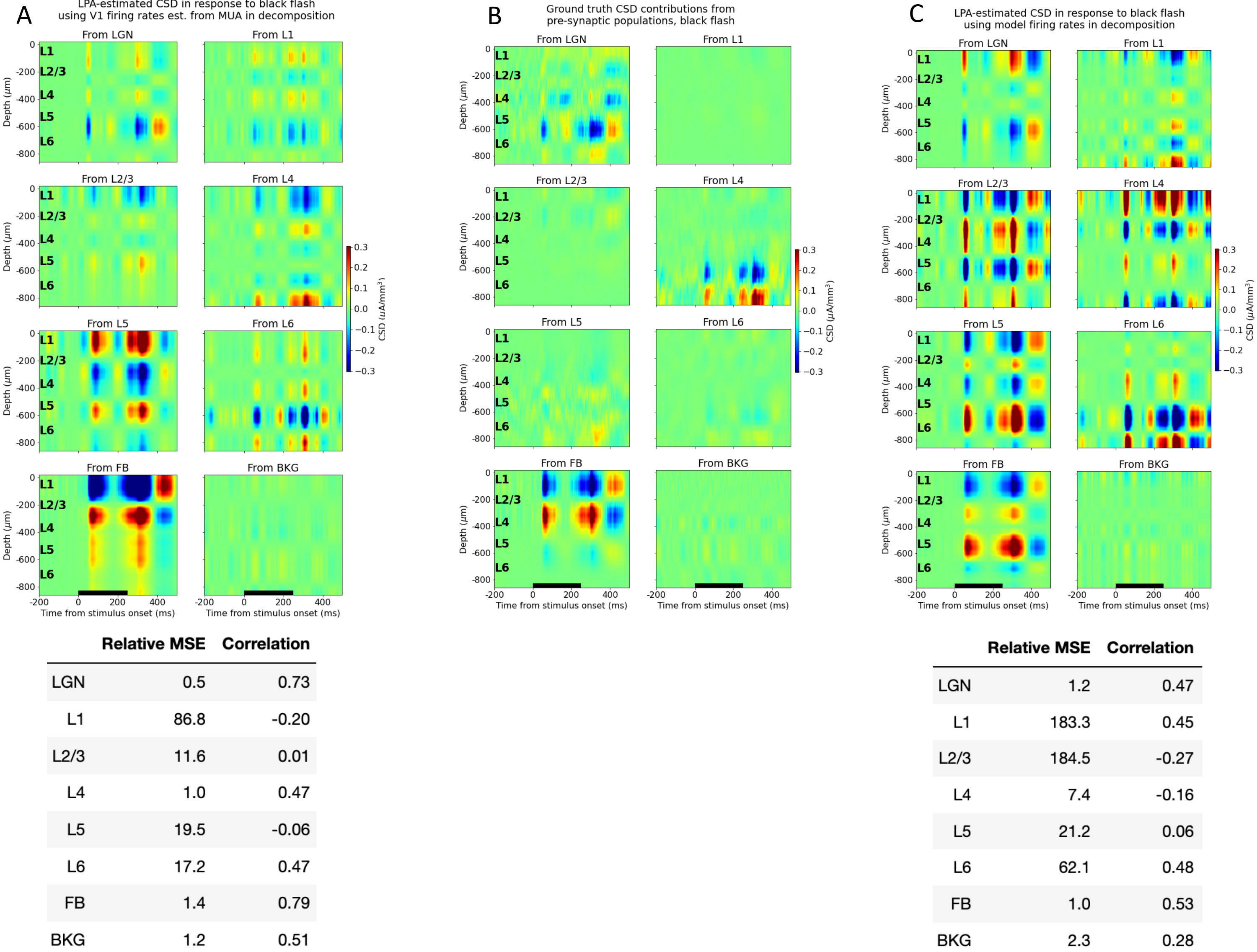

Supplement: S2 Fig — (A) Top: Black flash CSD generated from firing in each presynaptic population estimated with LPA using temporal profiles obtained from MUA as shown in Fig 2D. Bottom: Relative MSE and correlation between LPA-estimated and true black flash CSD contributions from each presynaptic population. (B) True Black flash CSD contributions from firing in each presynaptic population. (C) Top: Black flash CSD generated from firing in each presynaptic population estimated with LPA using temporal profiles obtained from firing rates of excitatory cells in the model in each layer from 2/3 to 6 and inhibitory cells in layer 1. Bottom: Relative MSE and correlation between LPA-estimated and true black flash CSD contributions from each presynaptic population. (TIF) [file pcbi.1011830.s002.tif]

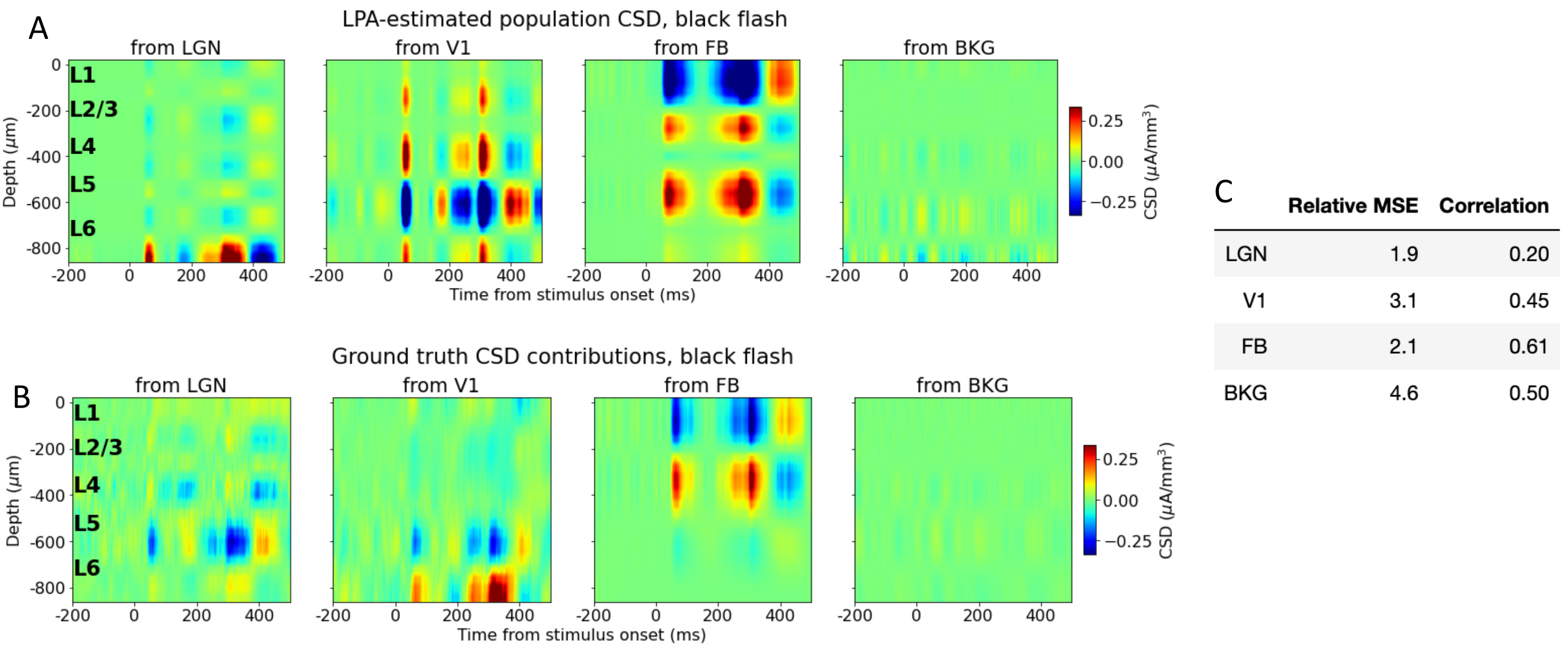

Supplement: S3 Fig — (A) Top: LPA-estimated black flash CSD generated from firing in each presynaptic population. Firing rates of excitatory cells in the model are used for the presynaptic temporal profiles of V1 in the decomposition. (B) True black flash CSD generated from firing in the same presynaptic populations. (C) Relative MSE and correlation between LPA-estimated and true black flash CSD generated from each presynaptic population. (TIF) [file pcbi.1011830.s003.tif]

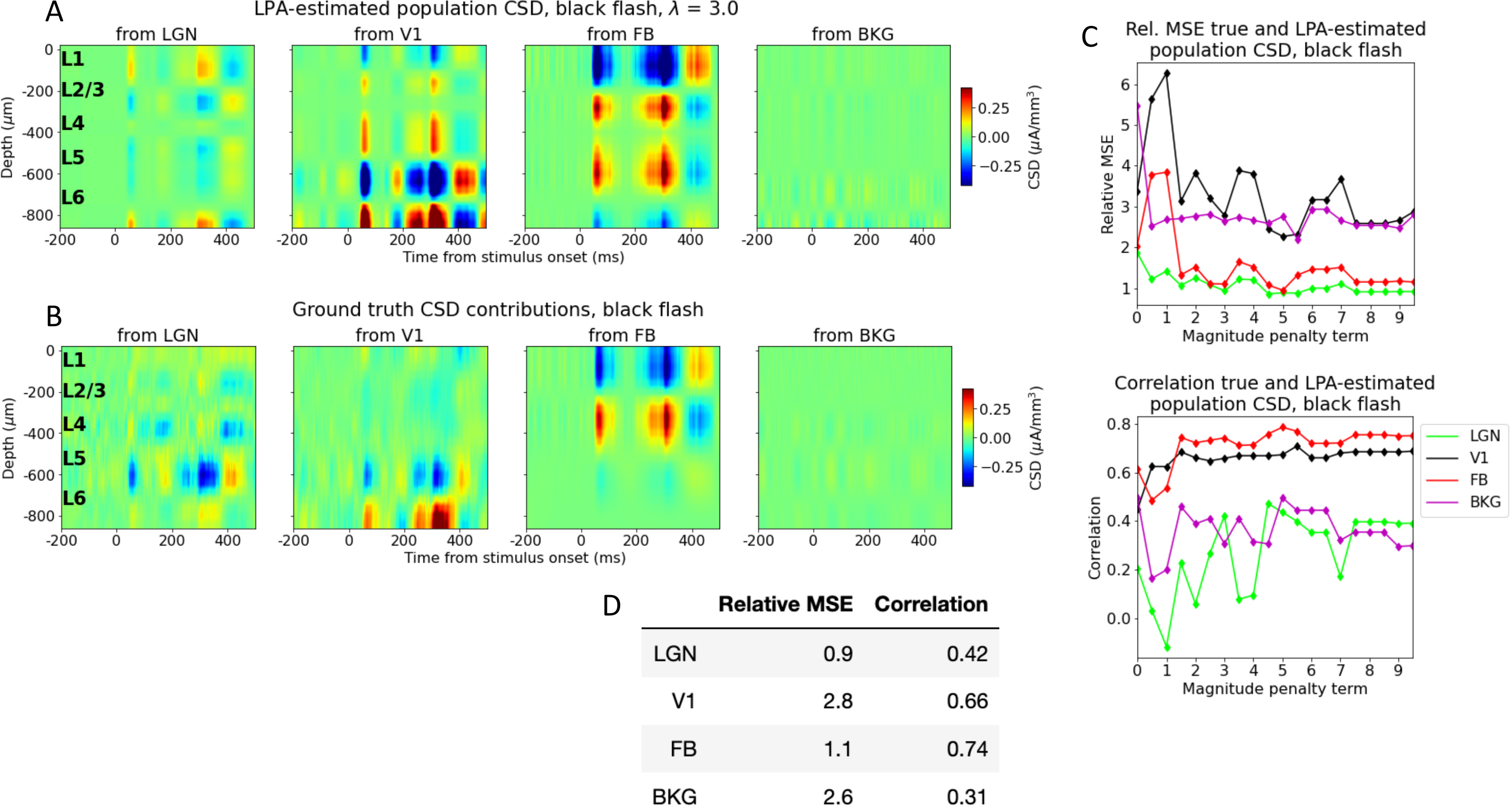

Supplement: S4 Fig — (A) LPA-estimated black flash CSD generated from firing in each presynaptic population for λ = 3, the value at which the deviation from 0 in the CSD summed across channels was smallest (Fig 5A). (B) True black flash CSD generated from firing in presynaptic populations. (C) Relative MSE (left) and correlation (right) between CSD generated from each presynaptic population estimated by CSD and true CSD generated from each presynaptic population at different values of λ. LGN: green, V1: black, feedback: red, background: purple. (D) Relative MSE and correlation between LPA-estimated and true black flash CSD generated from each presynaptic population at λ = 3. (TIF) [file pcbi.1011830.s004.tif]

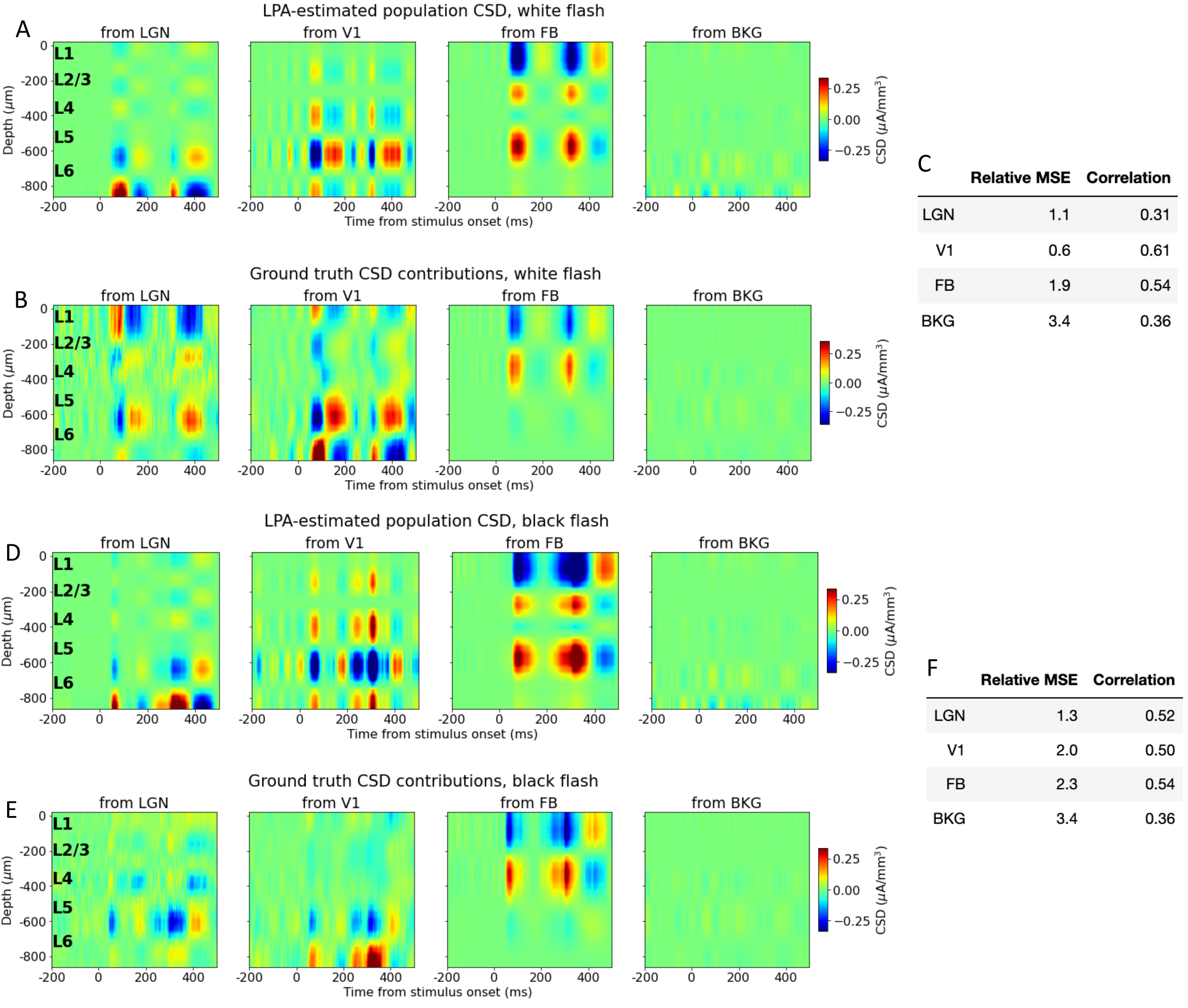

Supplement: S5 Fig — (A) Top: LPA-estimated white flash CSD generated from firing in each presynaptic population. (B) True white flash CSD generated from firing in the same presynaptic populations. (C) Relative MSE and correlation between LPA-estimated and true white flash CSD generated from each presynaptic population. (D) Top: LPA-estimated black flash CSD generated from firing in each presynaptic population. (E) True black flash CSD generated from firing in the same presynaptic populations. (F) Relative MSE and correlation between LPA-estimated and true black flash CSD generated from each presynaptic population. (TIF) [file pcbi.1011830.s005.tif]

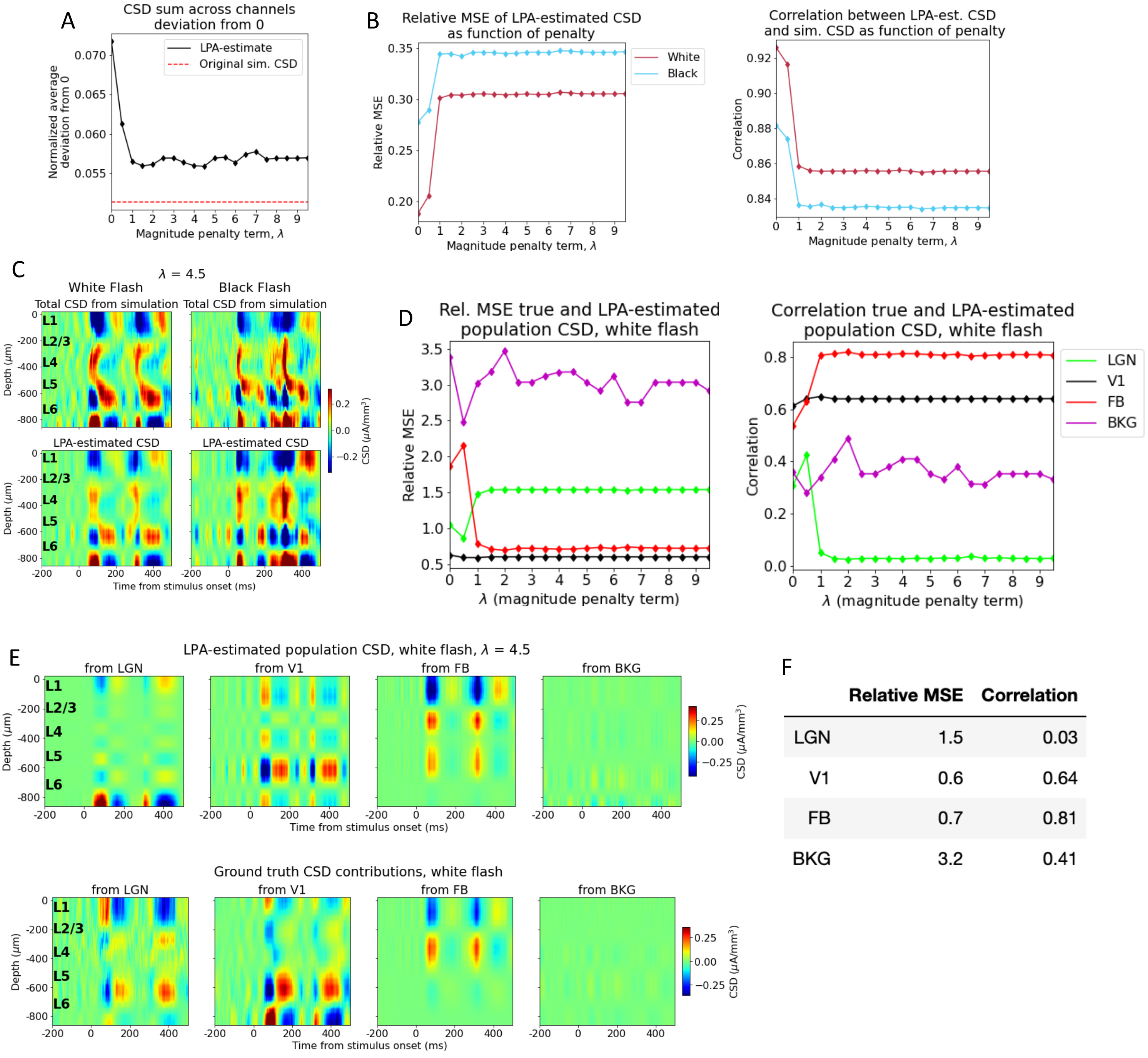

Supplement: S6 Fig — (A) Average sum of LPA-estimated total CSD across channels (black line) with varying penalty (λ) on deviations from 0. Red dashed line: Average deviation from 0 of sum across channels for total simulated CSD to which LPA was applied. (B) Relative MSE (left) and correlation (right) between CSD from simulation and CSD estimated from LPA-components at different penalty magnitudes with temporal profile of V1 firing rates estimated from MUA. White flash: dark red line, black flash: blue line. (C) Total simulated CSD (top) and CSD estimated from LPA-components (bottom) in response to white and black full-field flash when the penalty for deviations from zero λ = 4.5, which is the value at which the deviation from zero for the average sum across channels in the LPA-estimated CSD is smallest (see (A)). (D) Relative MSE (left) and correlation (right) between LPA-estimated and true white flash CSD generated from each presynaptic population at different values of λ. LGN: green, V1: black, feedback: red, background: purple. (E) Top: LPA-estimated white flash CSD generated from firing in each presynaptic population for λ = 4.5. Bottom: True white flash CSD generated from firing in presynaptic populations. (F) Relative MSE and correlation between LPA-estimated and true white flash CSD generated from each presynaptic population with λ = 4.5. (TIF) [file pcbi.1011830.s006.tif]

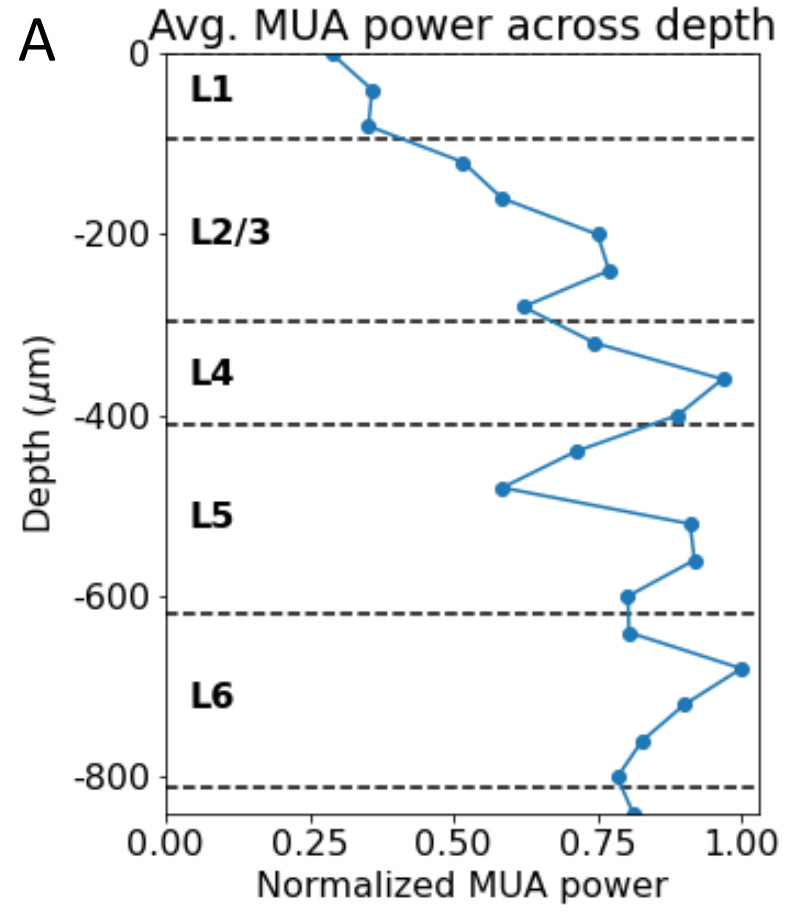

Supplement: S7 Fig — (A) The blue line shows the average MUA power at different channels of the simulated probe across all trials of white and black full-field flash stimuli. (TIF) [file pcbi.1011830.s007.tif]

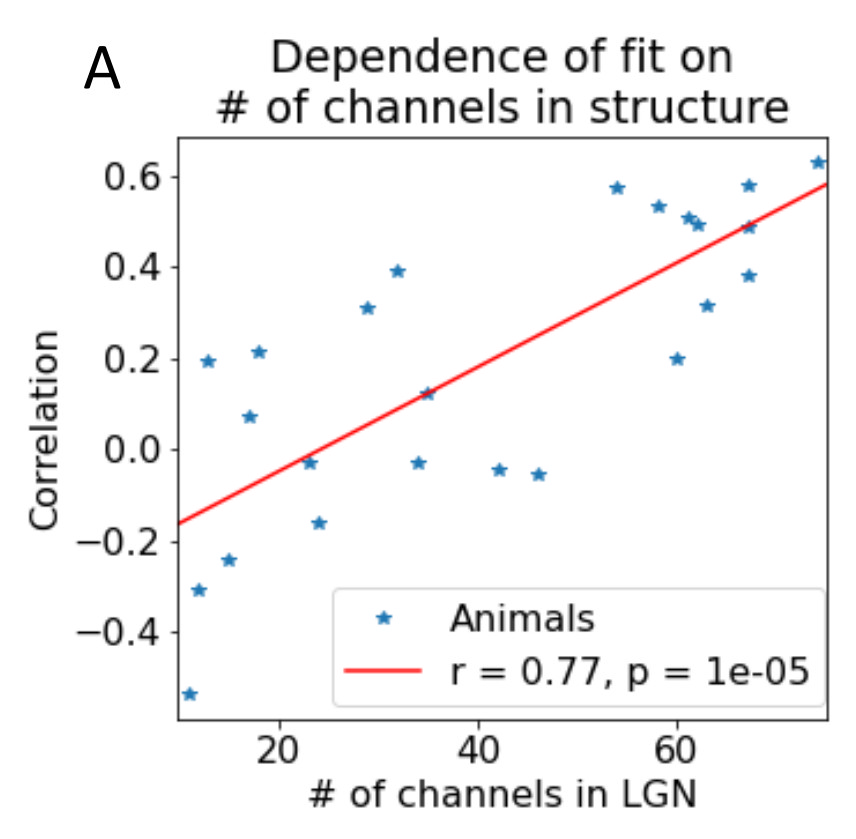

Supplement: S8 Fig — (A) Relationship between number of recording channels in LGN in experiments and the correlation between the LPA-estimated and simulated MUA in LGN. Each blue star corresponds to an animal in the dataset and the red line displays the results of a linear regression between number of recording channels in LGN and the correlation. (TIF) [file pcbi.1011830.s008.tif]

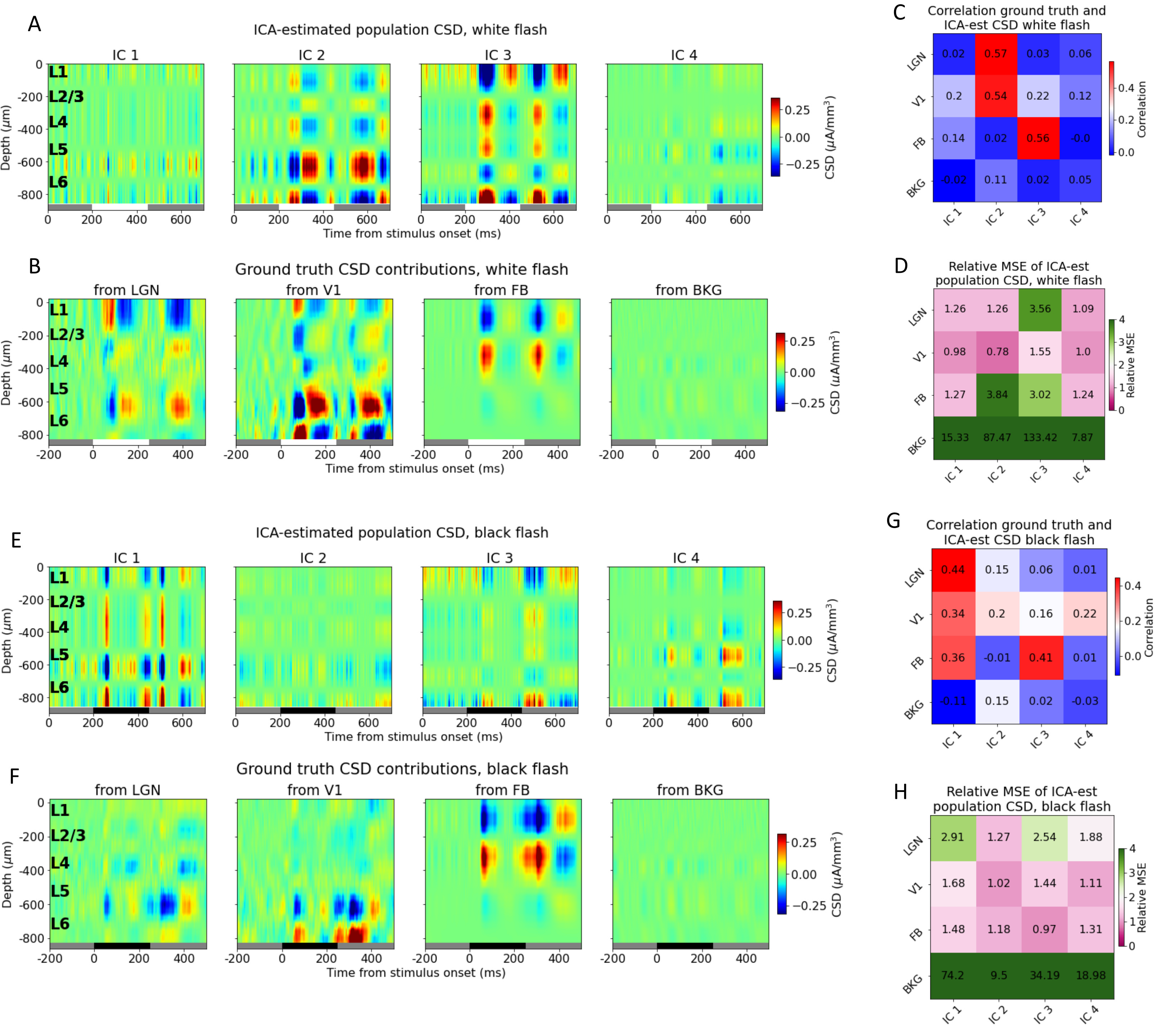

Supplement: S9 Fig — (A) Estimated white flash CSD of four ICA components. (B) True white flash CSD from each population. (C-D) Correlation and relative MSE, respectively, between CSD of ICA-components and true CSD in (A) and (B). (E) Estimated black flash CSD of four ICA components. (F) True black flash CSD from each population. (G-H) Correlation and relative MSE, respectively, between CSD of ICA-components and true CSD in (E) and (F). (TIF) [file pcbi.1011830.s009.tif]

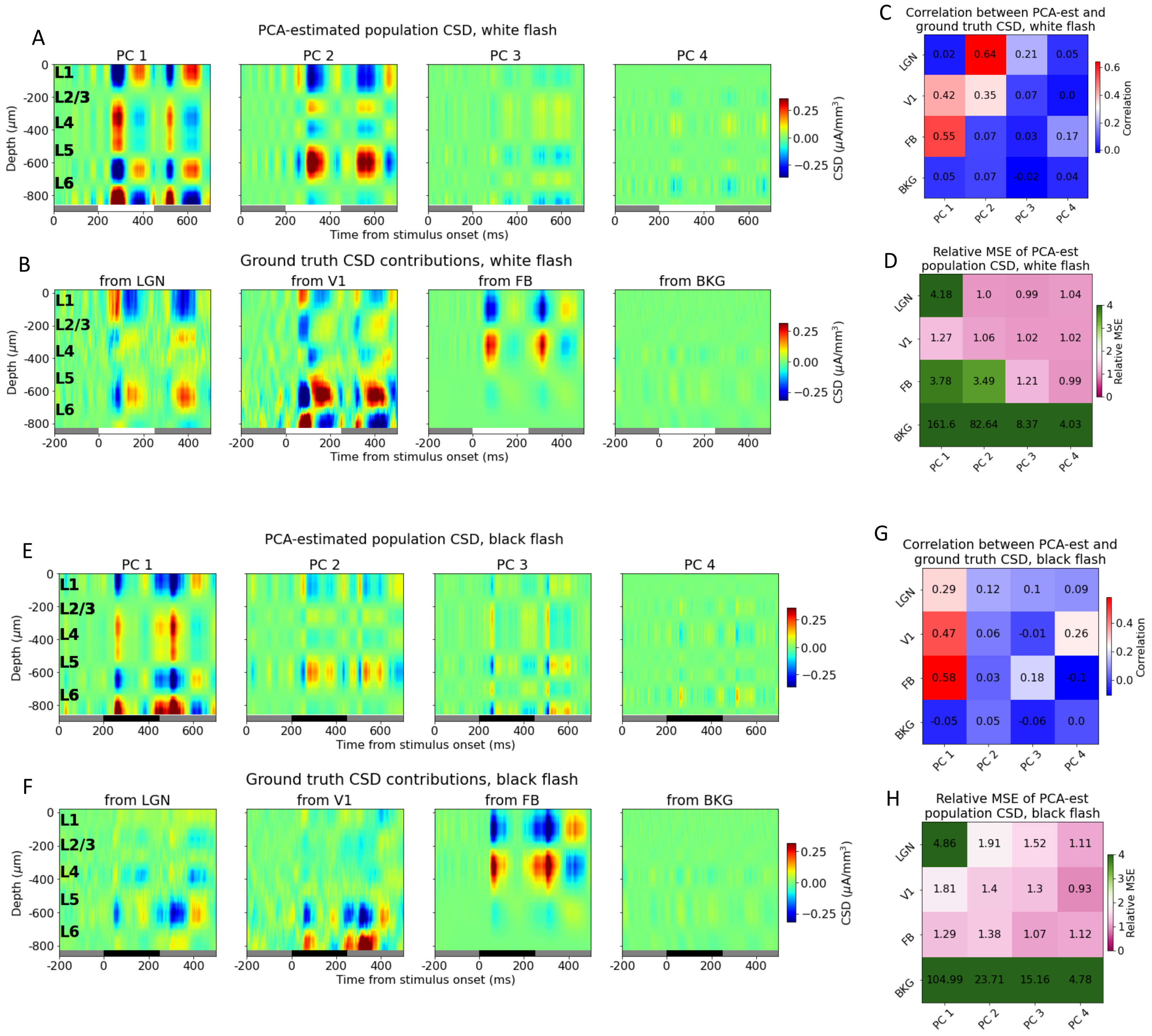

Supplement: S10 Fig — (A) Estimated white flash CSD of four PCA components. (B) True white flash CSD from each population. (C-D) Correlation and relative MSE, respectively, between CSD of PCA-components and true CSD in (A) and (B). (E) Estimated black flash CSD of four PCA components. (F) True black flash CSD from each population. (G-H) Correlation and relative MSE, respectively, between CSD of ICA-components and true CSD in (E) and (F). (TIF) [file pcbi.1011830.s010.tif]

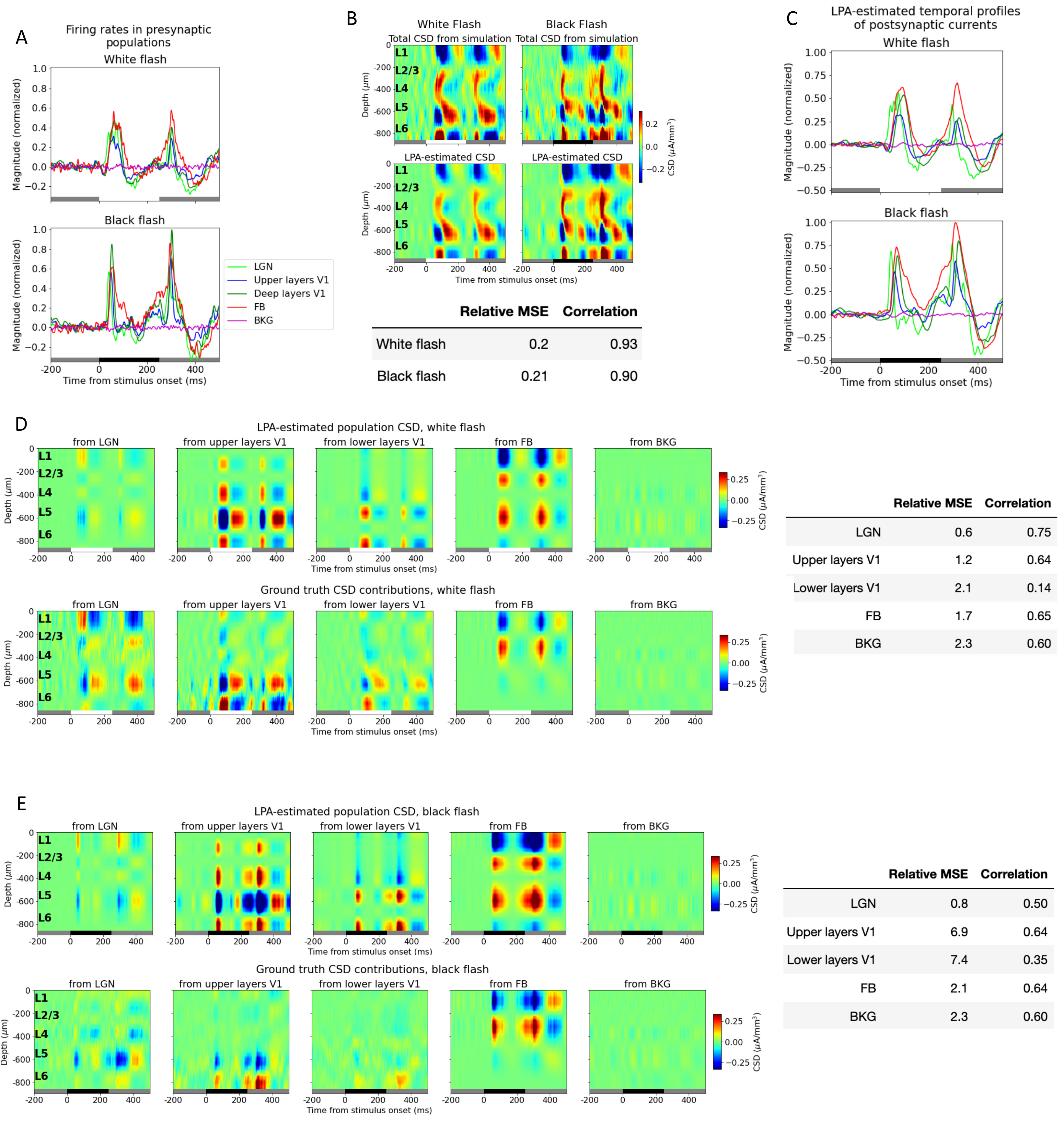

Supplement: S11 Fig — (A) Temporal profiles of firing rates of presynaptic excitatory populations in external structures (LGN: light green line; feedback: red line; background: purple line), upper layer V1 merged (blue line), and deep layers V1 merged (dark green line). (B) Top: Trial-averaged CSD from simulation with white (left) and (black) flash stimuli and CSD estimated from LPA-components using presynaptic firing rates in (A) in decomposition. Bottom: Relative MSE and correlation between CSD from simulation and CSD estimated from LPA-components. (C) Temporal profiles of CSD of each LPA-component in response to white and black flash. (D) Top: LPA-estimated white flash CSD generated from firing in each presynaptic population shown in (A). Bottom: True white flash CSD generated from firing in the same presynaptic populations. Right: Relative MSE and correlation between LPA-estimated and true white flash population CSD. (E) Top: LPA-estimated black flash CSD generated from firing in each presynaptic population shown in (A). Bottom: True black flash CSD generated from firing in the same presynaptic populations. Right: Relative MSE and correlation between LPA-estimated and true black flash population CSD. (TIF) [file pcbi.1011830.s011.tif]
