# Supplementary material for: Uncovering population contributions to the extracellular potential in the mouse visual system using Laminar Population Analysis
Source: PLoS Comput Biol. 2024 Dec 12;20(12):e1011830. doi: 10.1371/journal.pcbi.1011830 (PMC11670981; doi:10.1371/journal.pcbi.1011830)

Supplementary figure S12

Results from applying LPA to all 24 animals in Visual Coding data set

Applying LPA to MUA

Session ID: 71509703

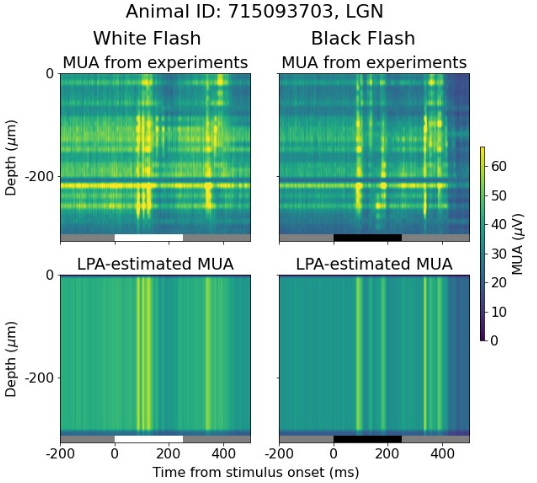

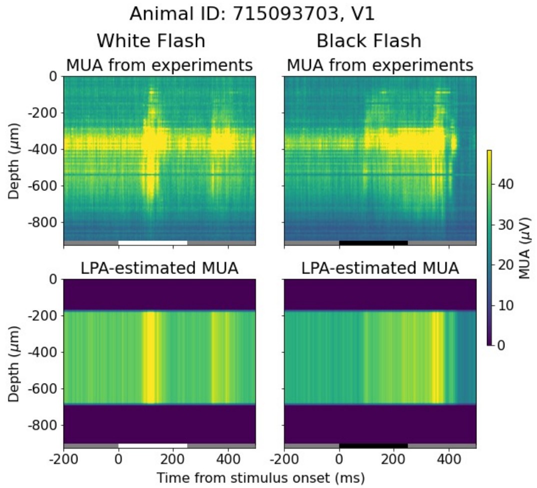

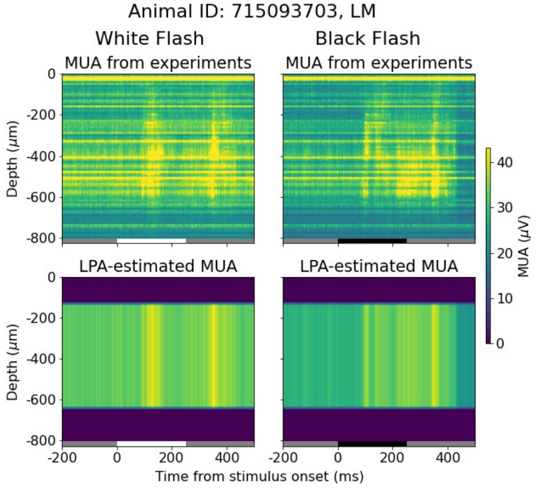

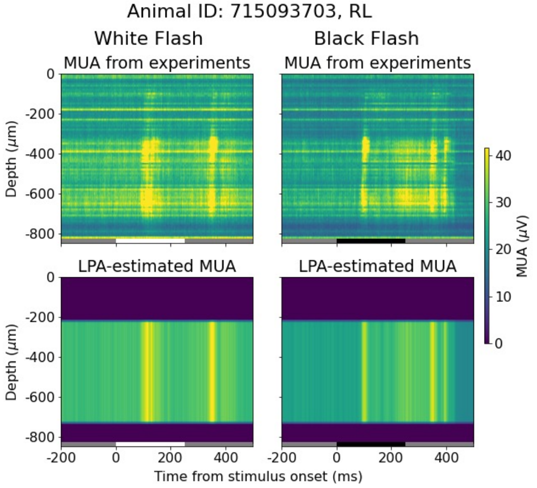

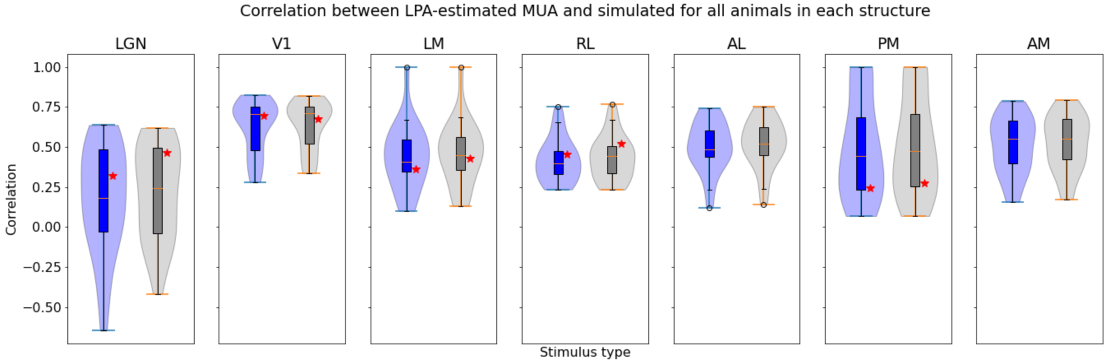

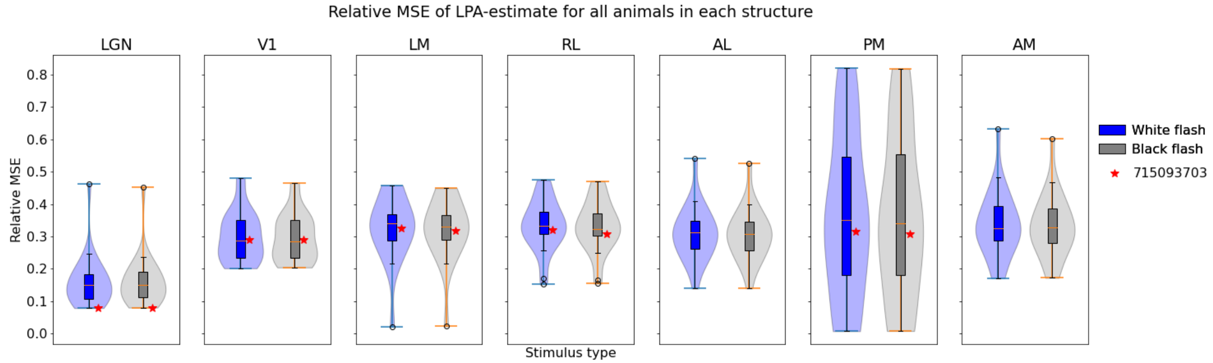

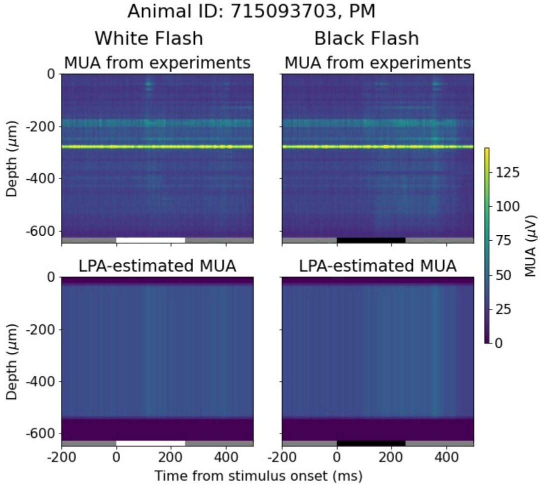

Session ID: 719161530

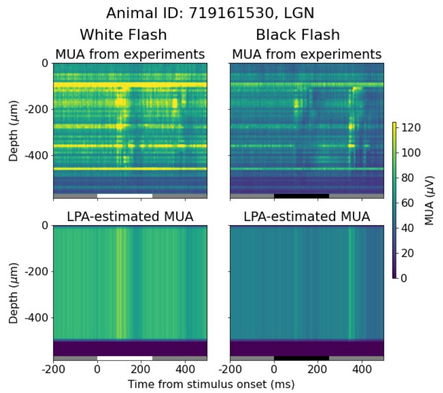

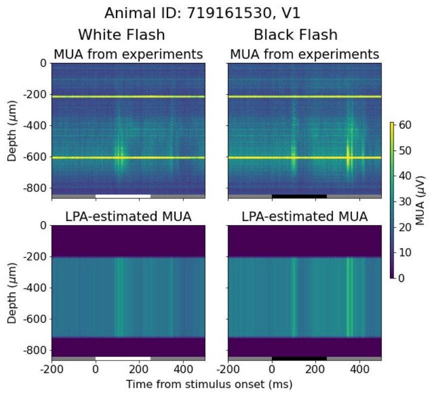

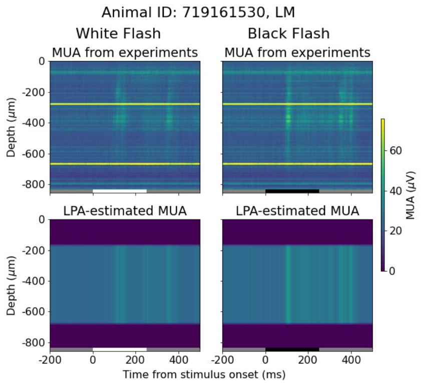

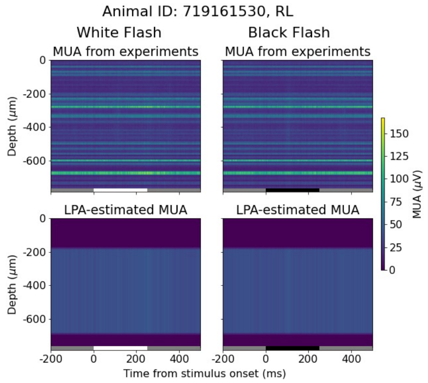

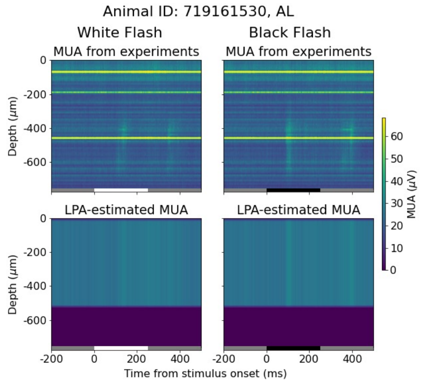

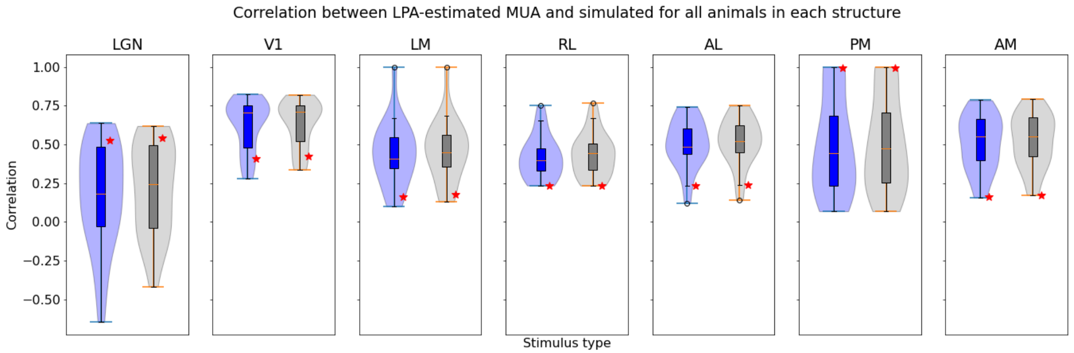

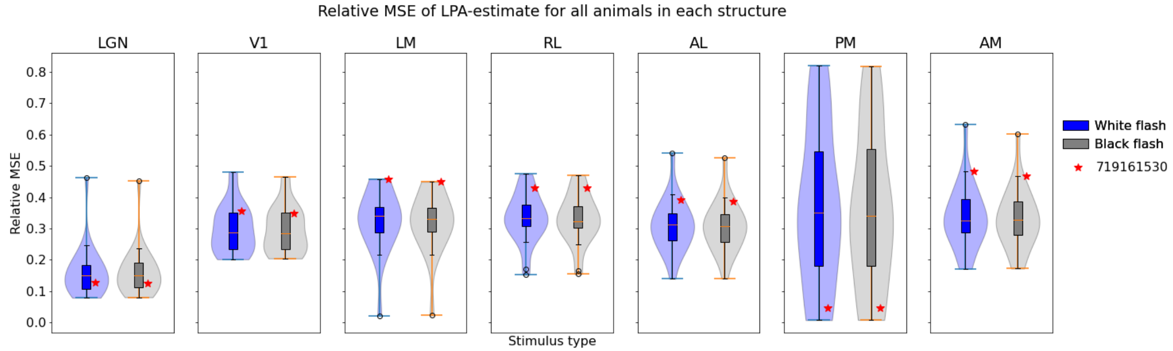

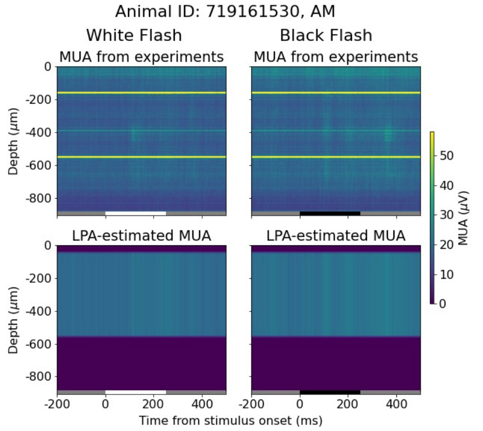

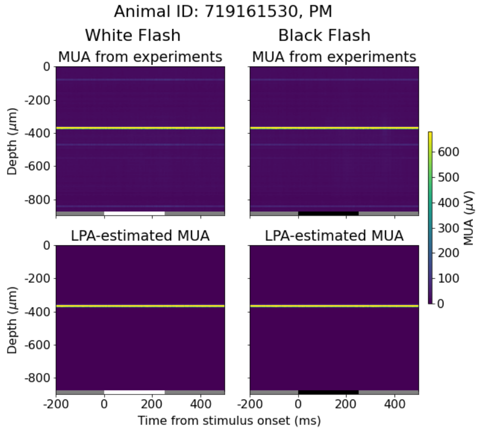

Session ID: 721173822

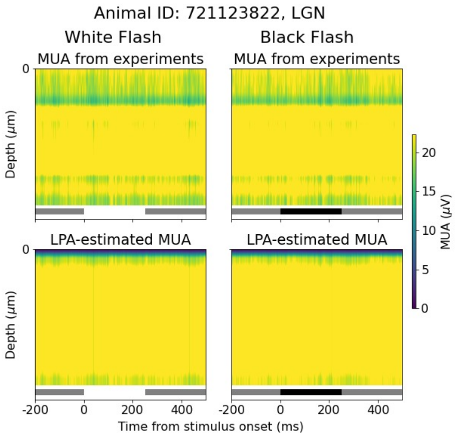

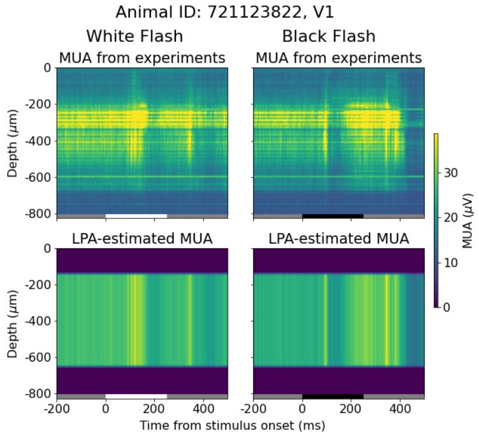

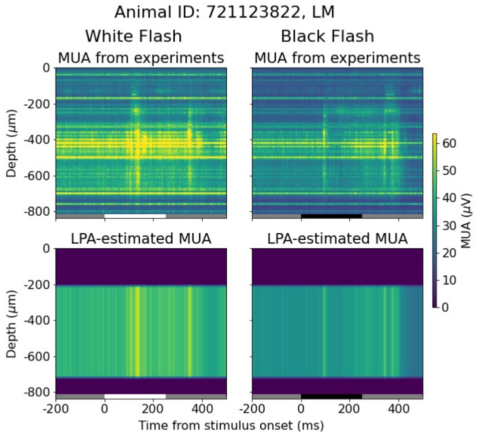

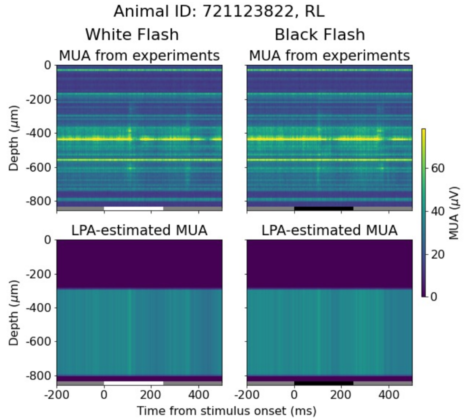

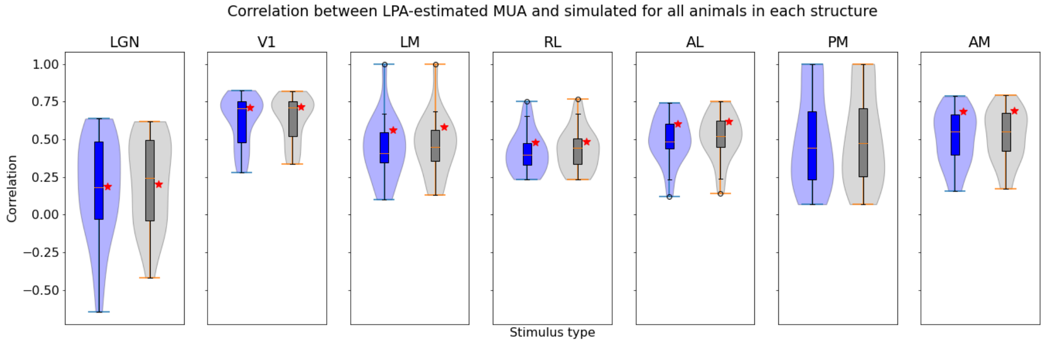

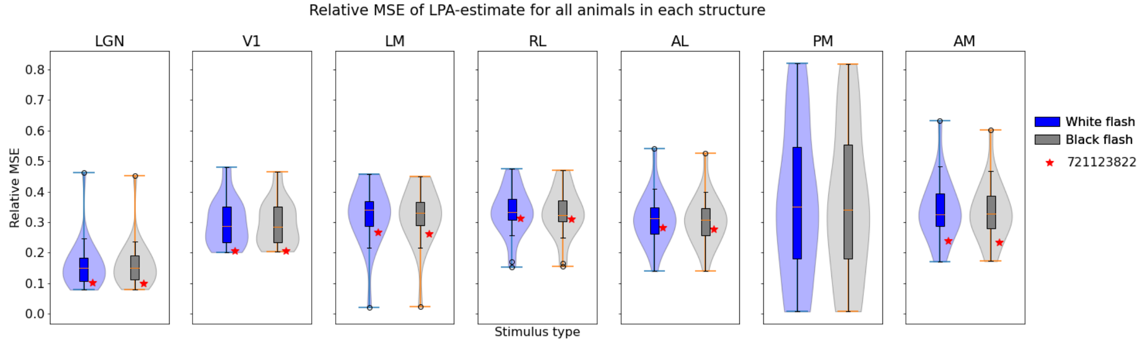

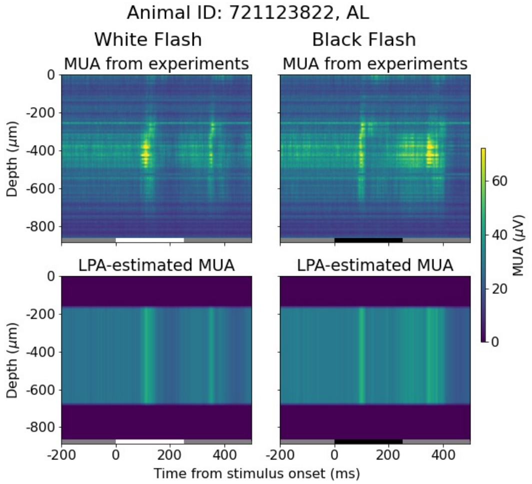

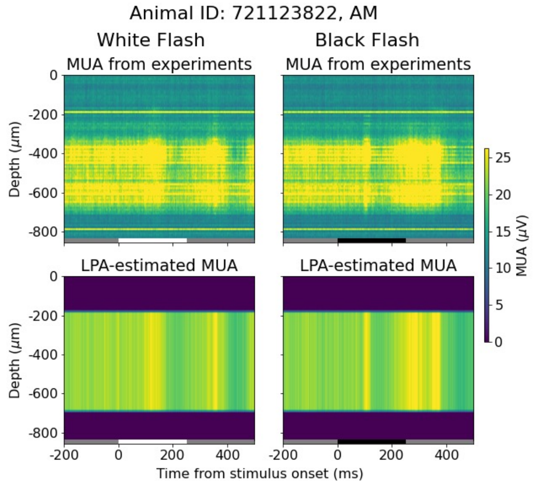

Session ID: 750749662

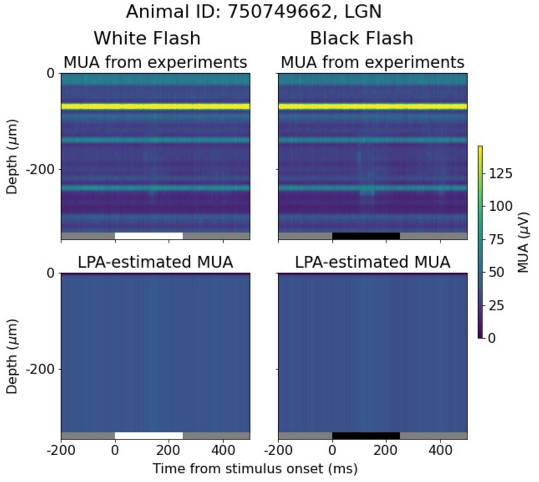

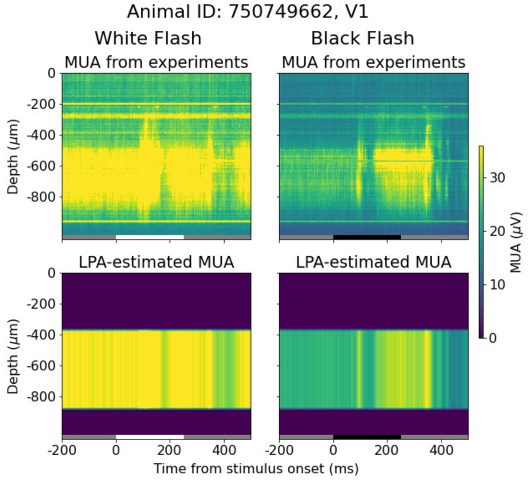

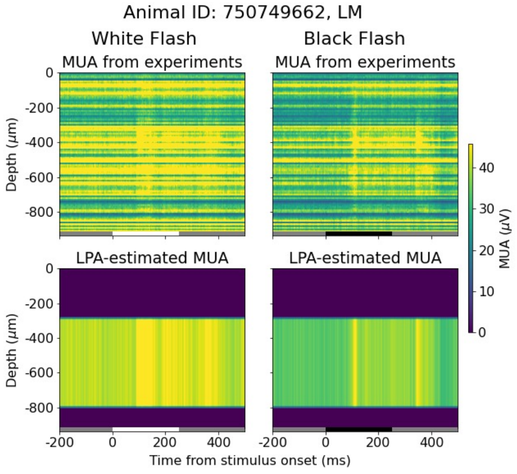

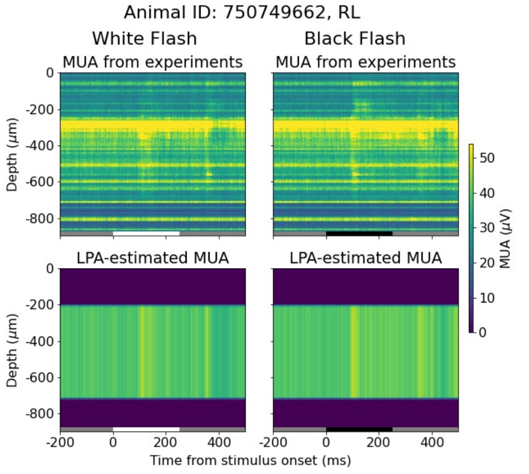

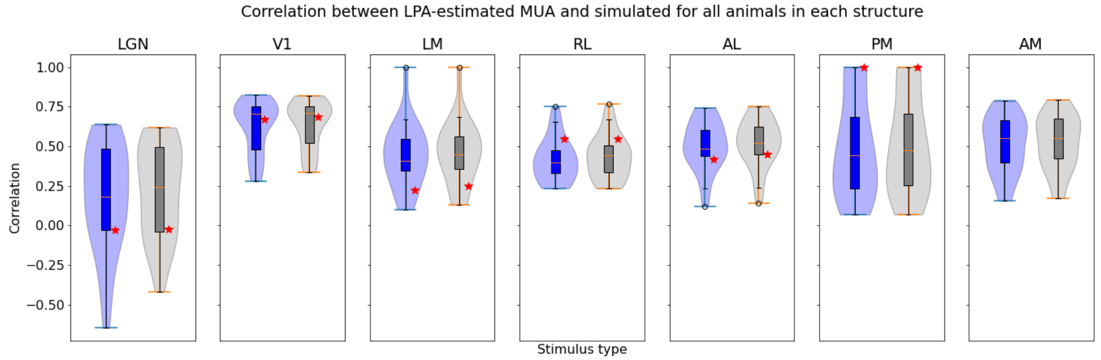

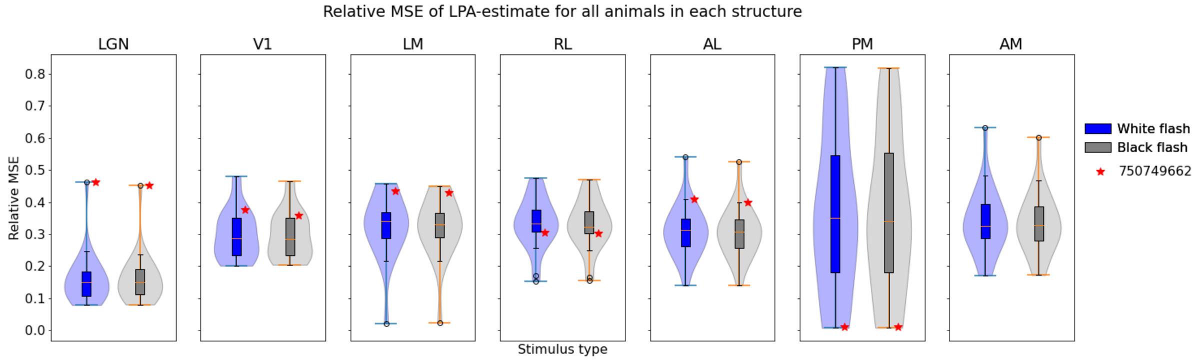

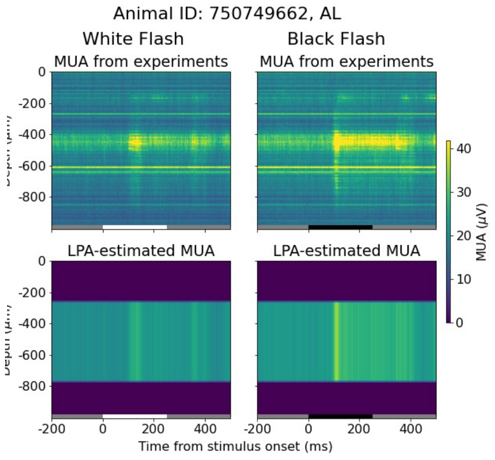

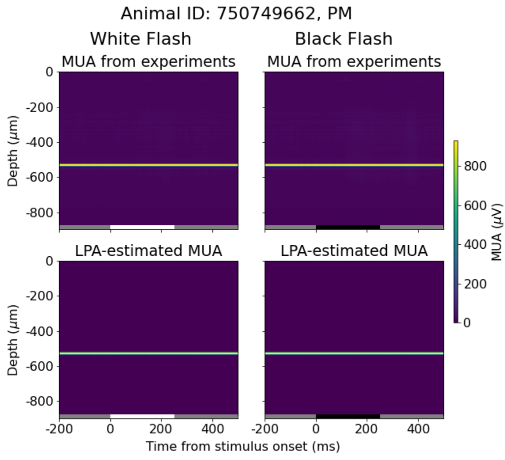

Session ID: 750749662

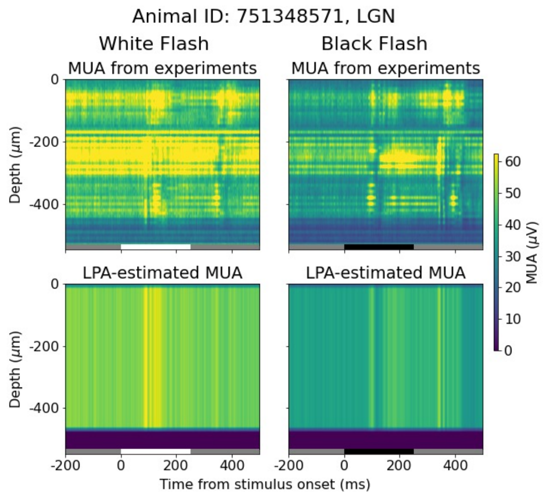

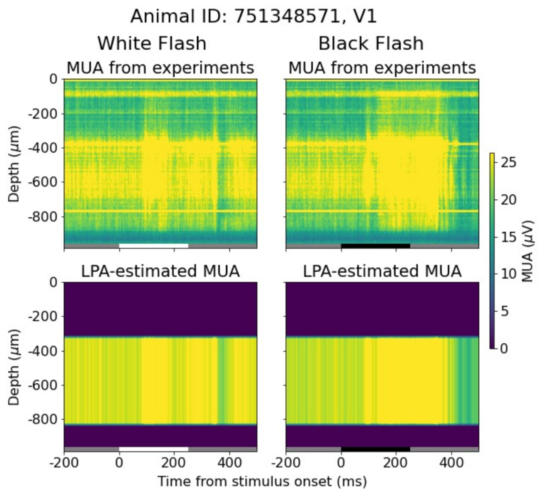

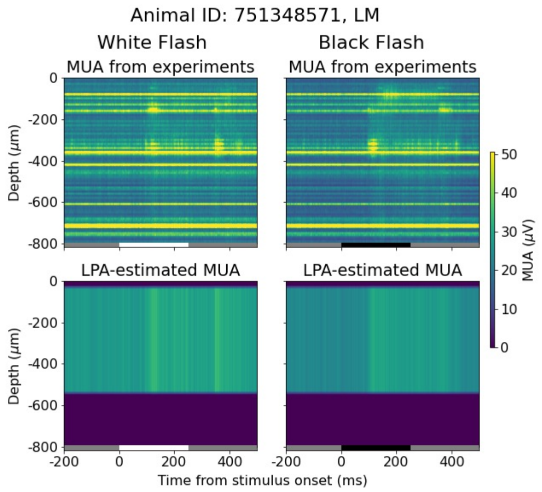

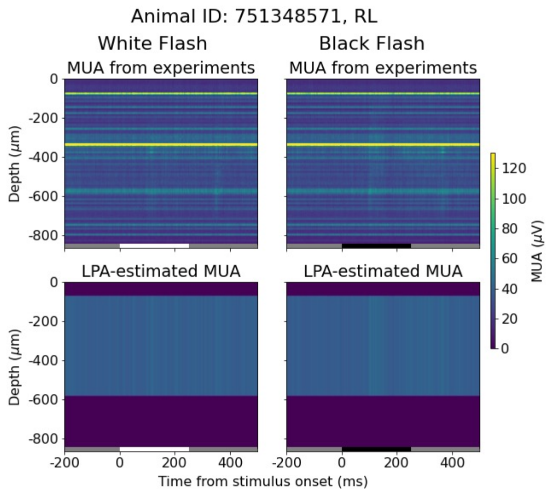

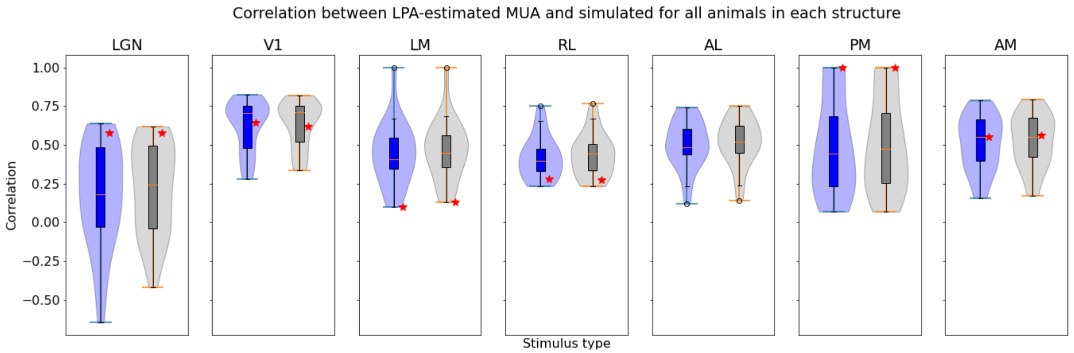

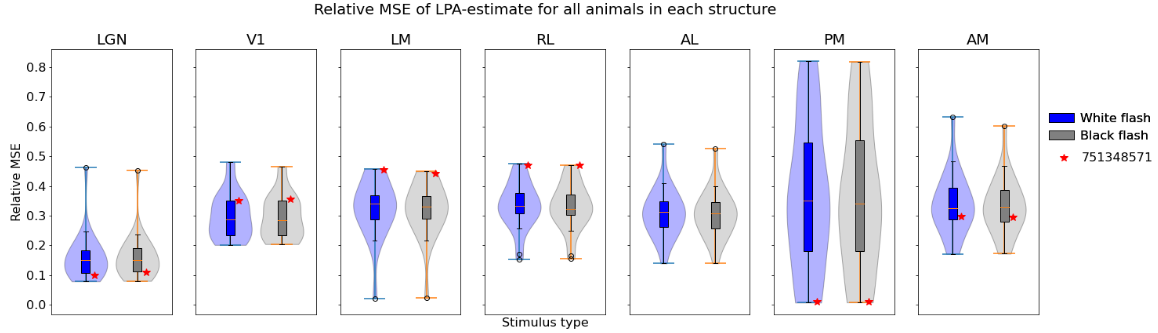

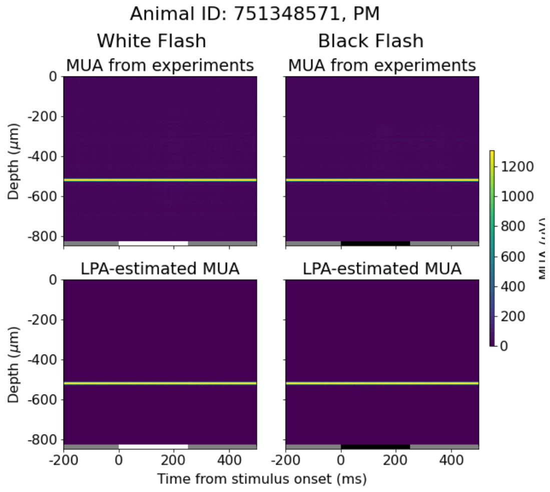

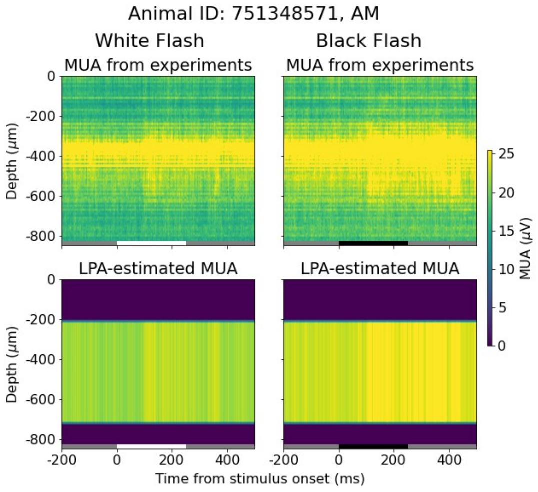

Session ID: 754312389

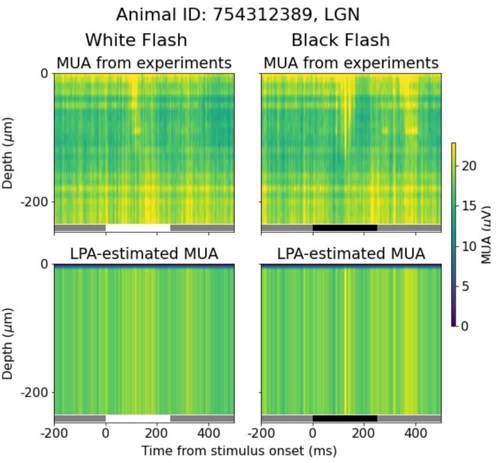

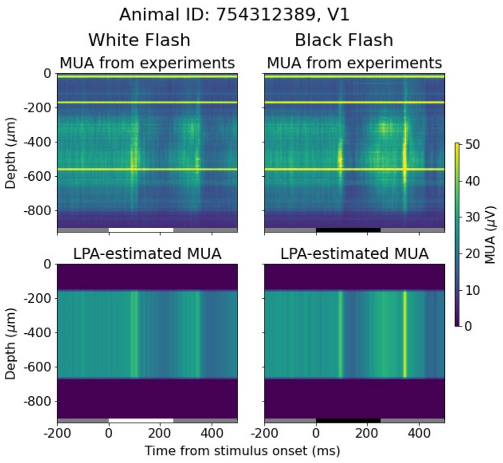

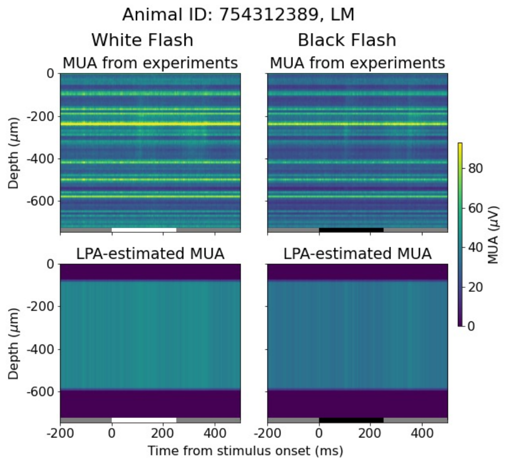

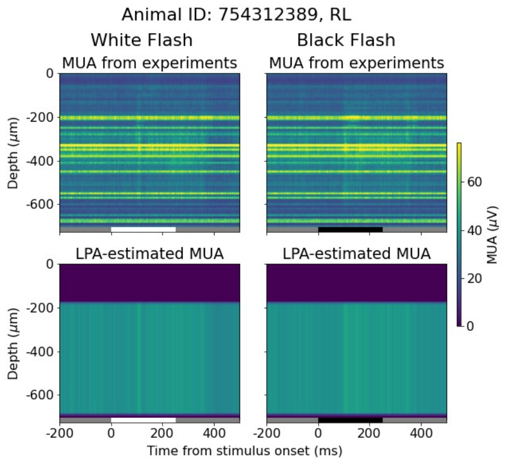

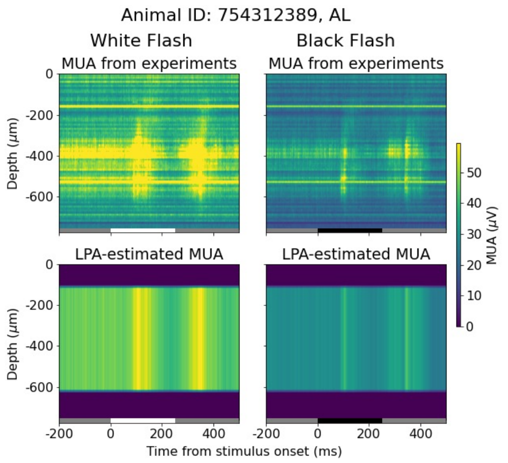

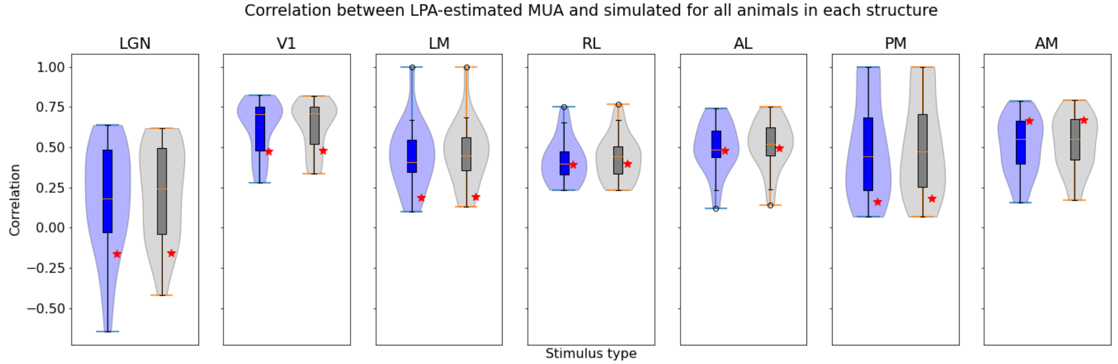

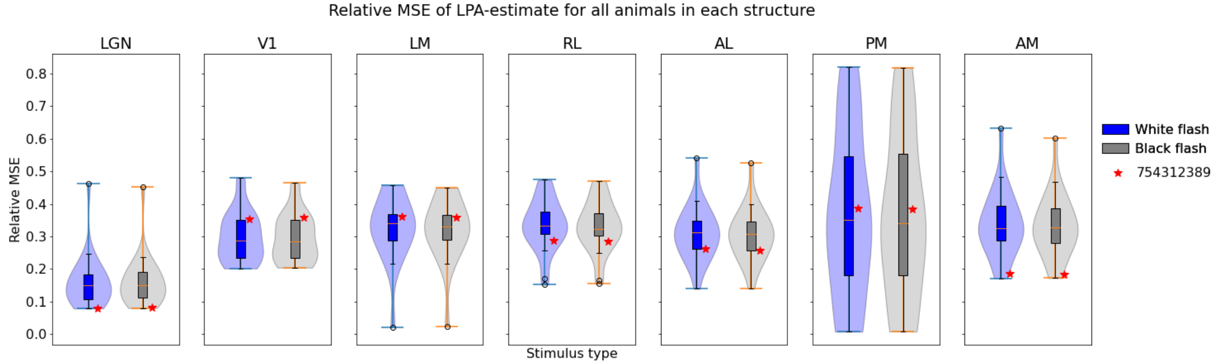

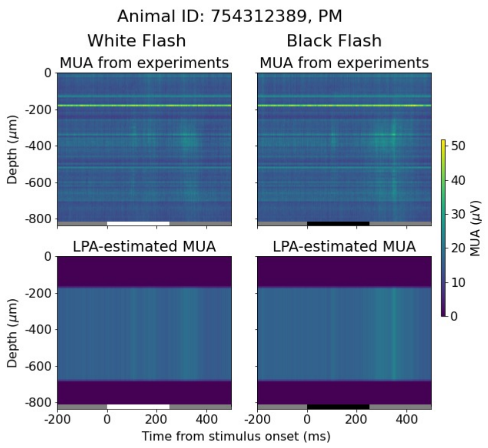

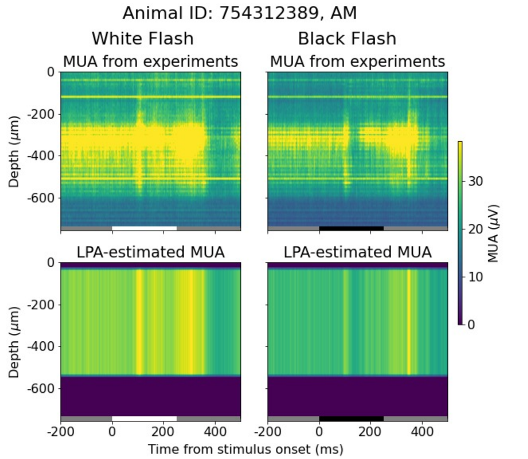

Session ID: 754829445

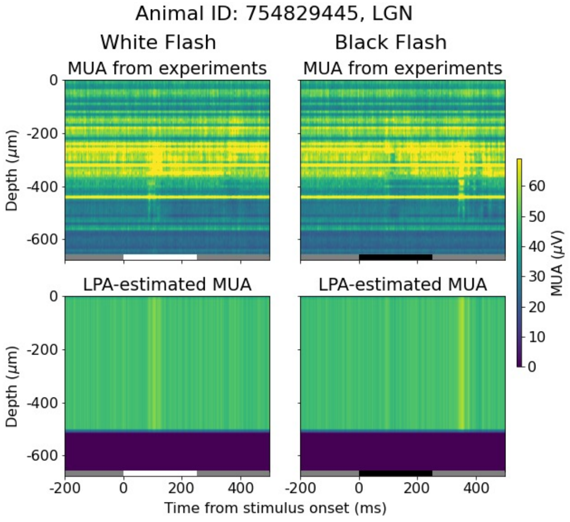

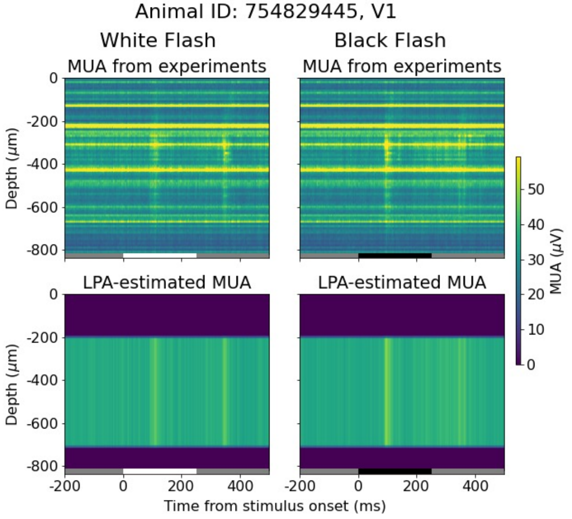

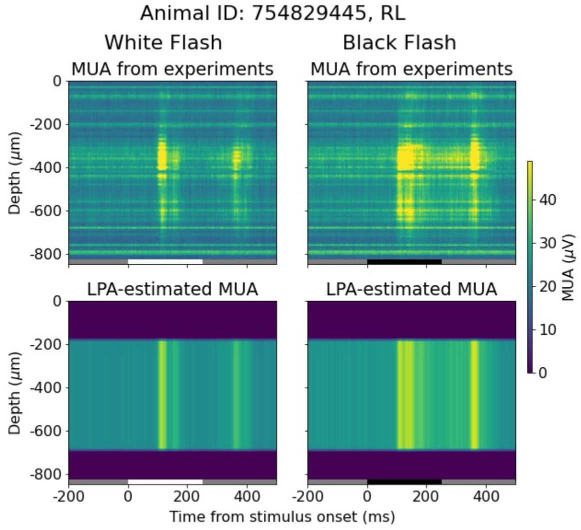

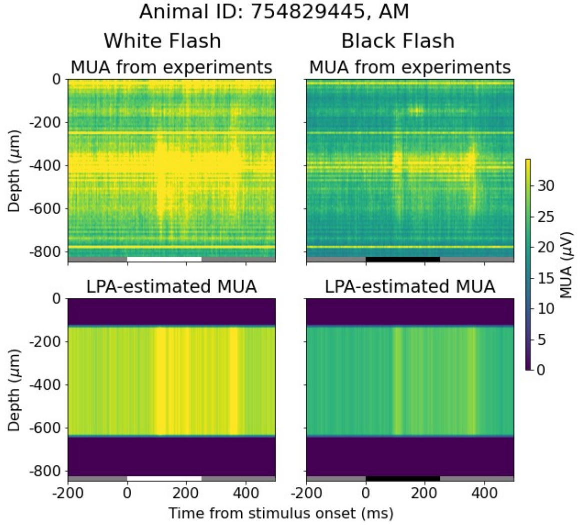

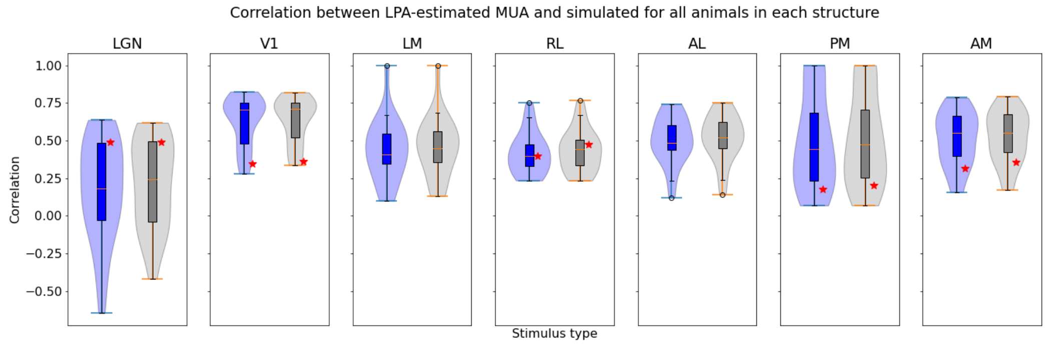

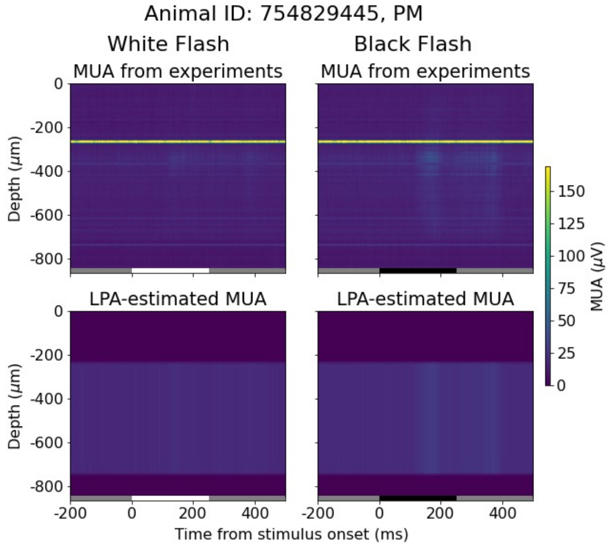

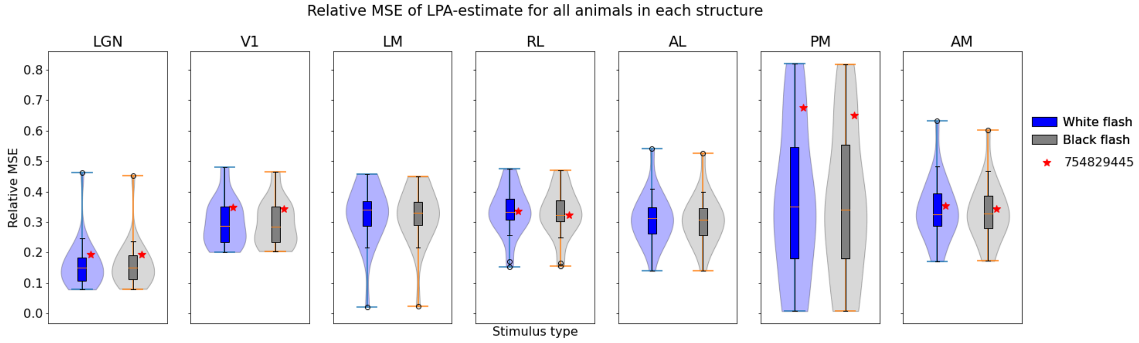

Session ID: 755434585

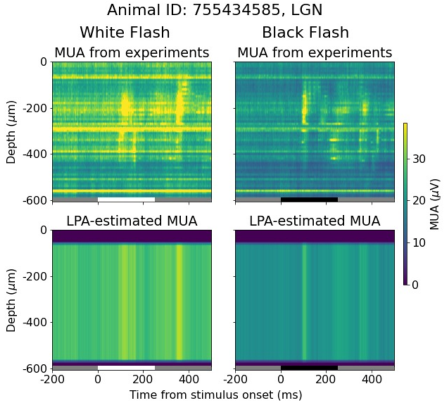

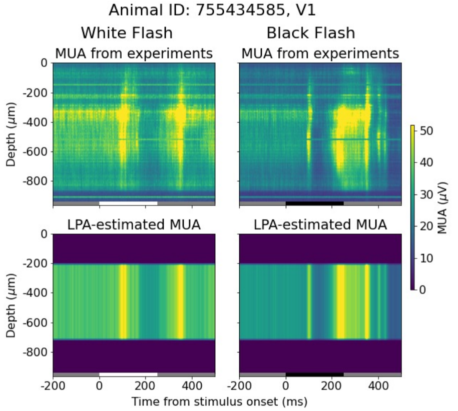

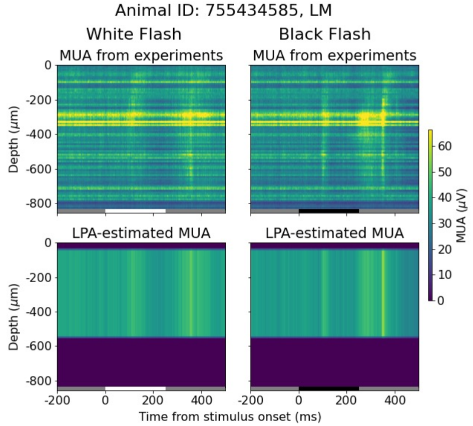

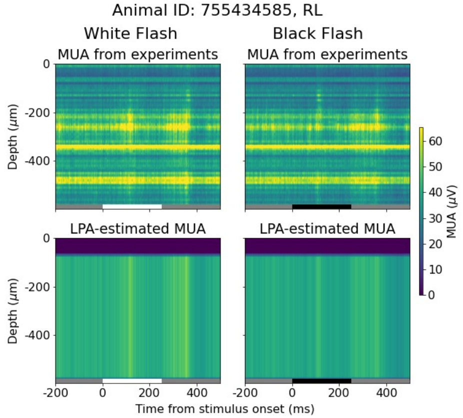

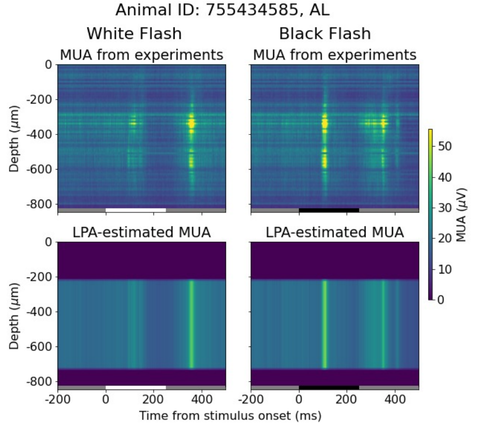

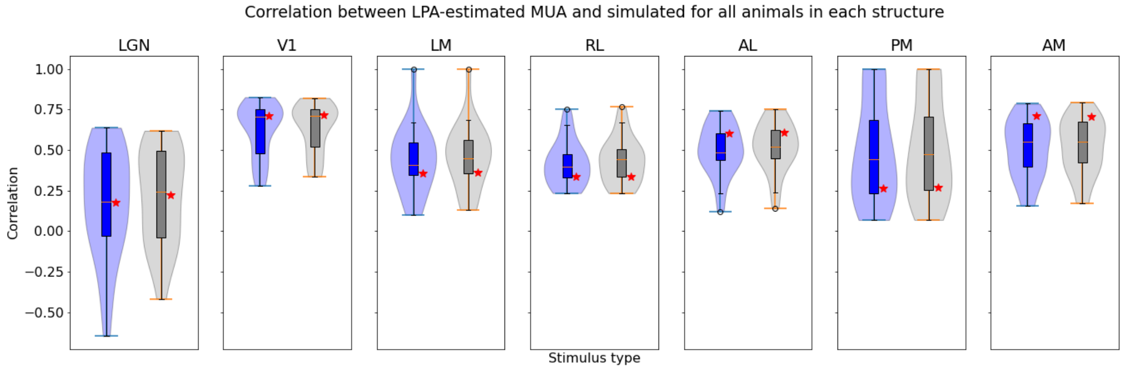

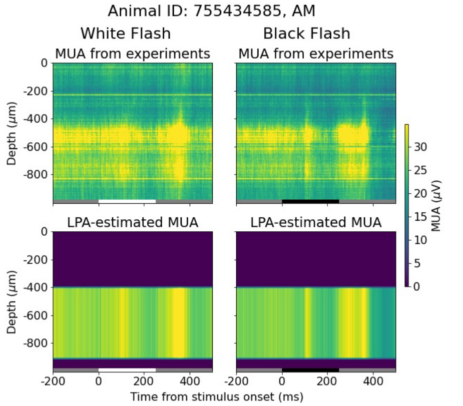

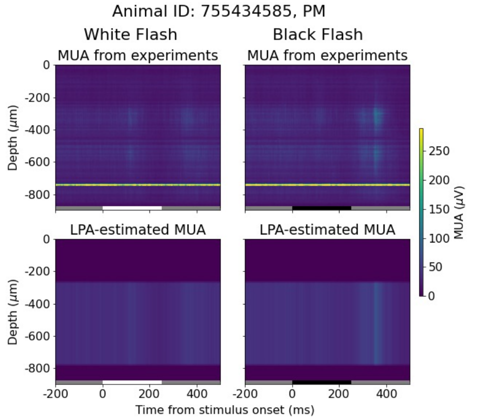

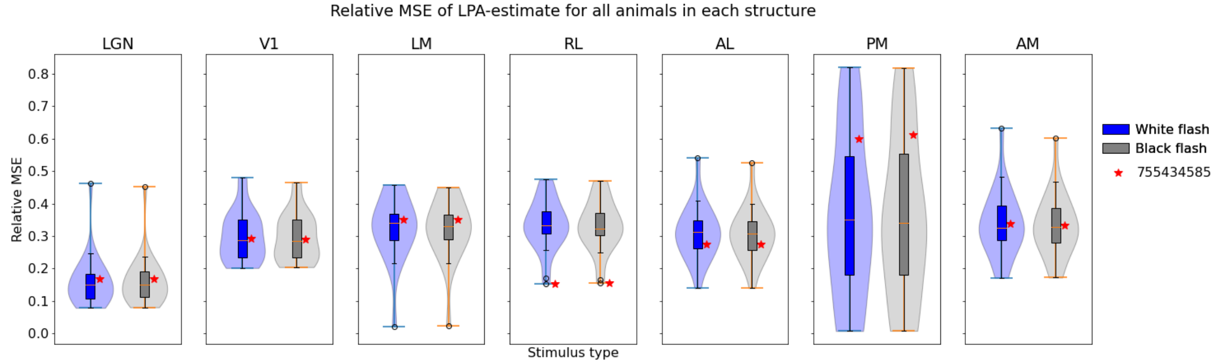

Session ID: 756029989

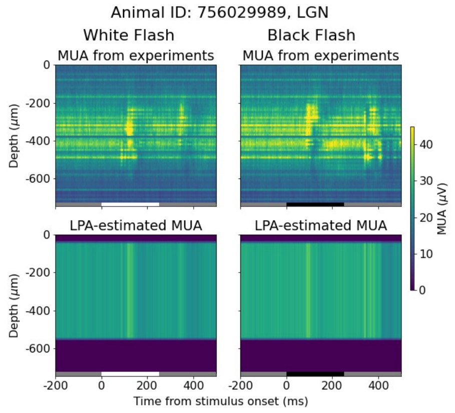

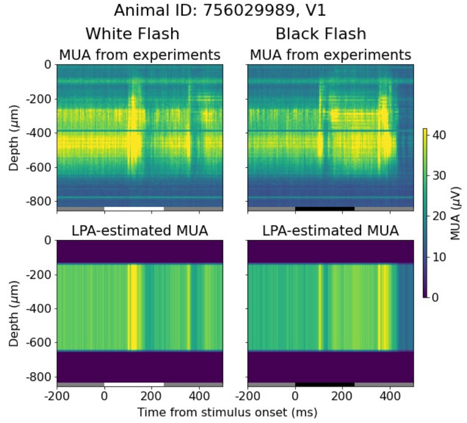

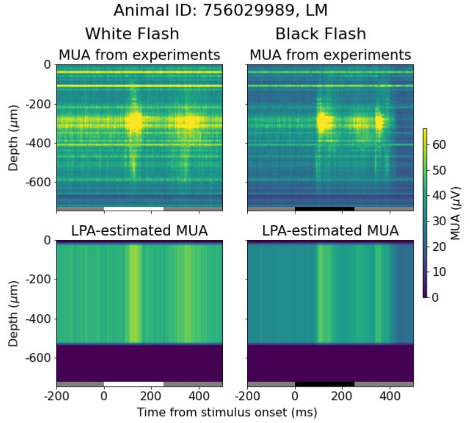

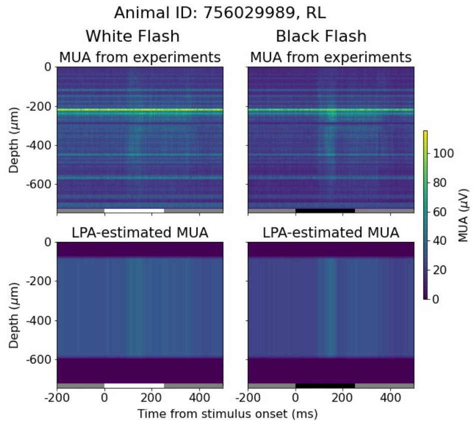

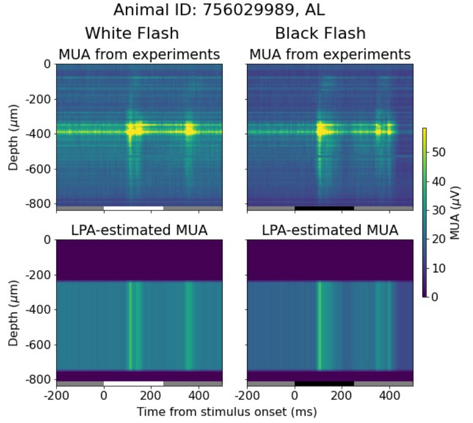

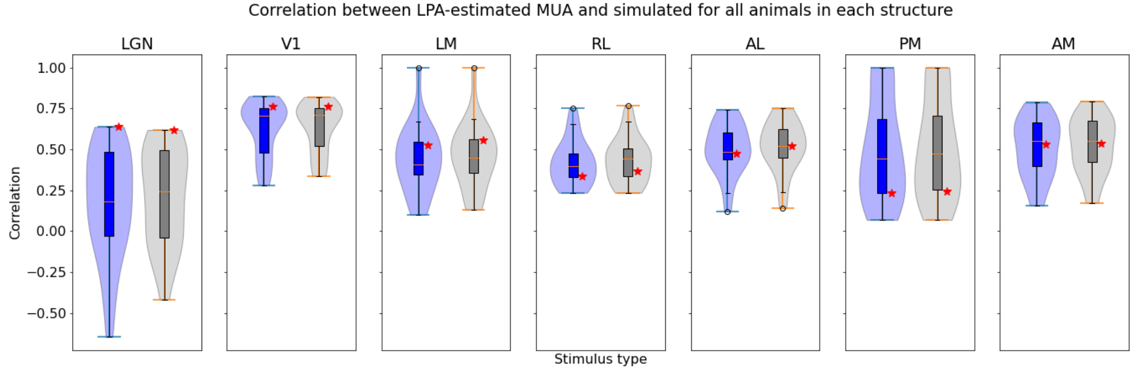

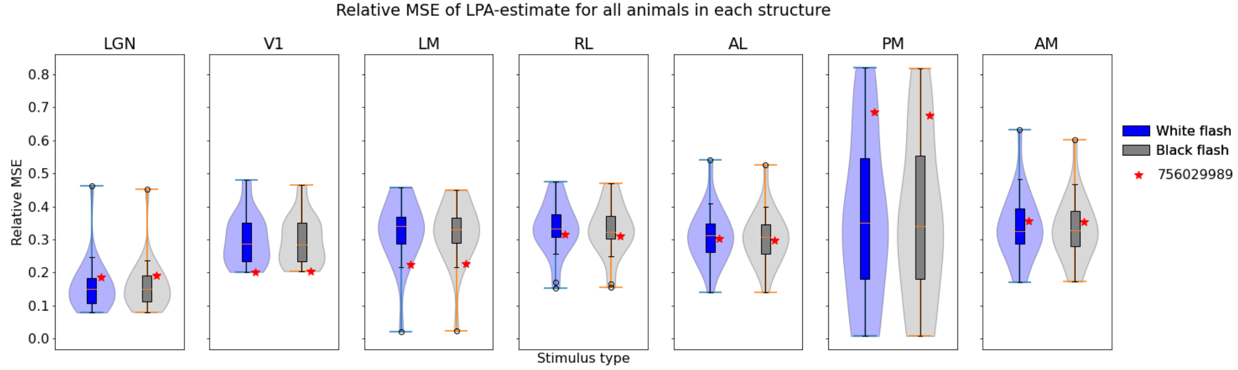

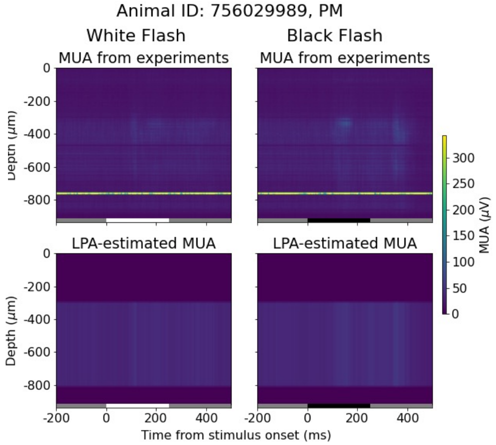

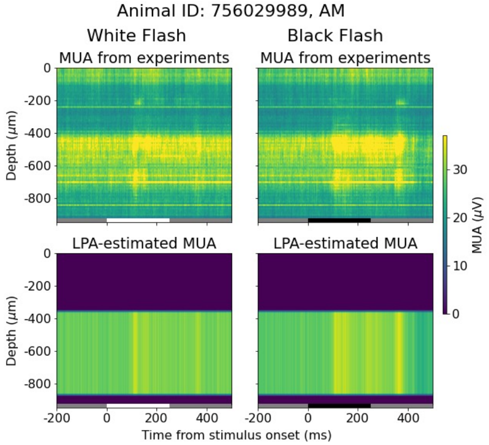

Session ID: 757970808

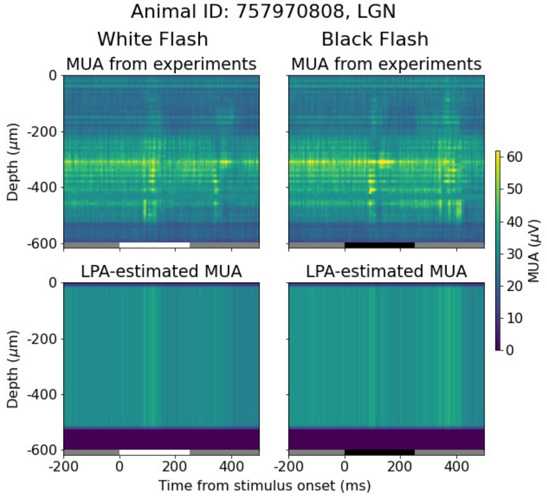

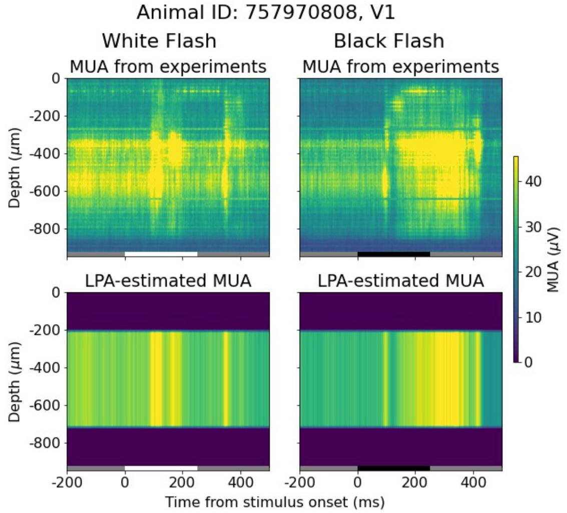

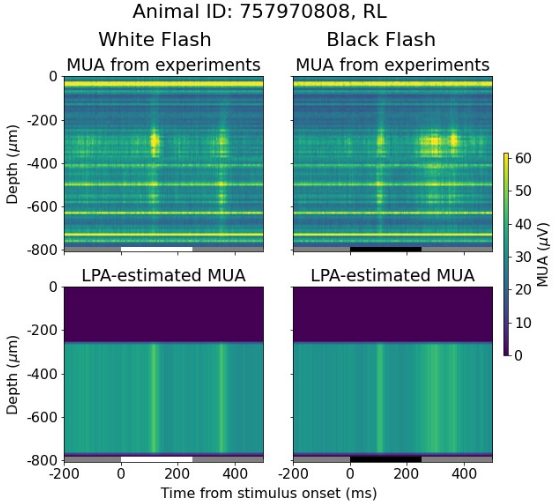

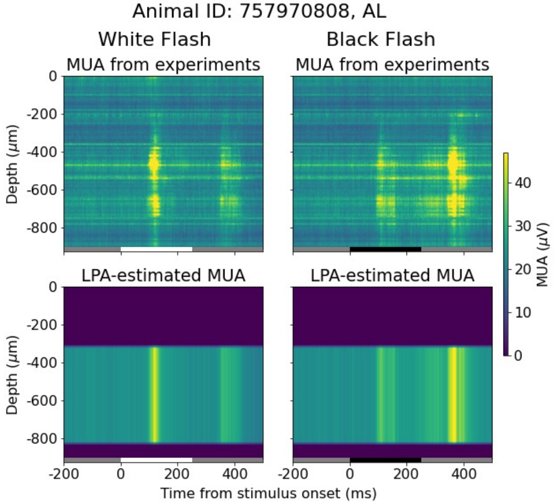

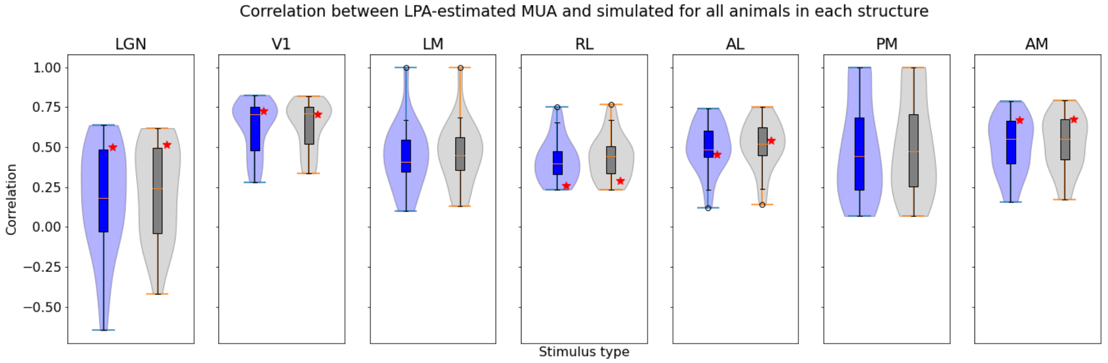

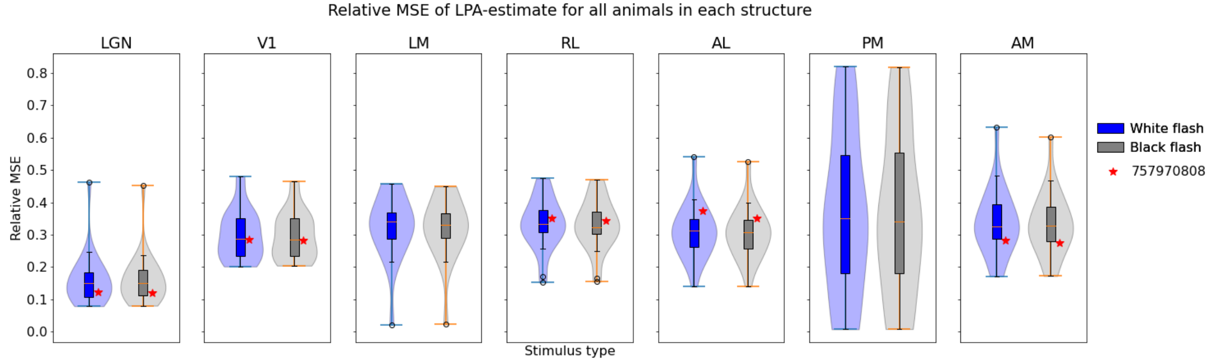

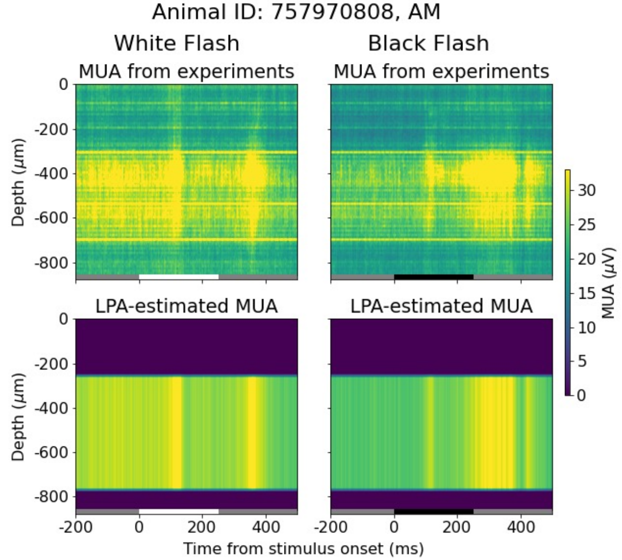

Session ID: 759883607

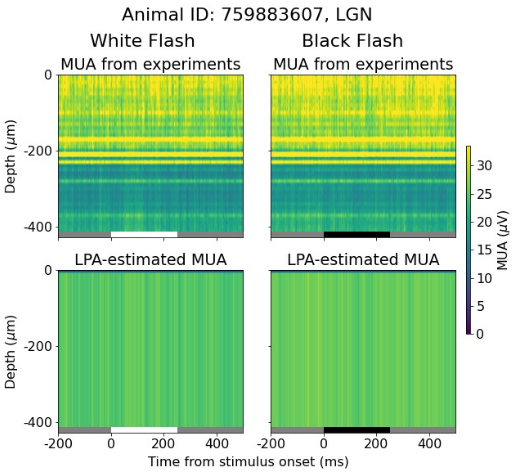

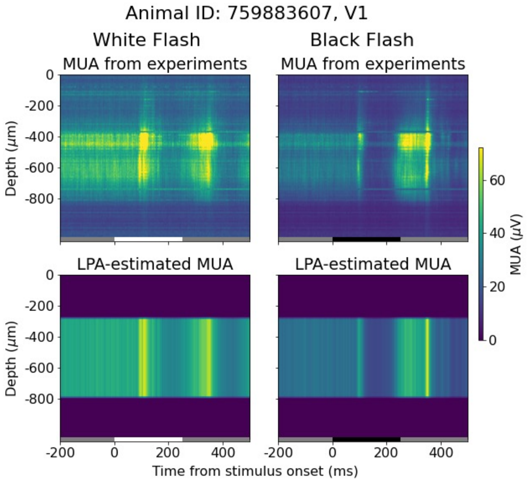

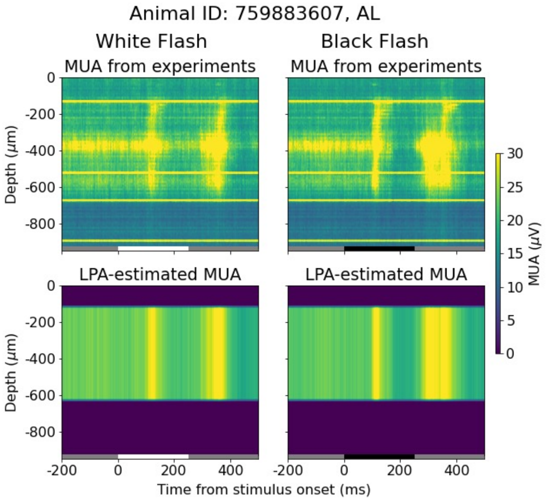

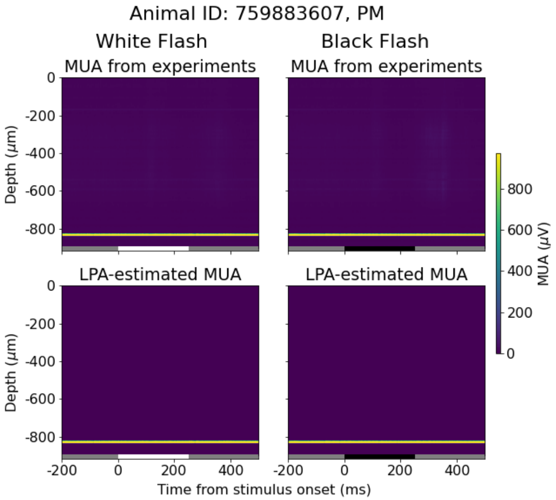

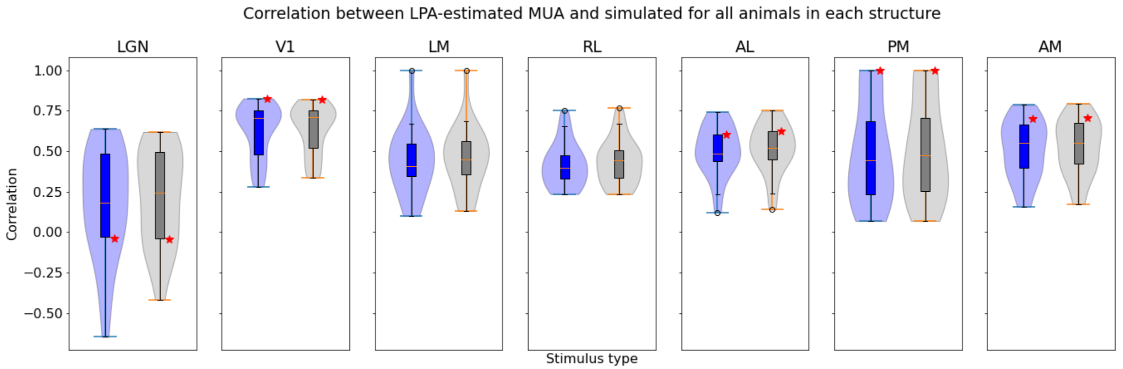

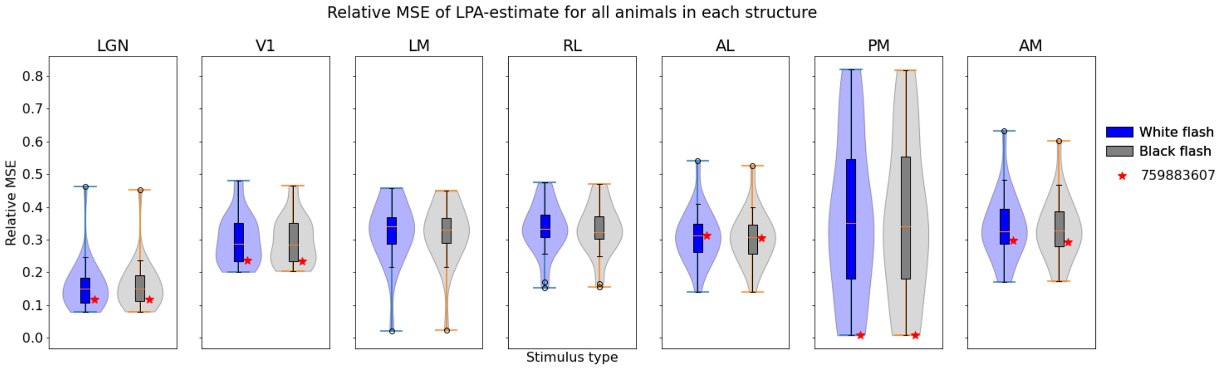

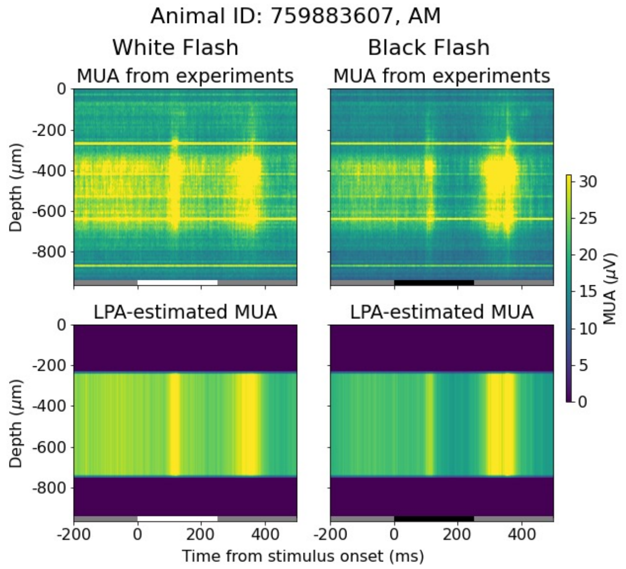

Session ID: 760345702

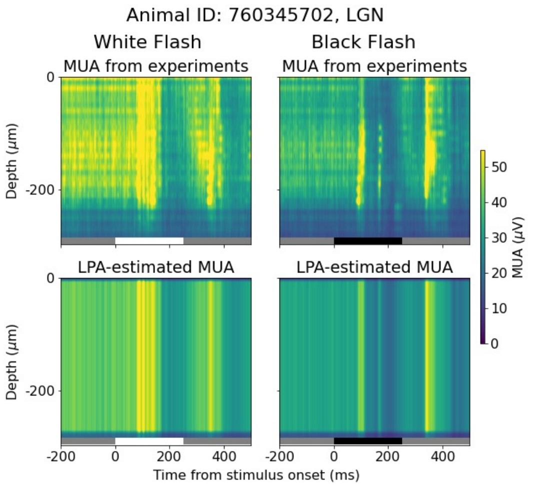

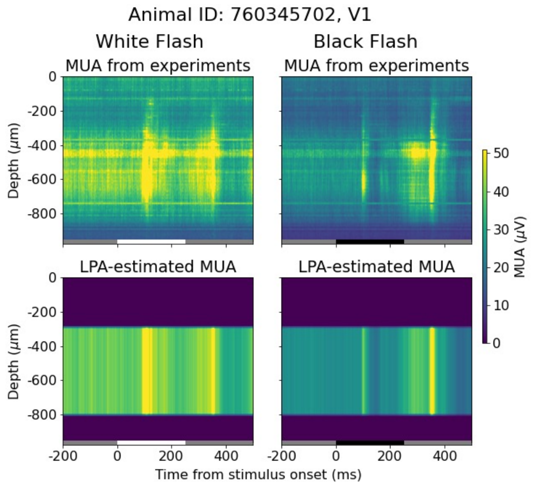

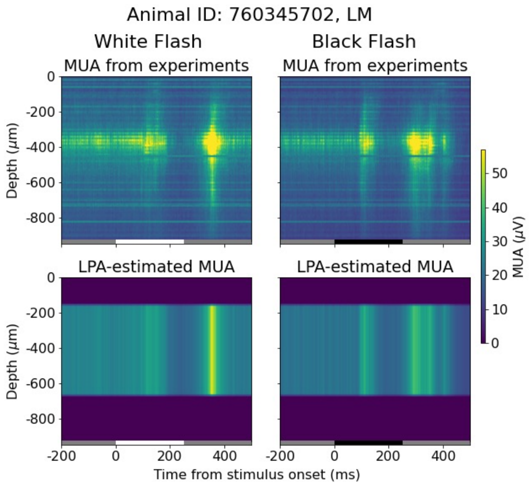

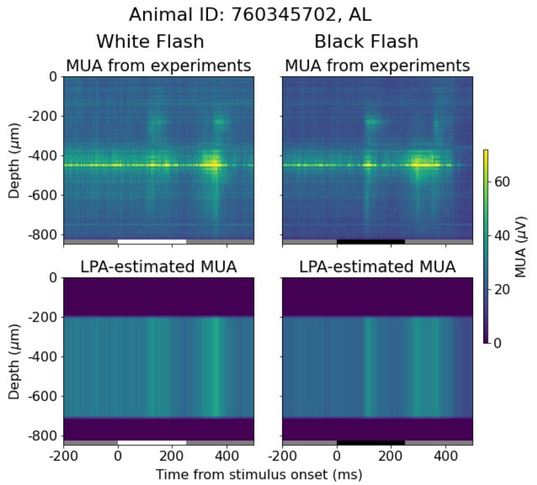

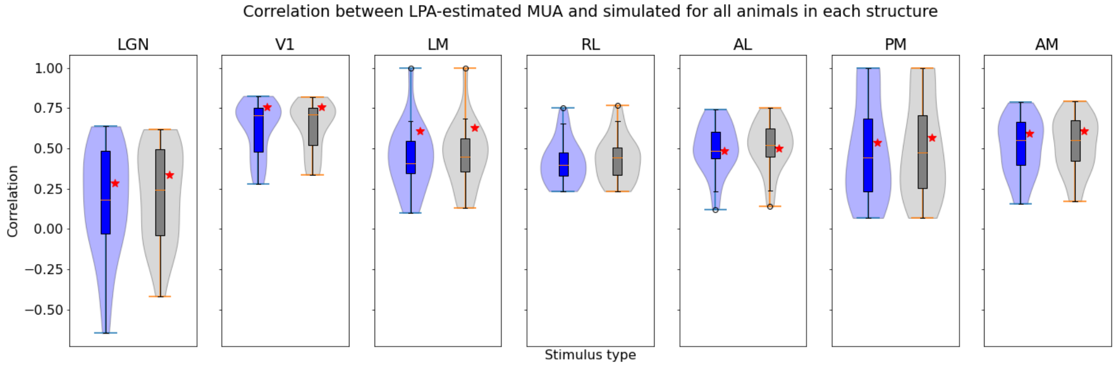

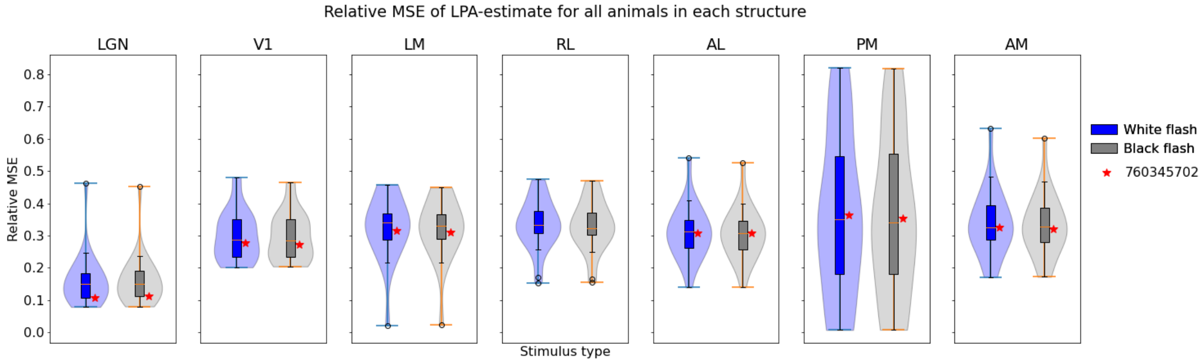

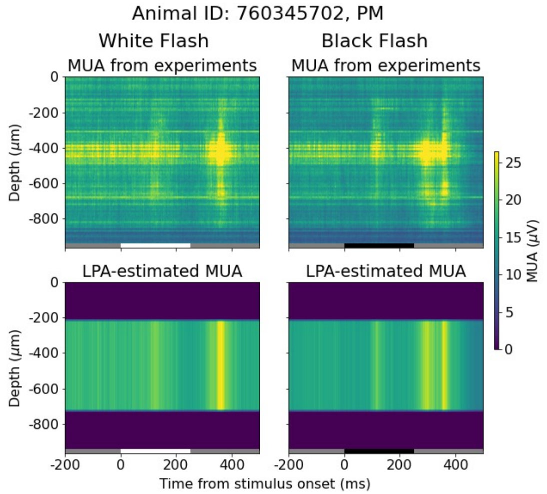

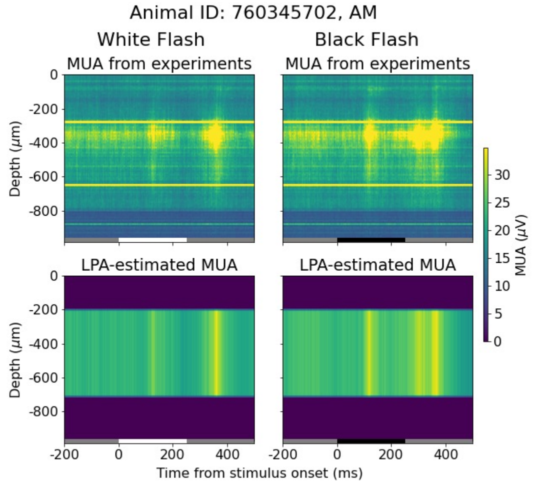

Session ID: 763673393

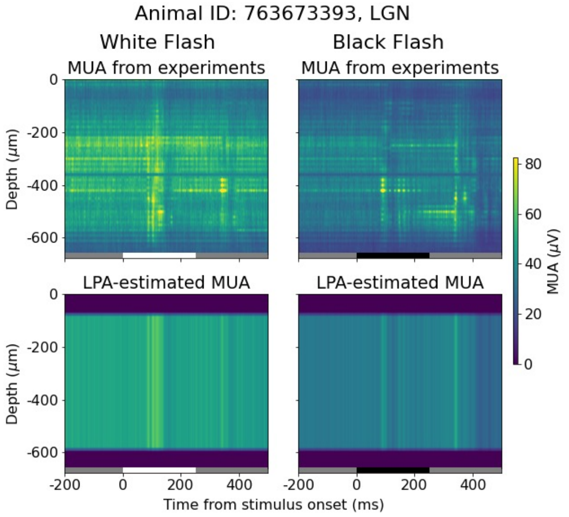

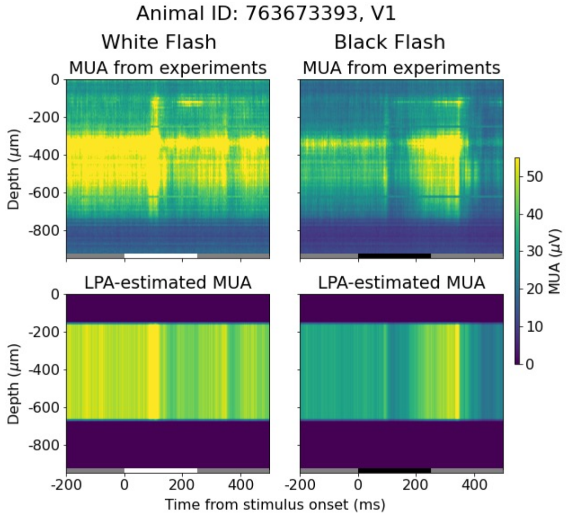

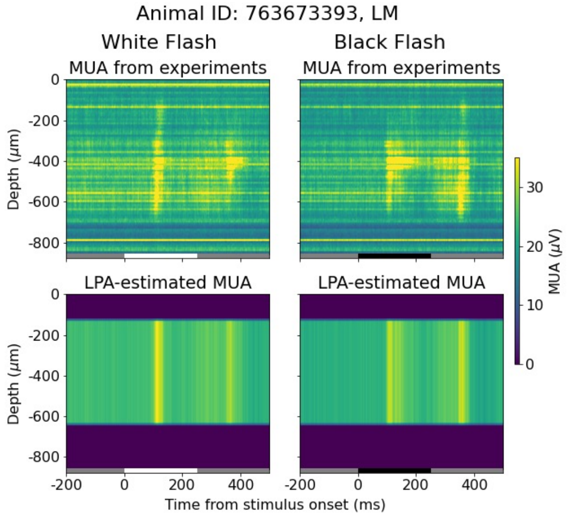

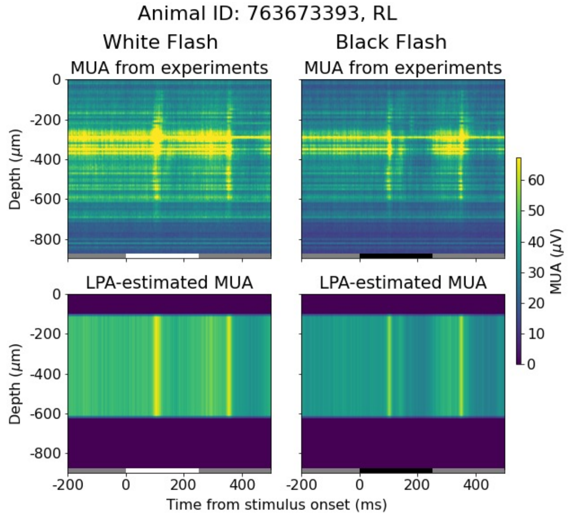

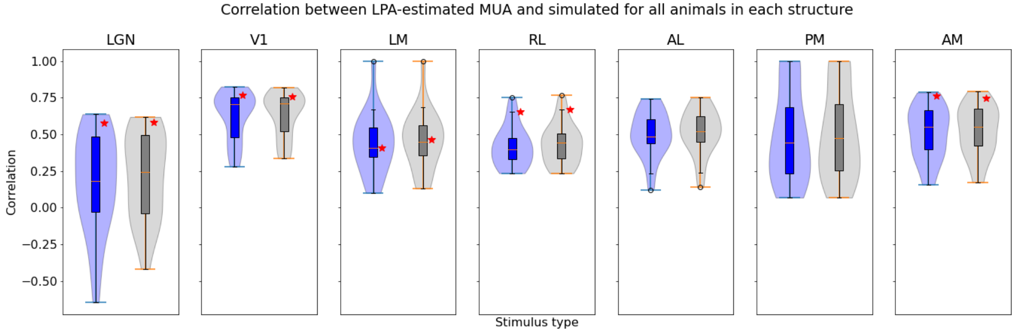

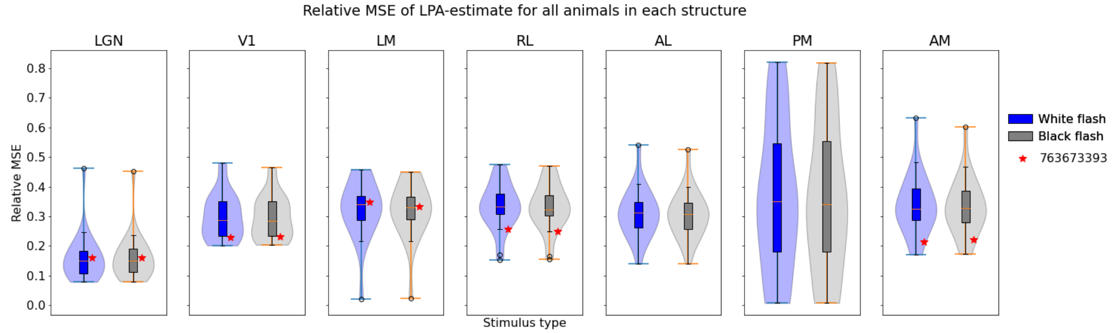

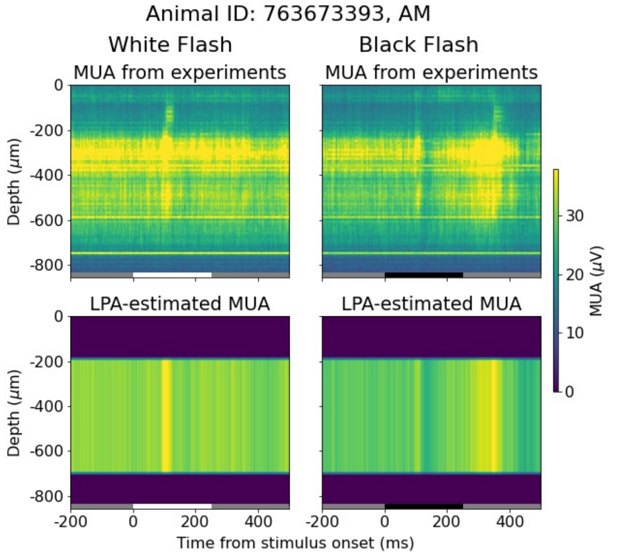

Session ID 766640955:

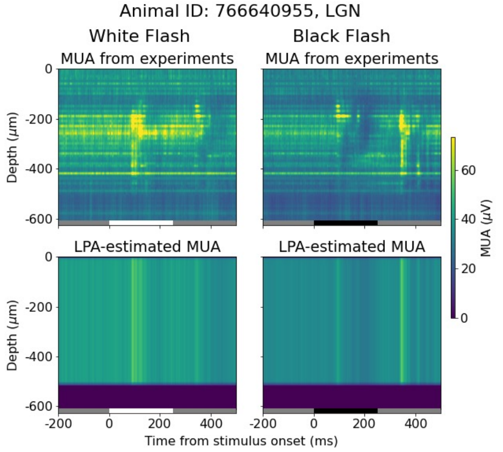

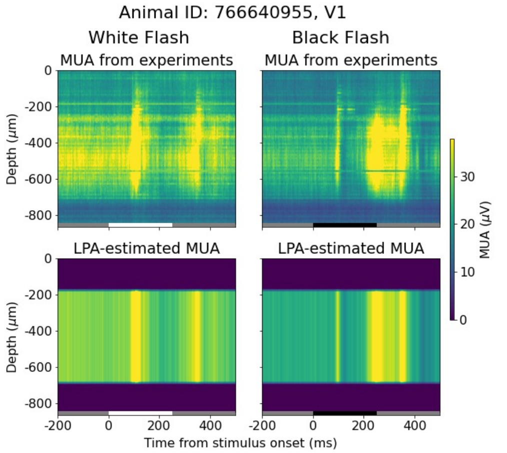

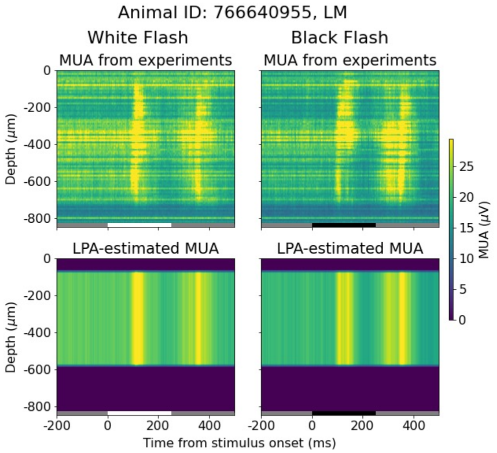

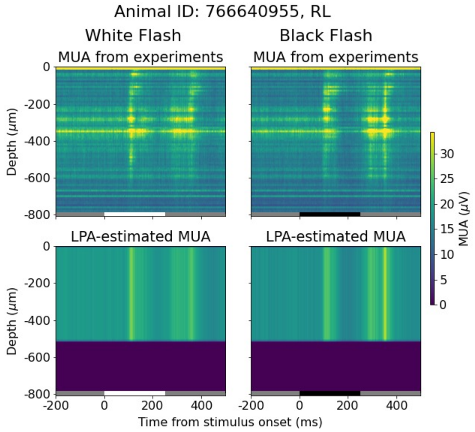

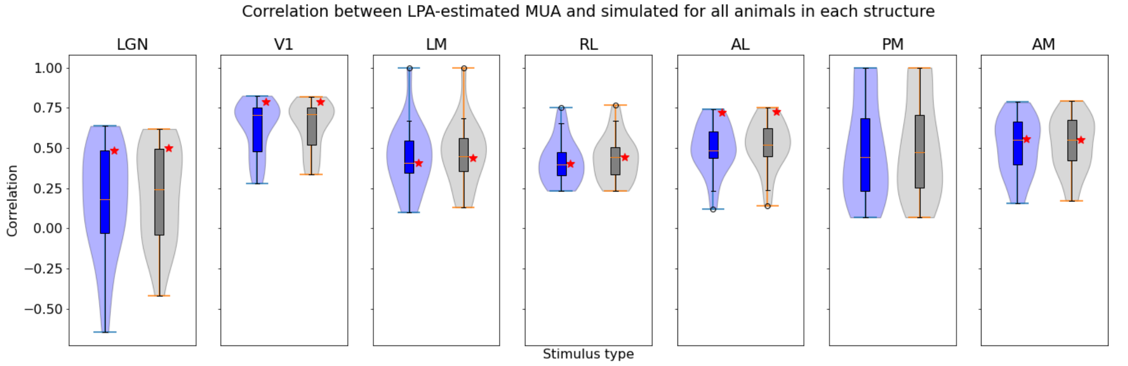

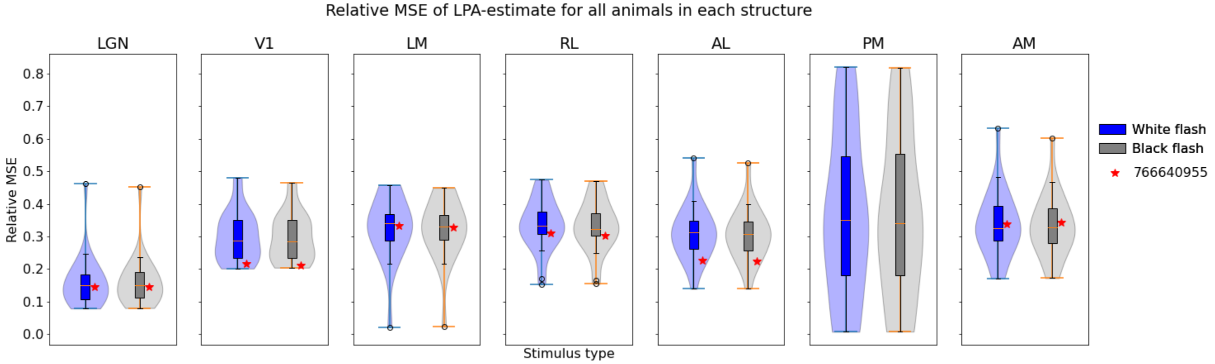

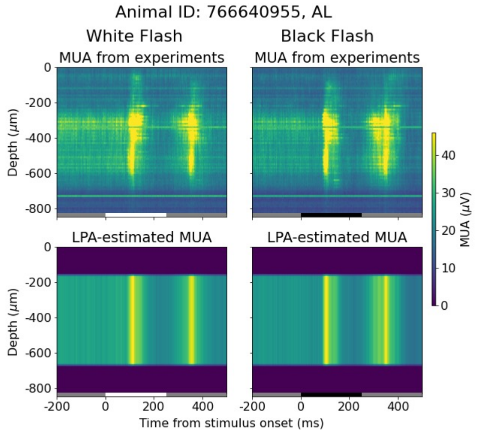

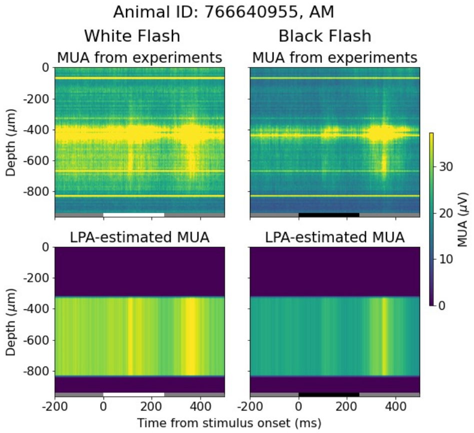

Session ID: 768515987

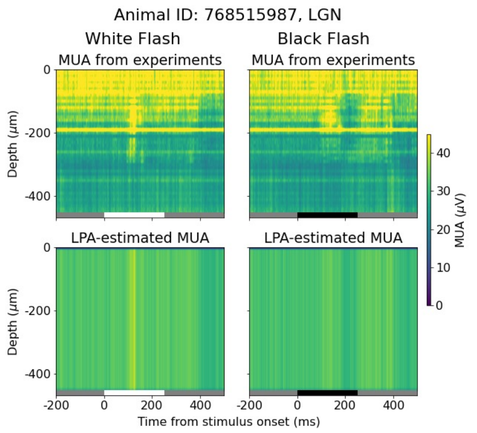

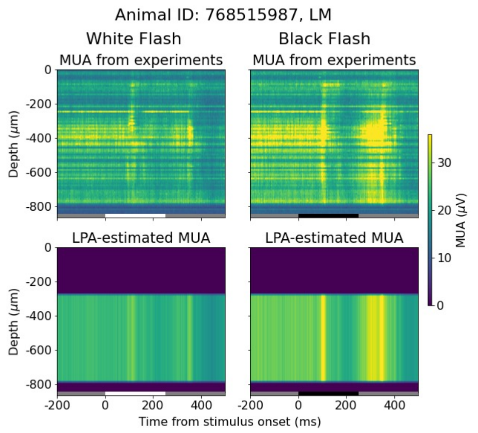

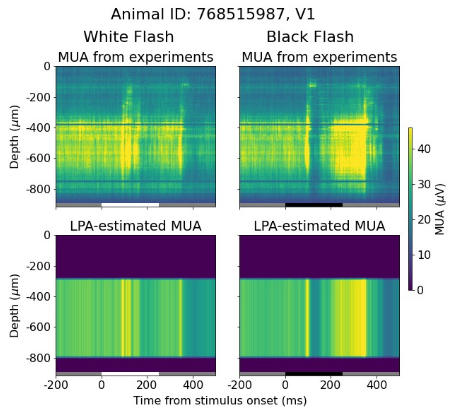

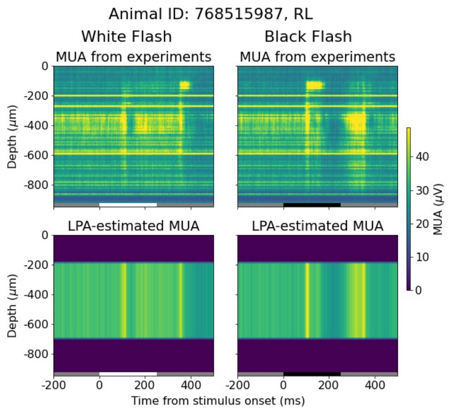

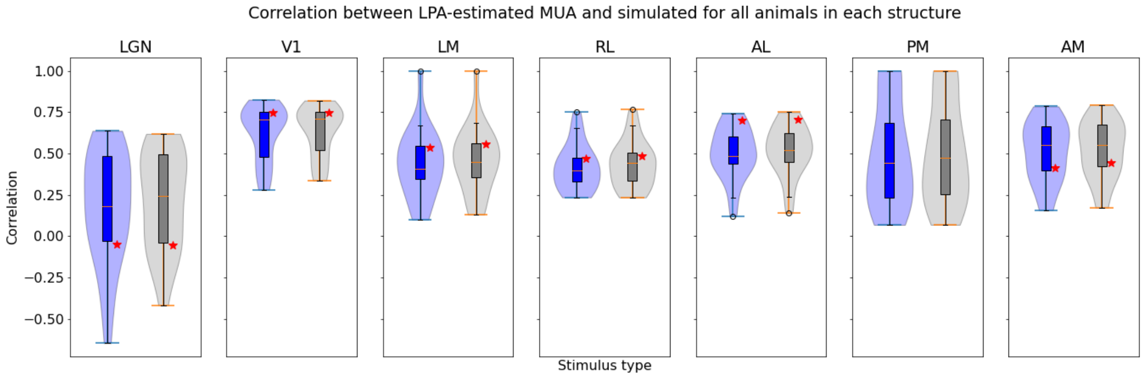

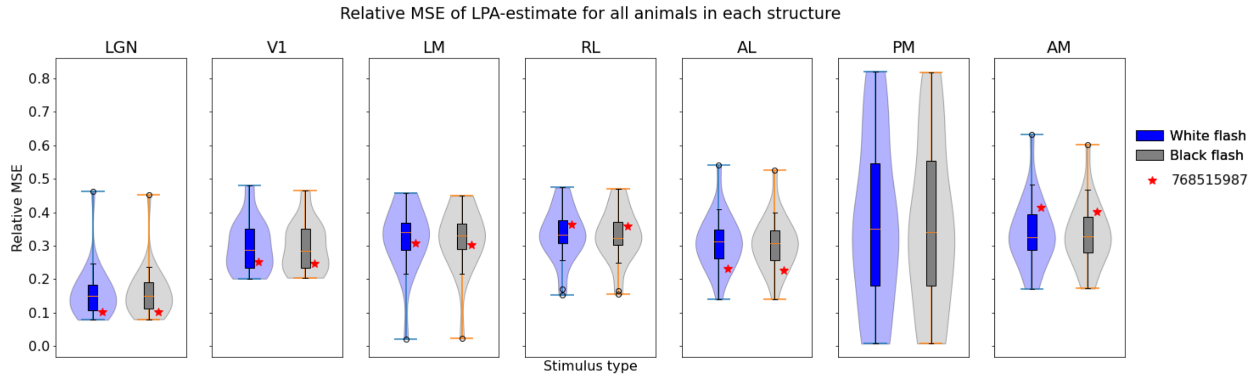

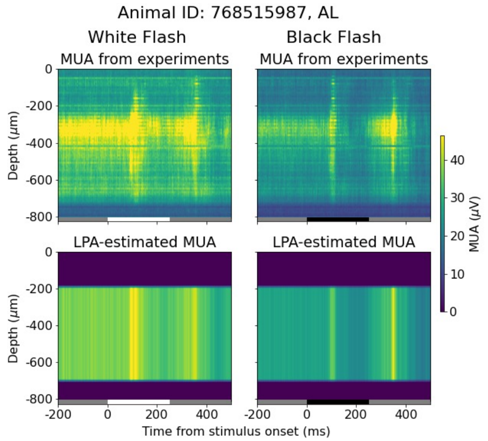

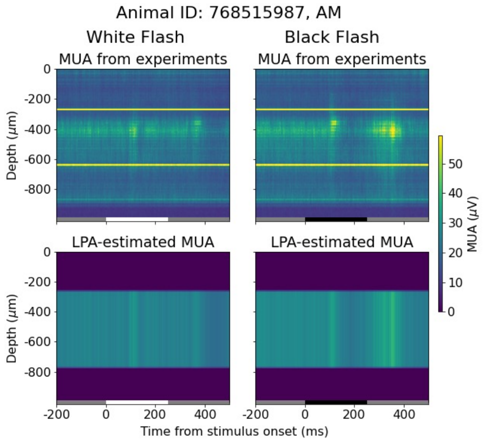

Session ID: 771160300

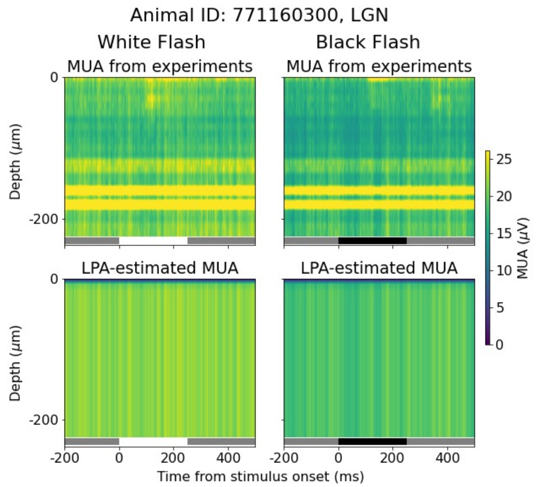

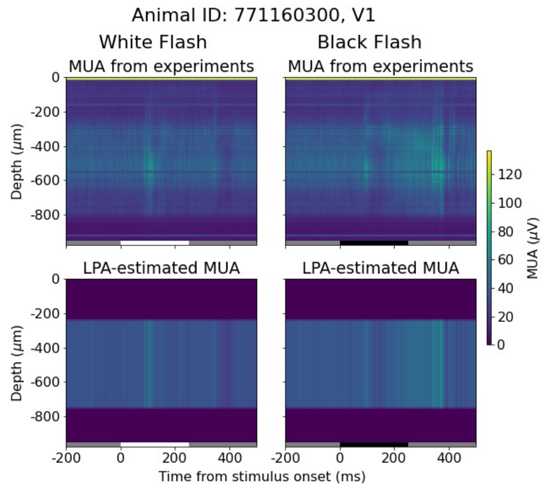

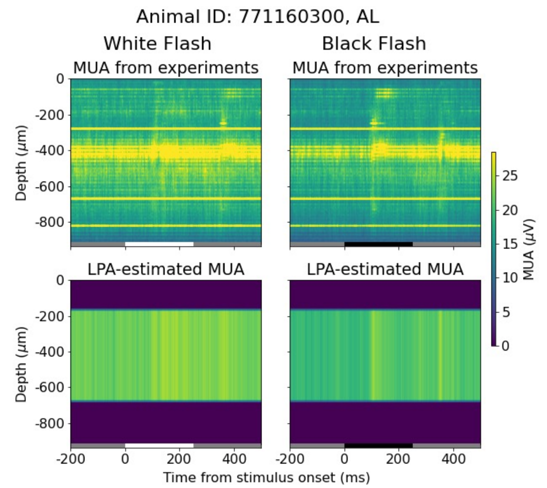

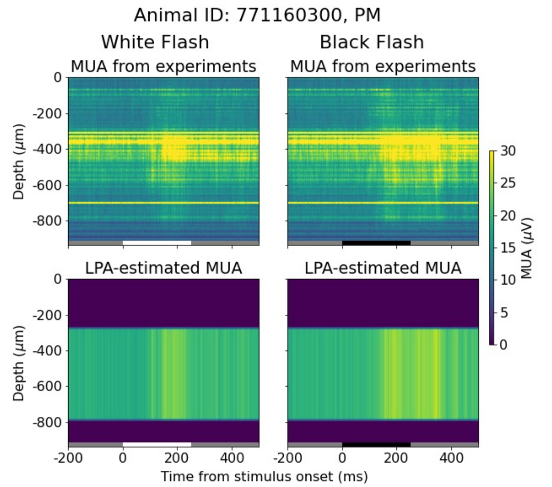

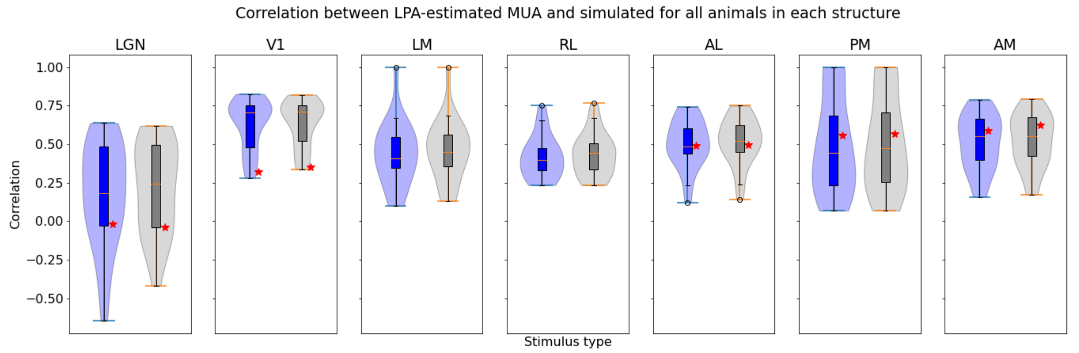

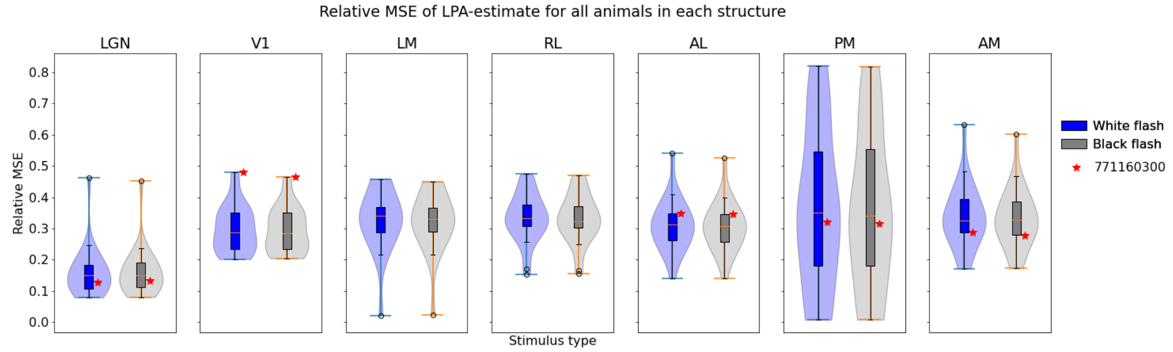

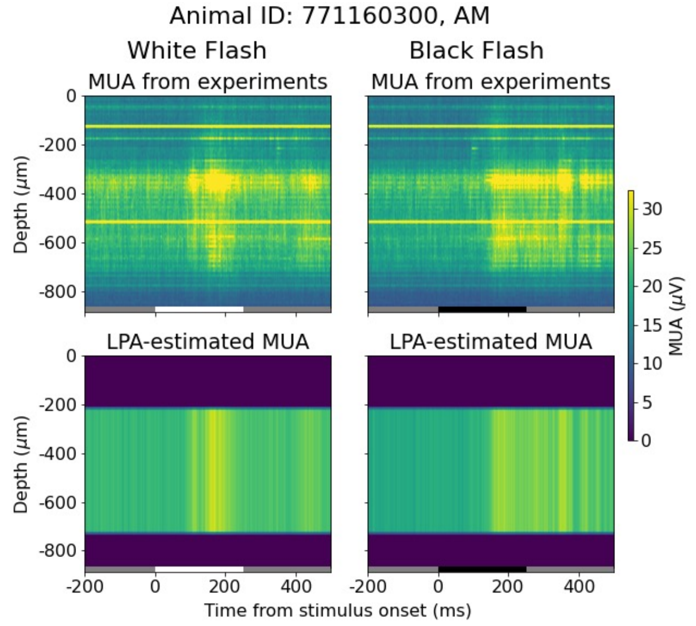

Session ID: 771999200

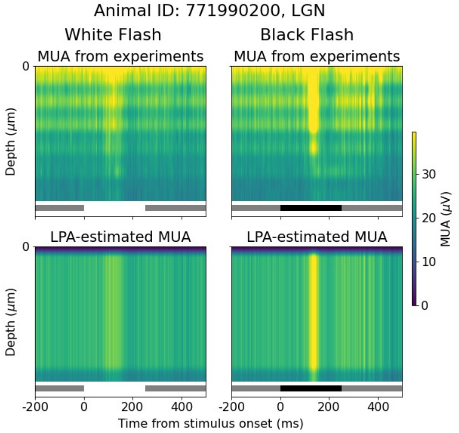

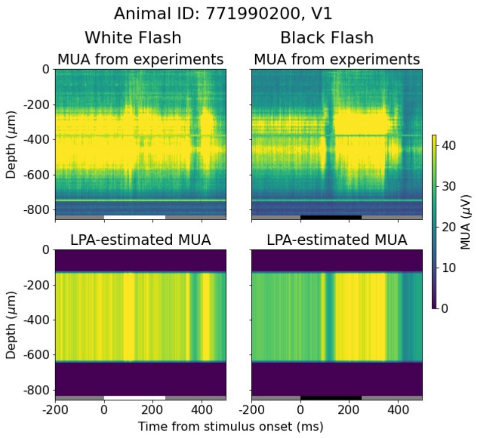

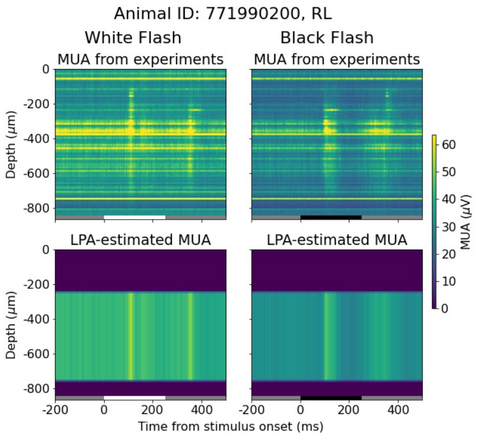

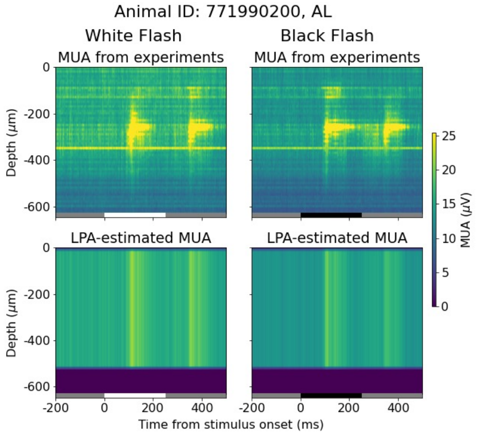

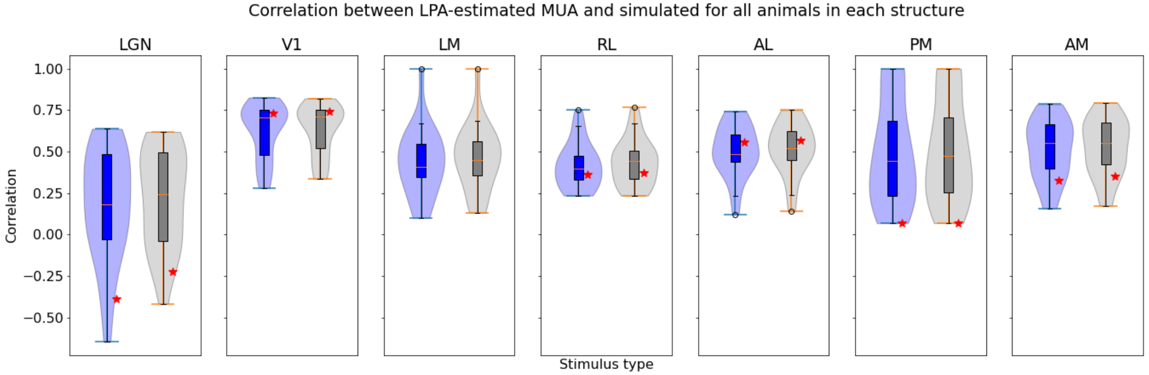

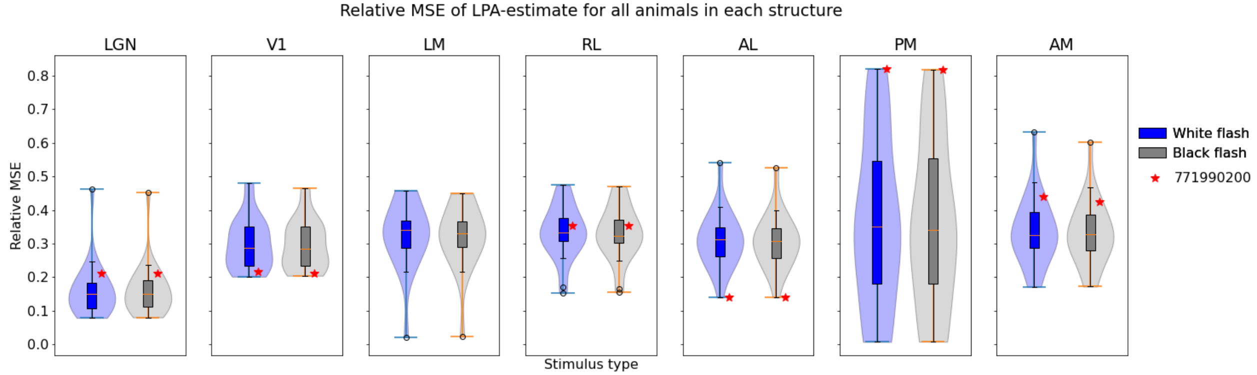

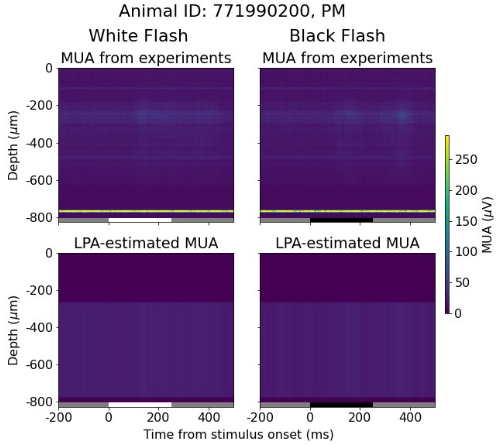

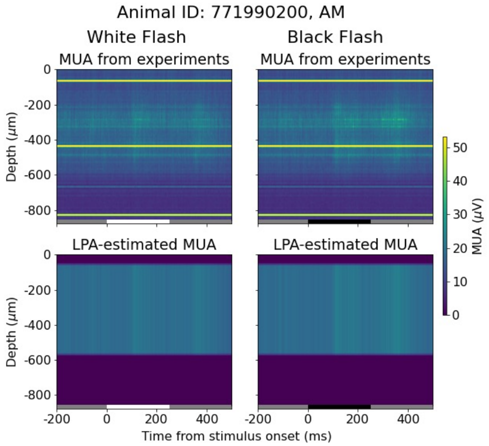

Session ID: 778240327

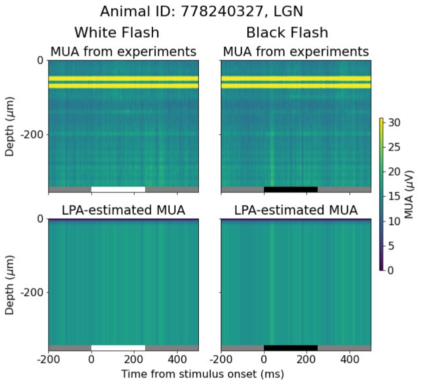

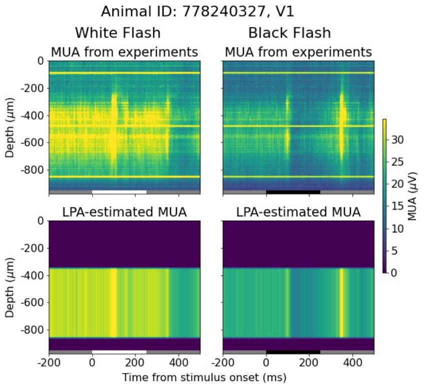

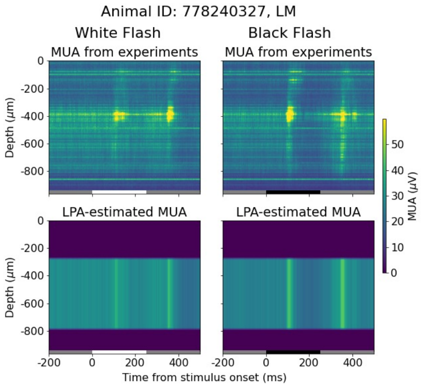

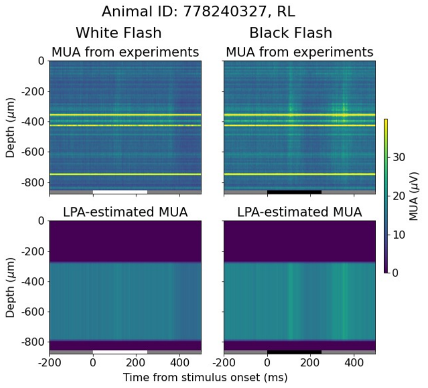

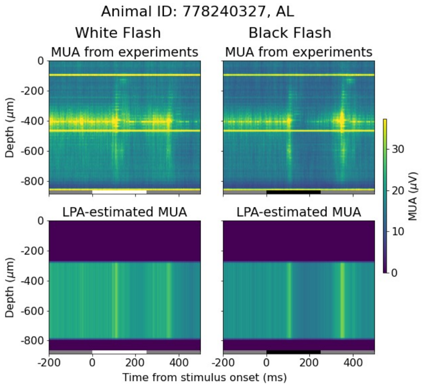

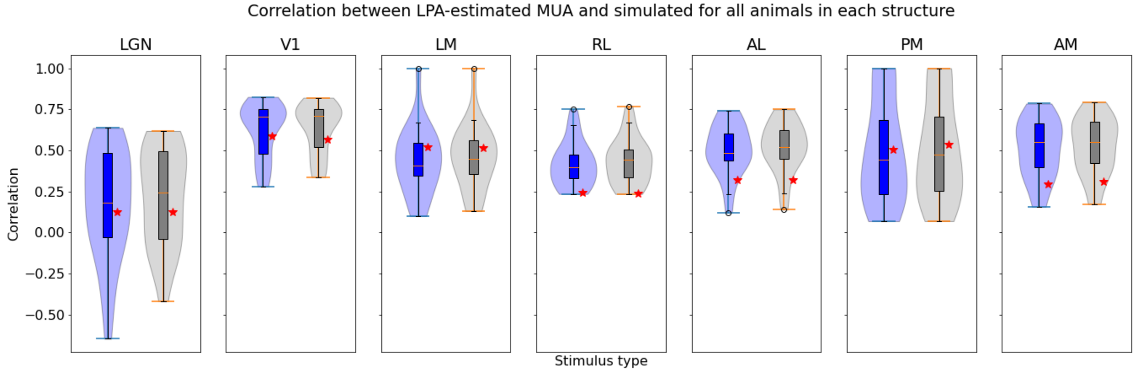

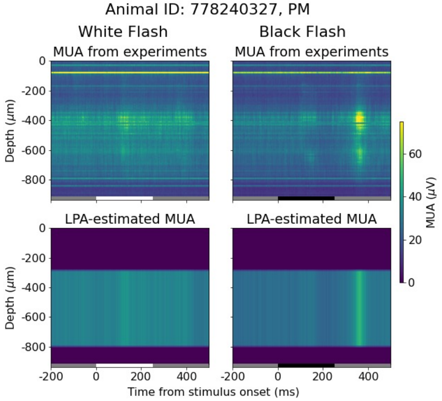

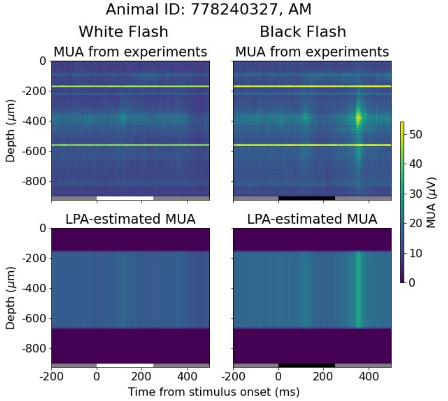

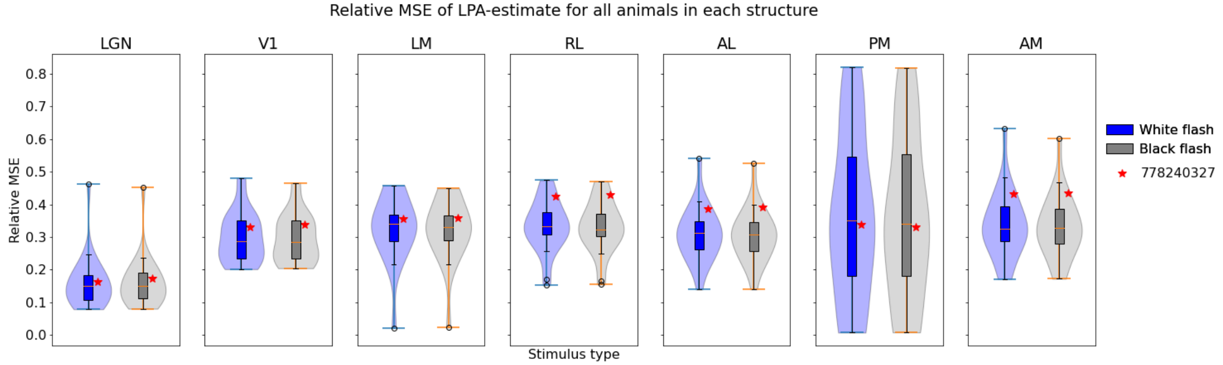

Session ID: 779839471

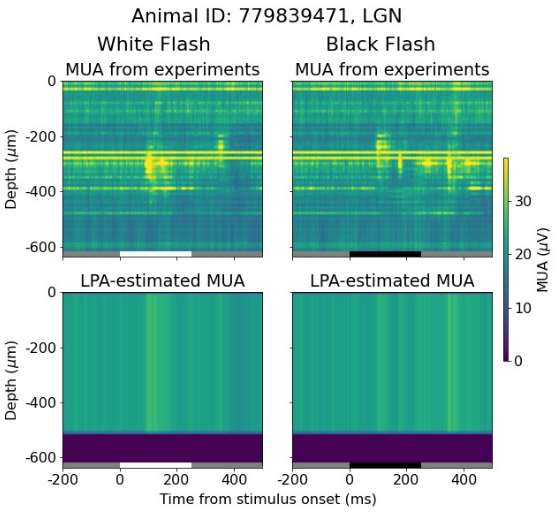

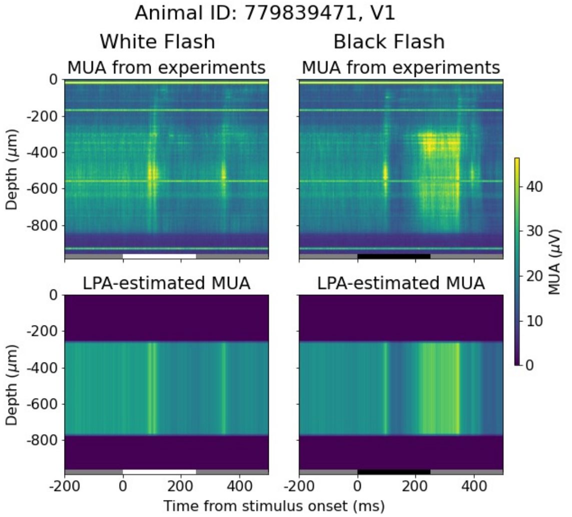

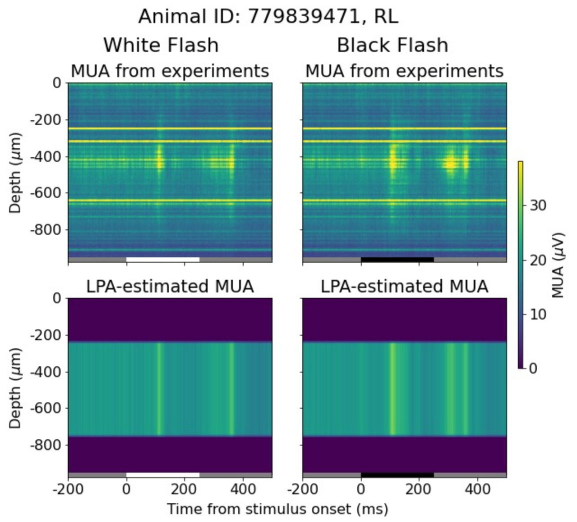

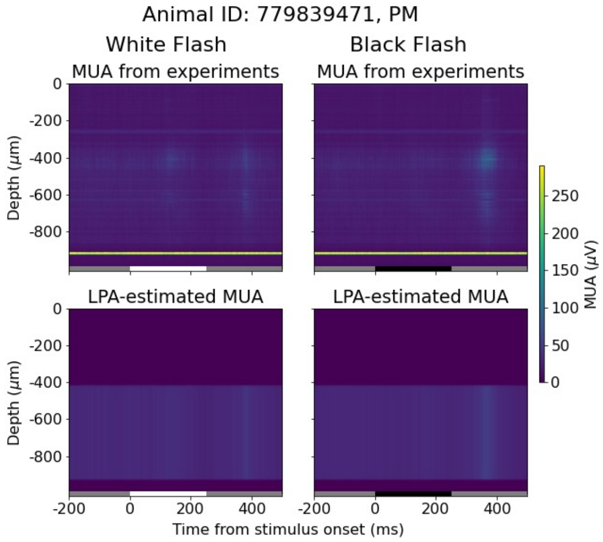

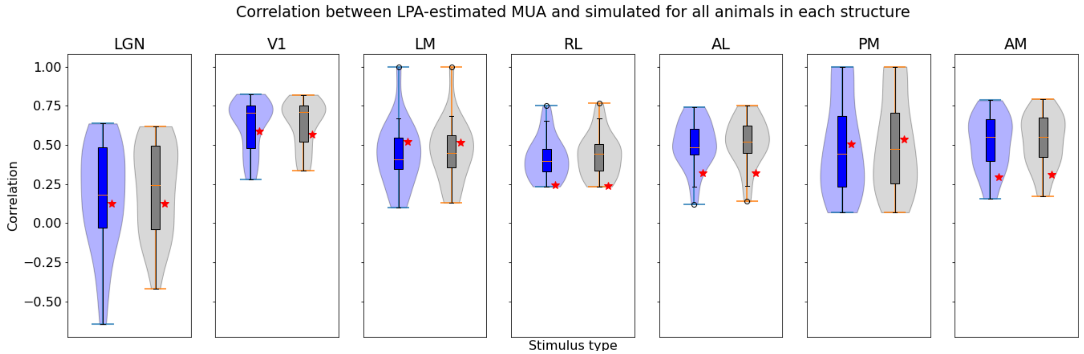

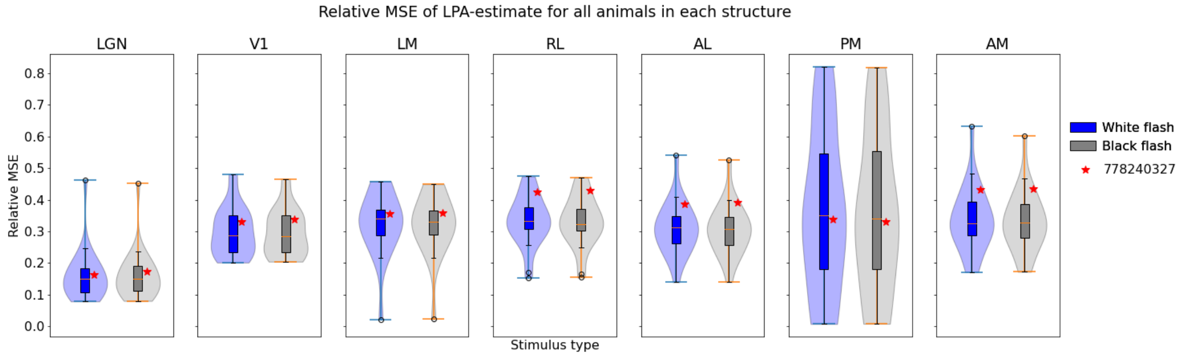

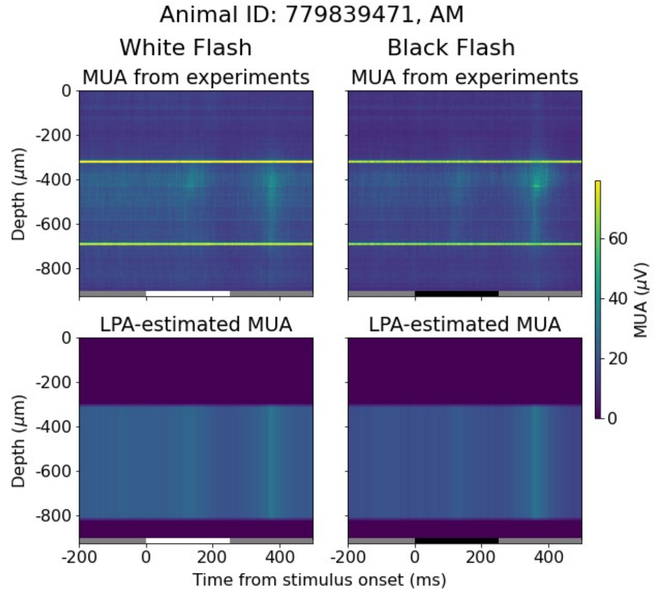

Session ID: 781842082

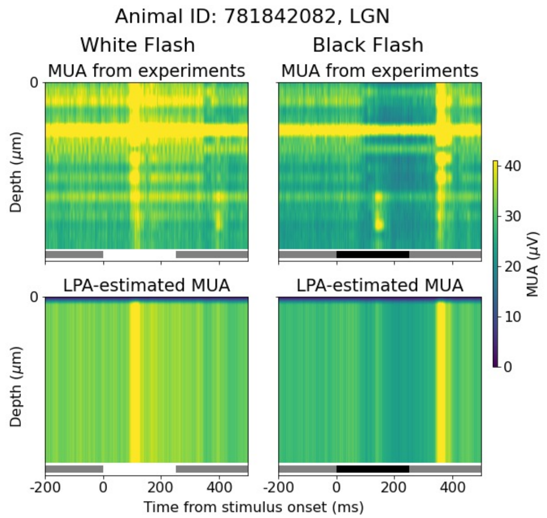

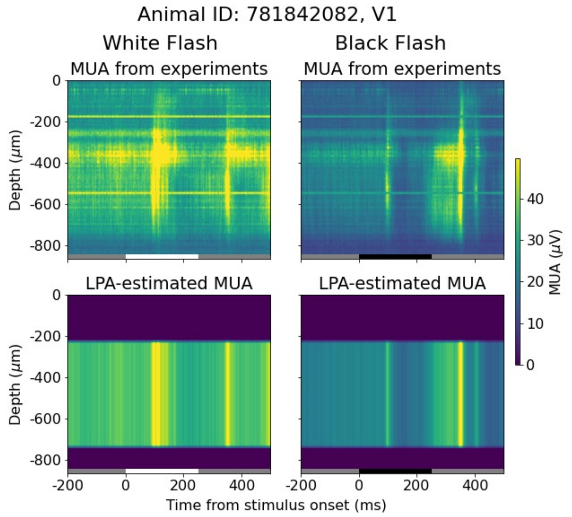

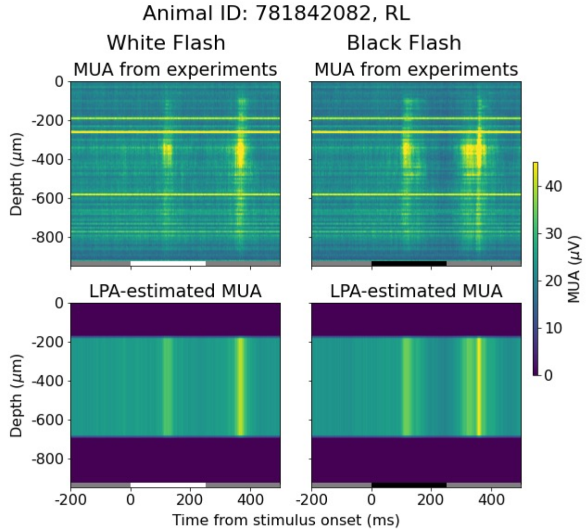

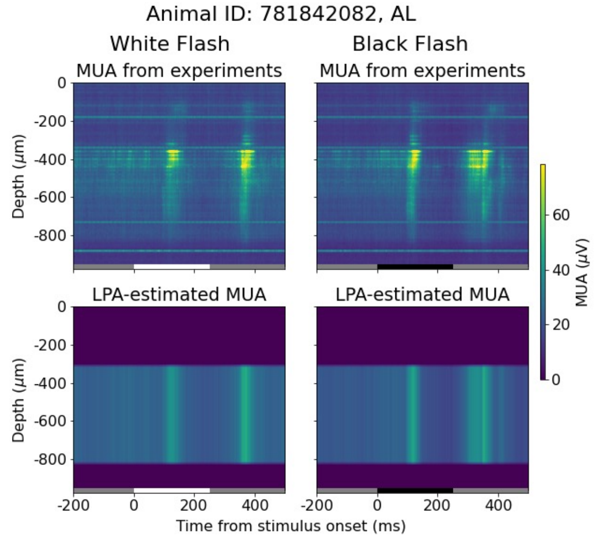

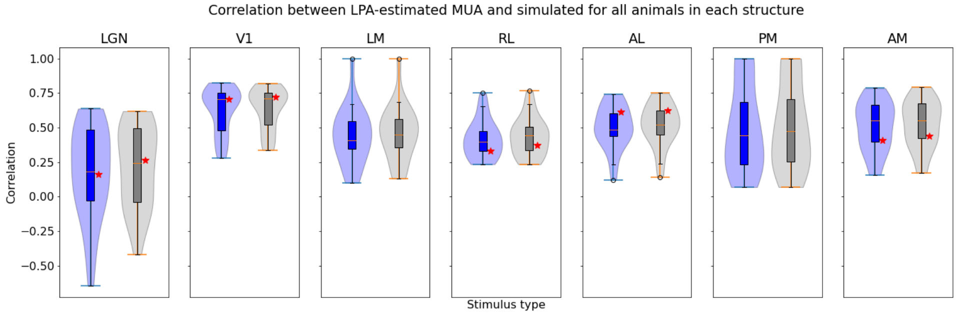

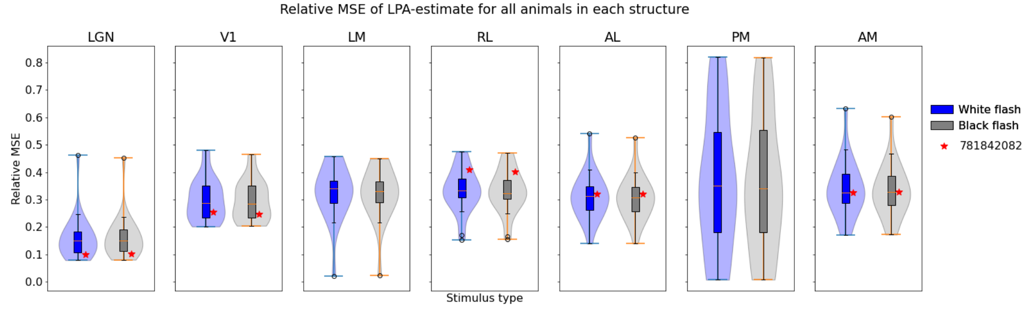

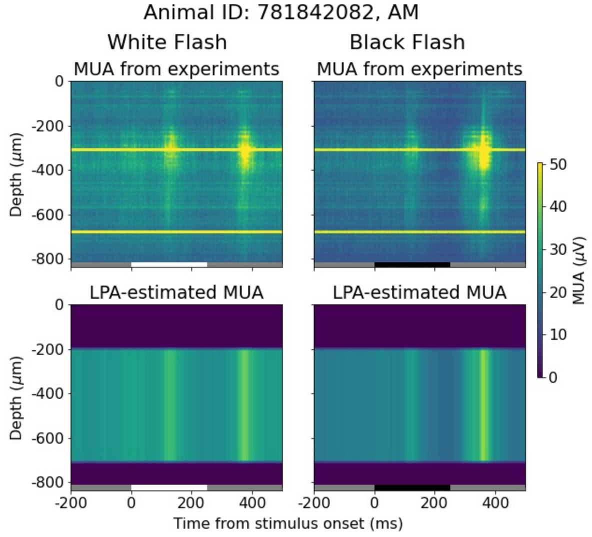

Session ID: 791319847

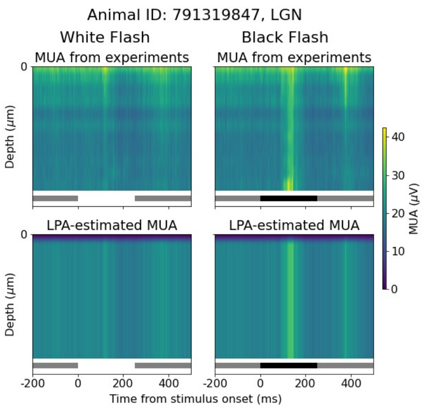

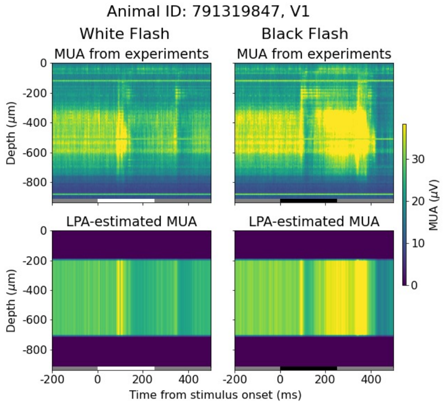

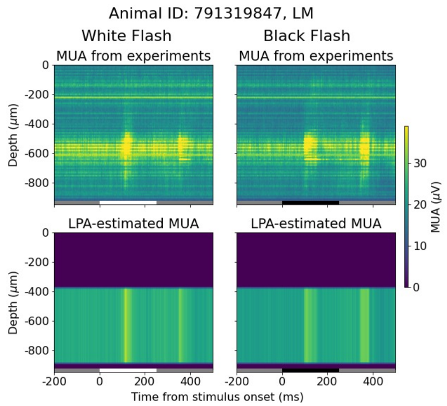

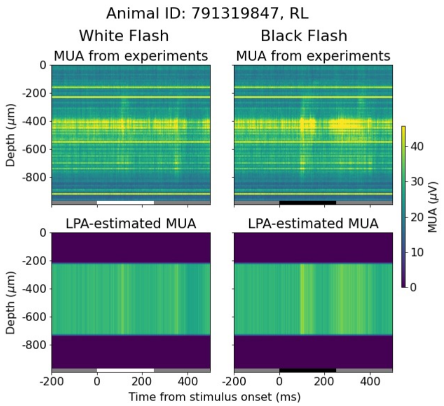

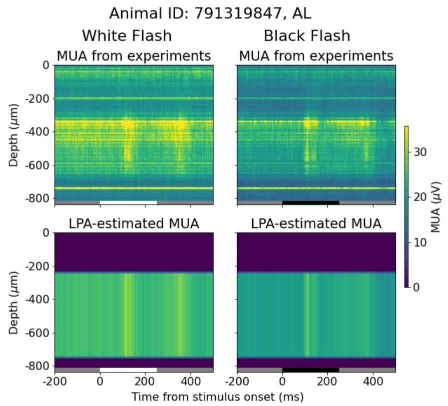

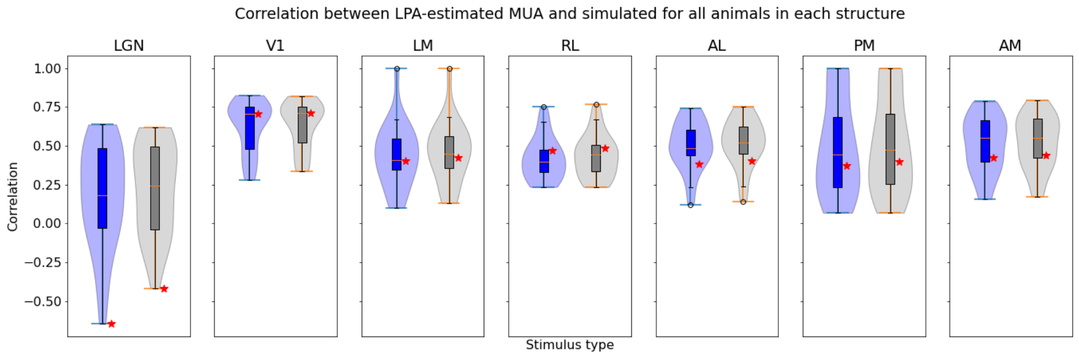

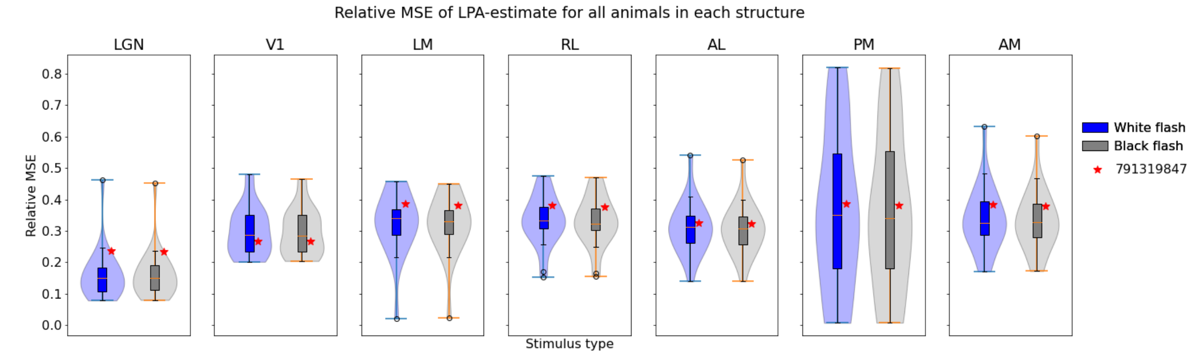

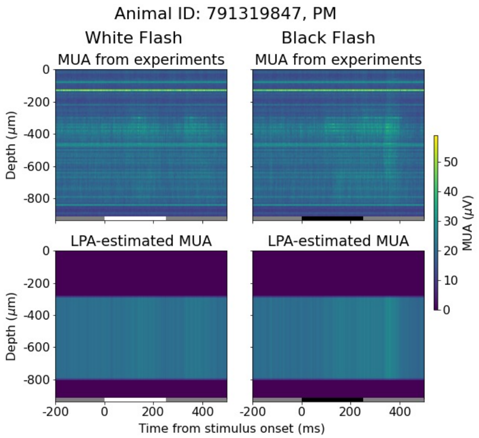

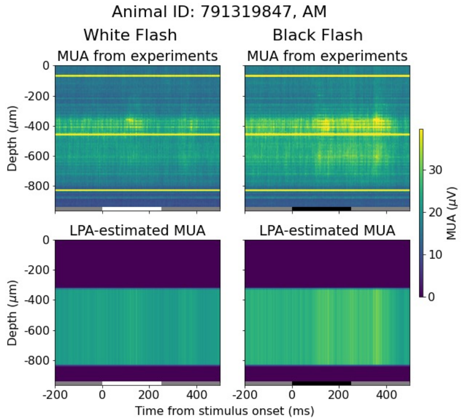

Session ID: 799864342

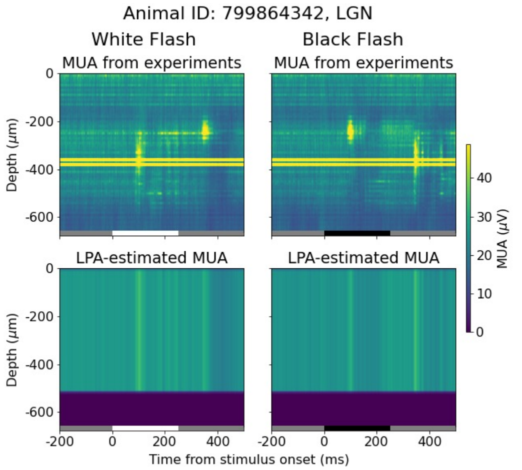

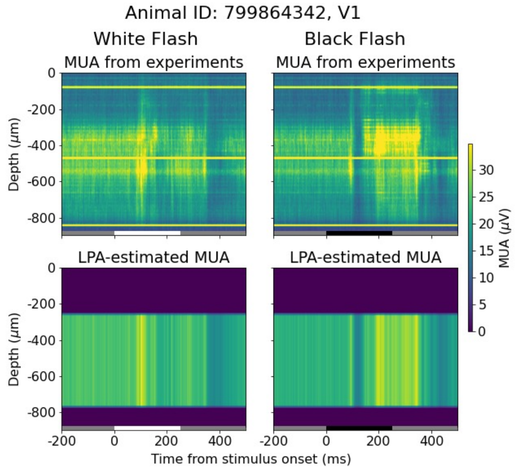

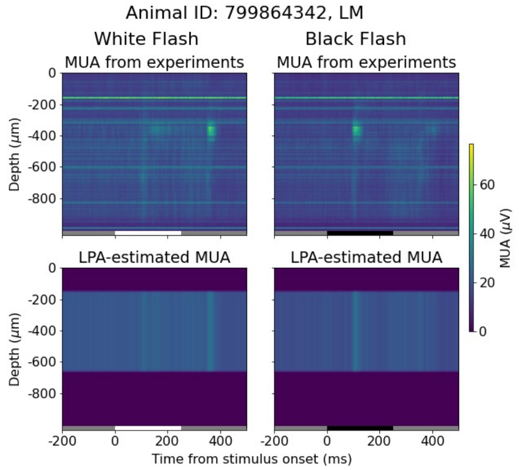

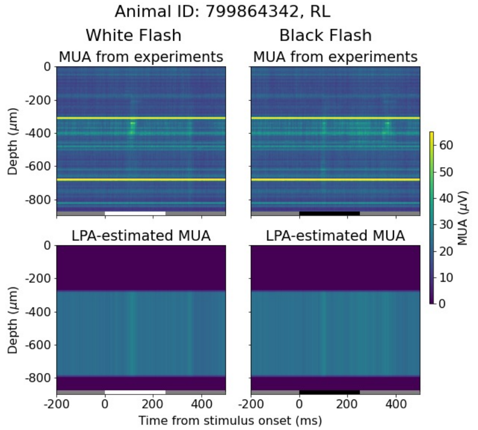

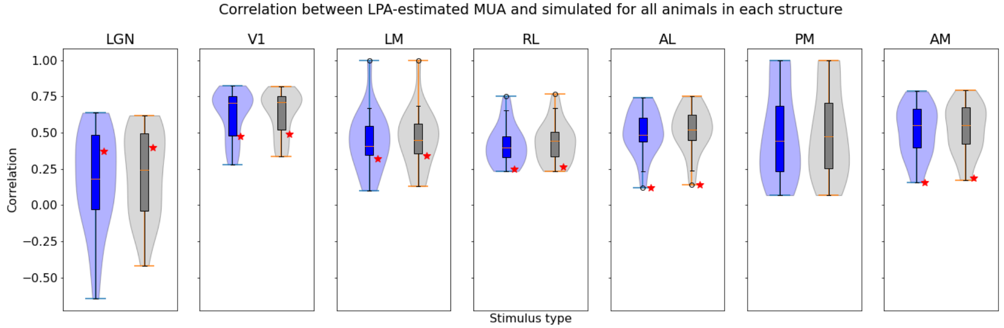

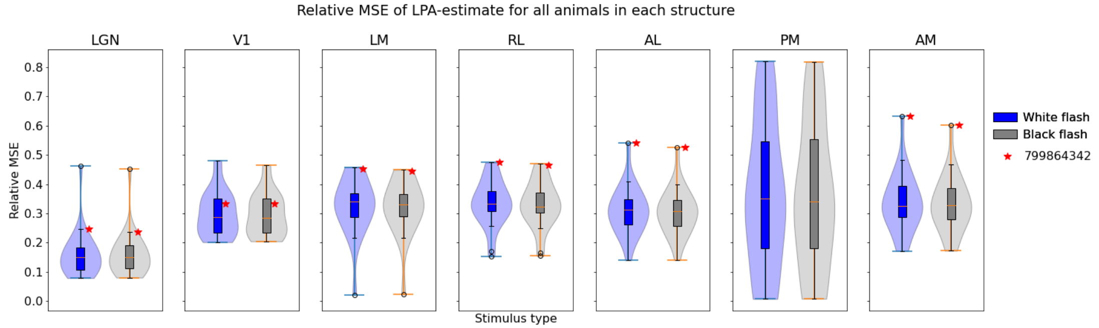

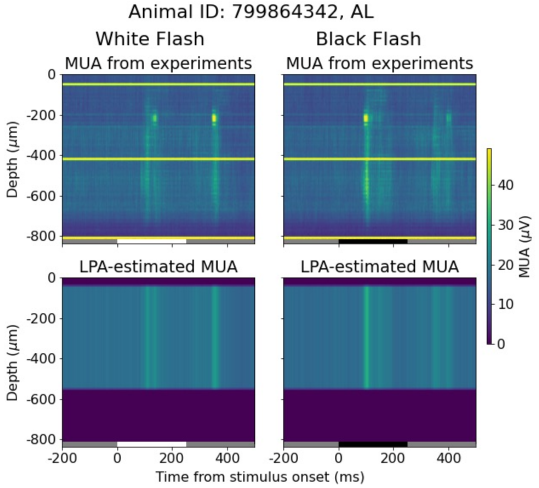

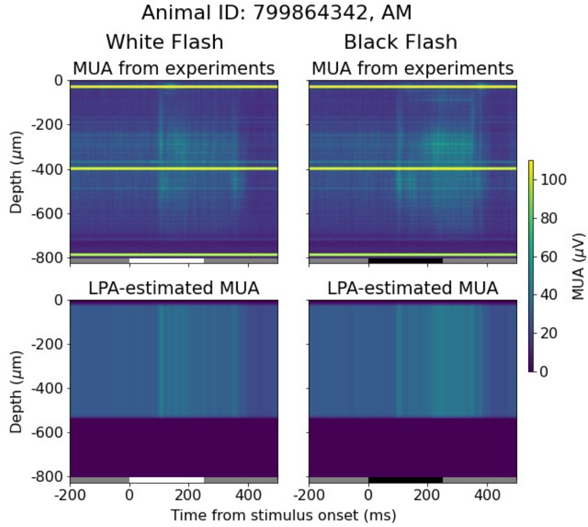

Session ID: 835479236

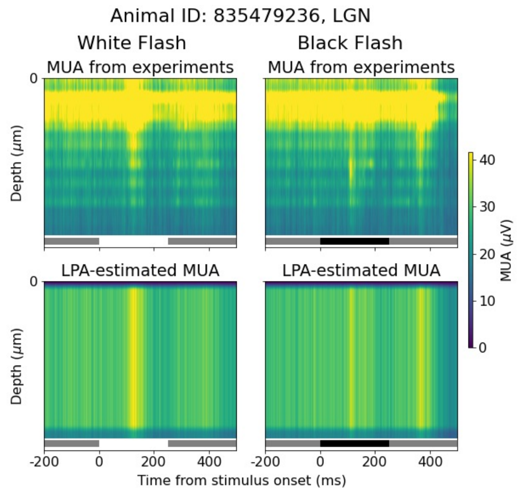

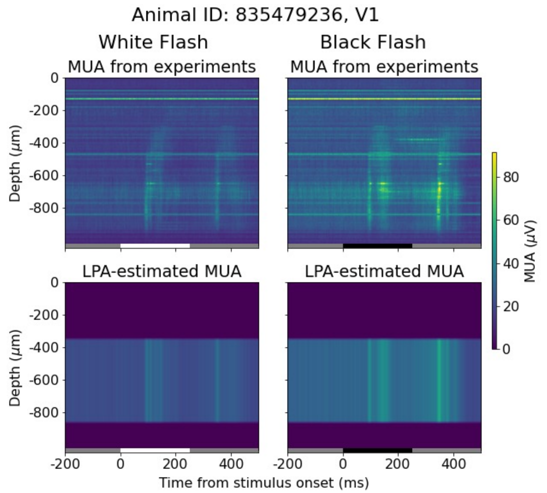

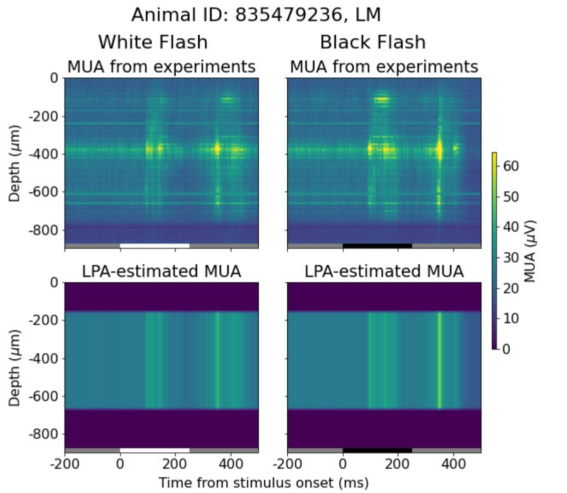

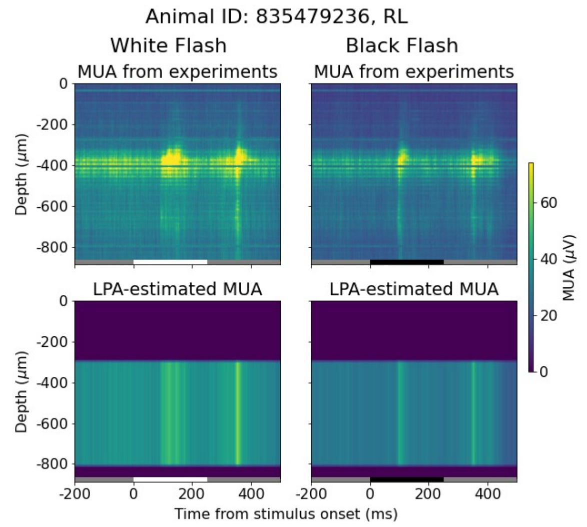

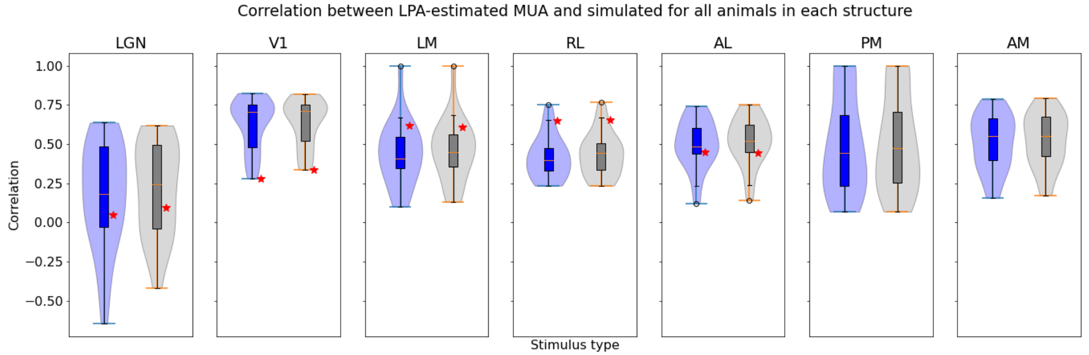

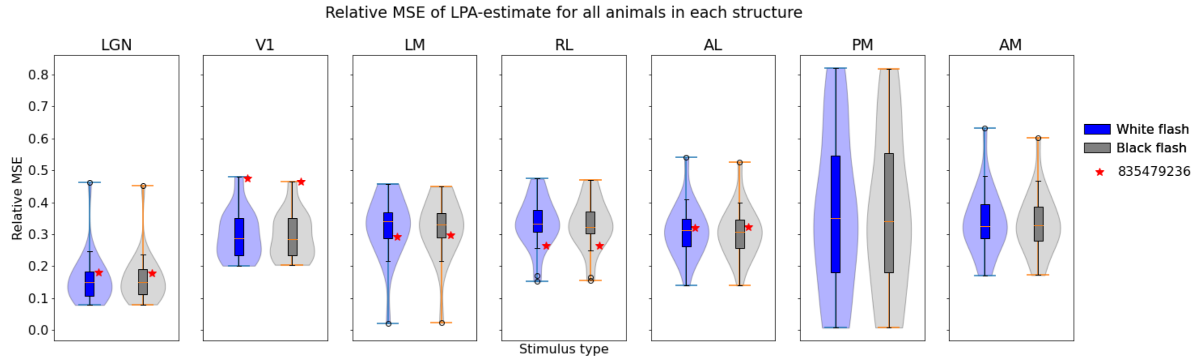

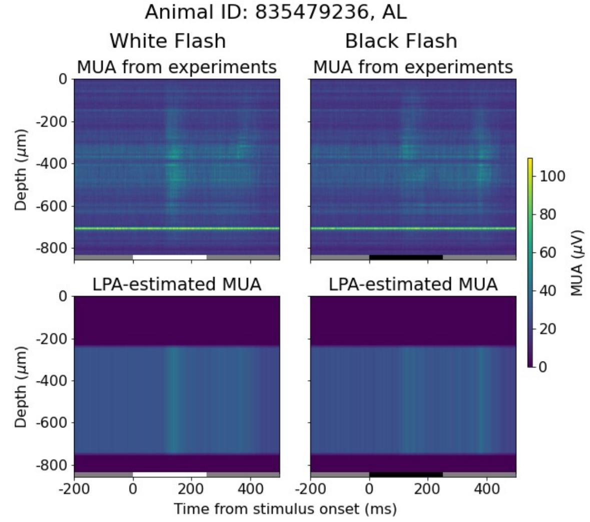

Session ID: 839068429

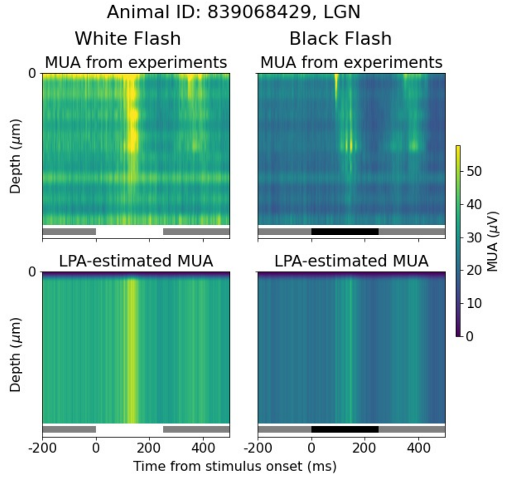

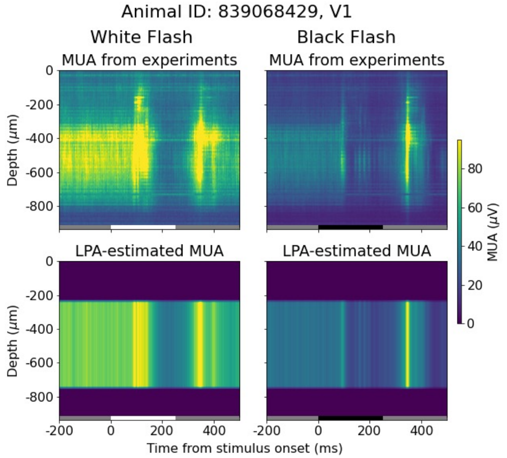

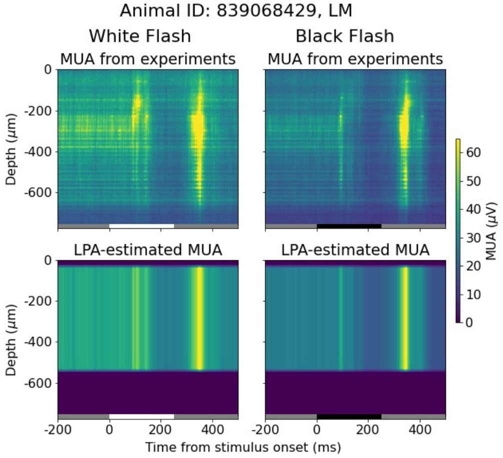

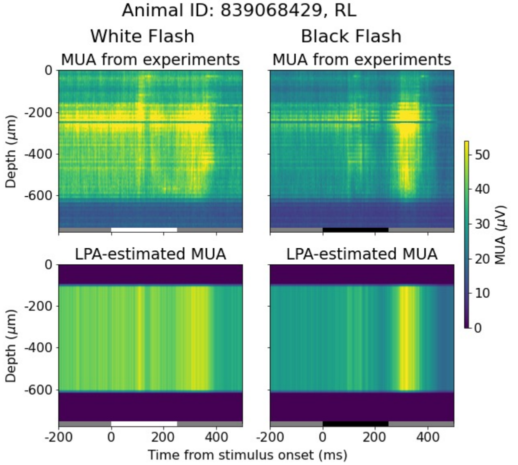

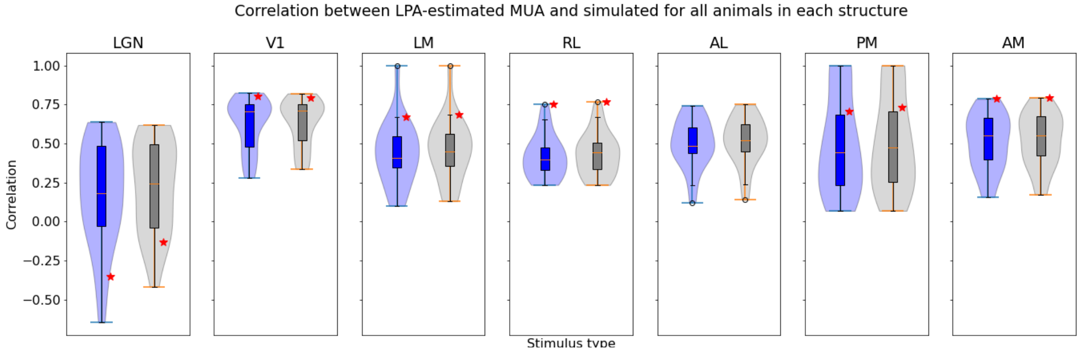

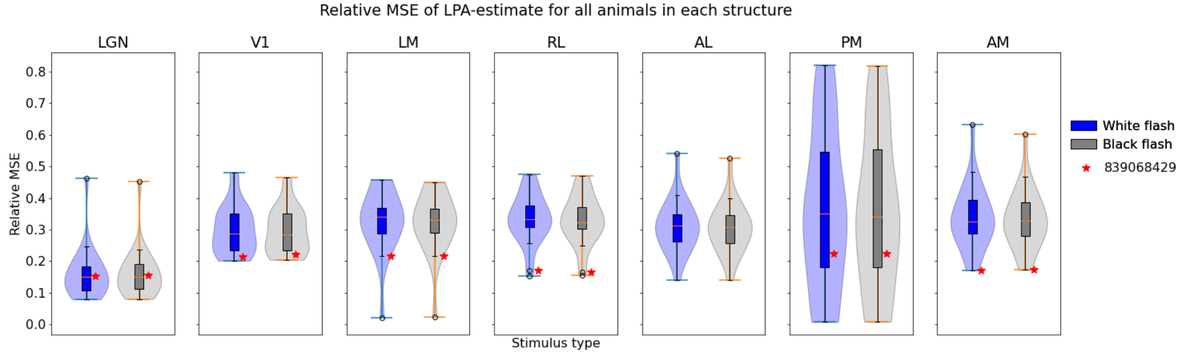

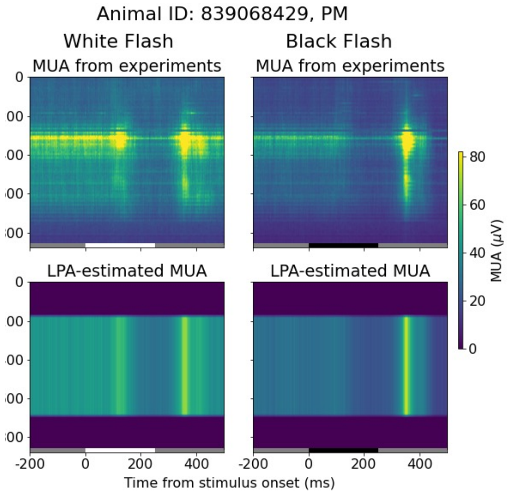

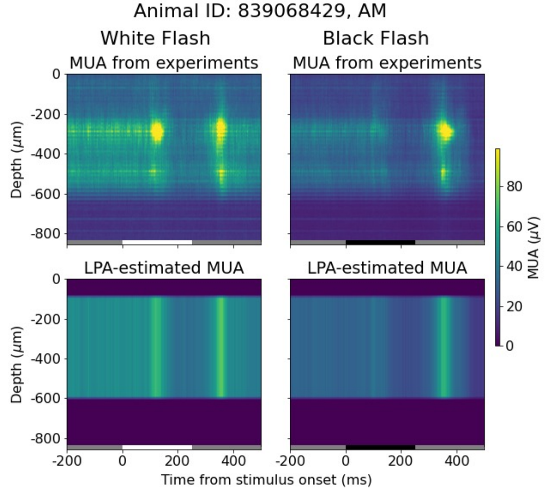

Applying LPA to CSD

Session ID: 71509703

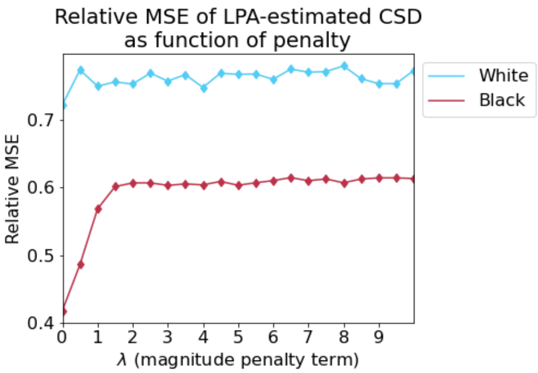

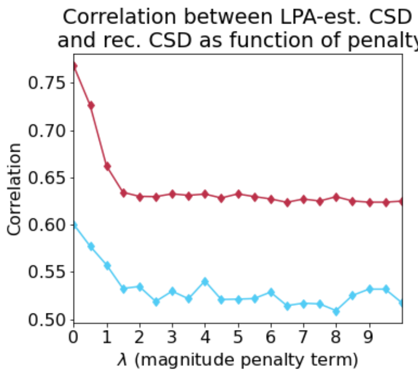

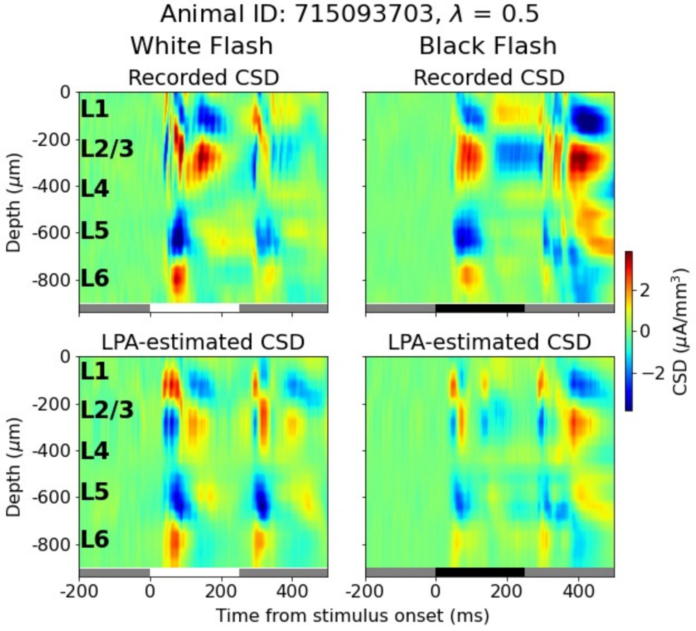

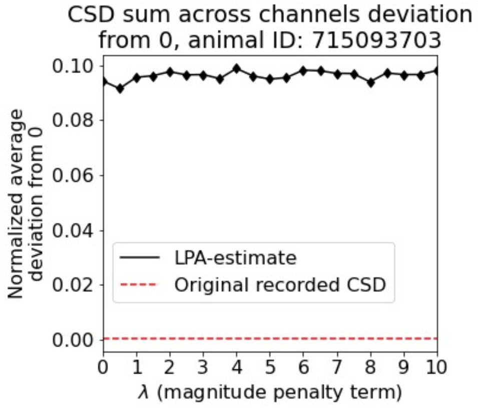

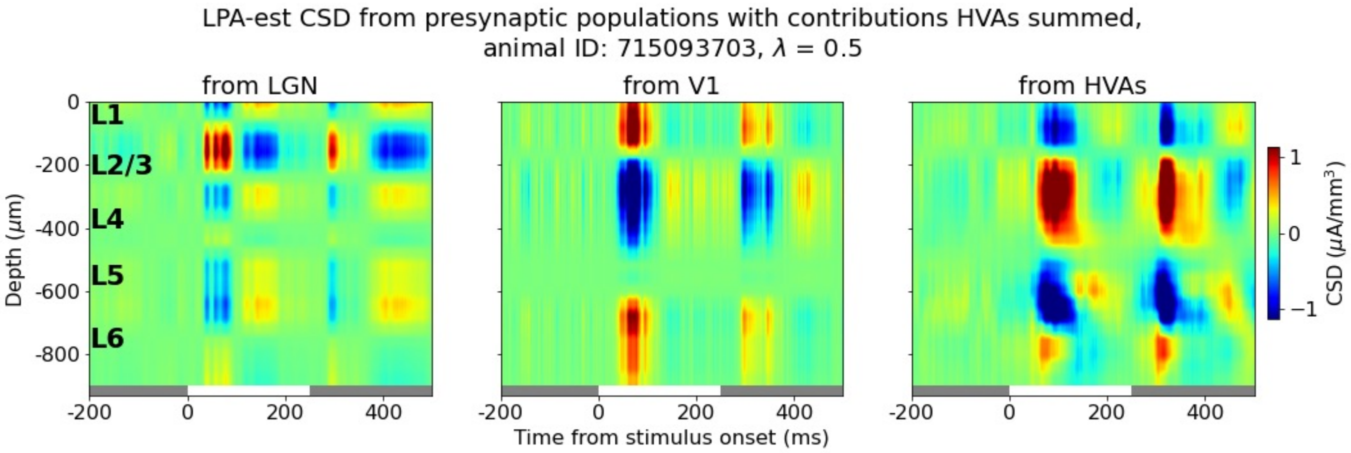

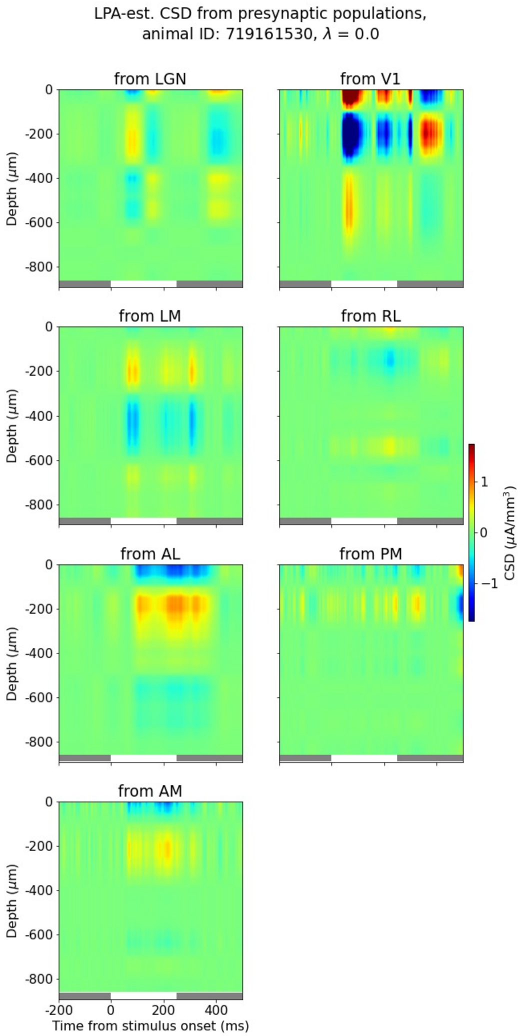

Session ID: 719161530

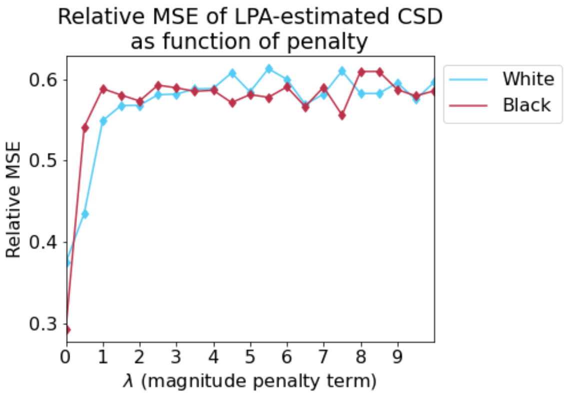

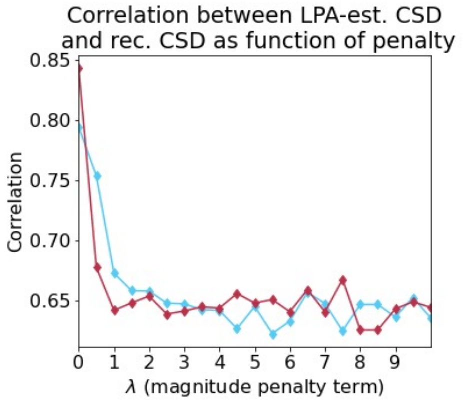

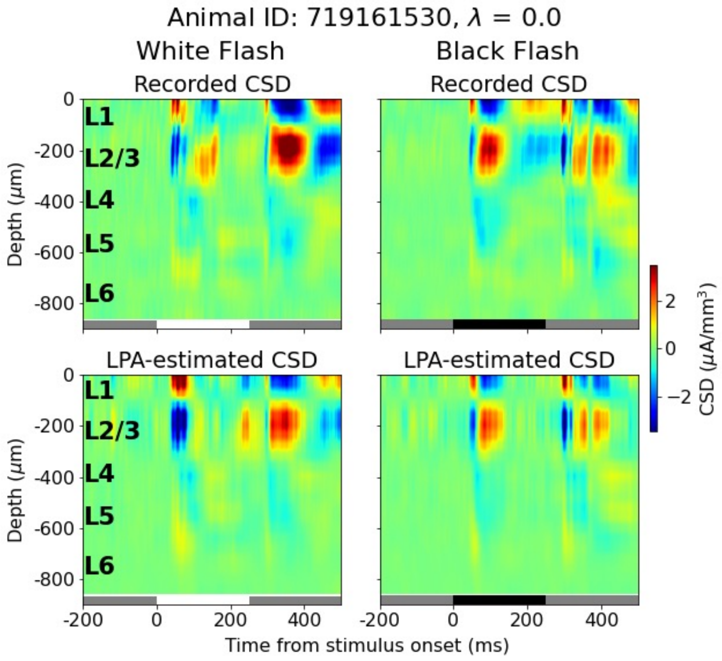

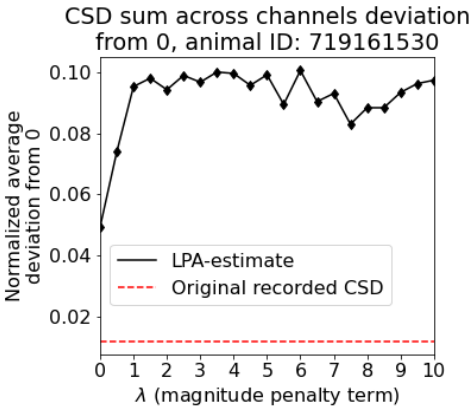

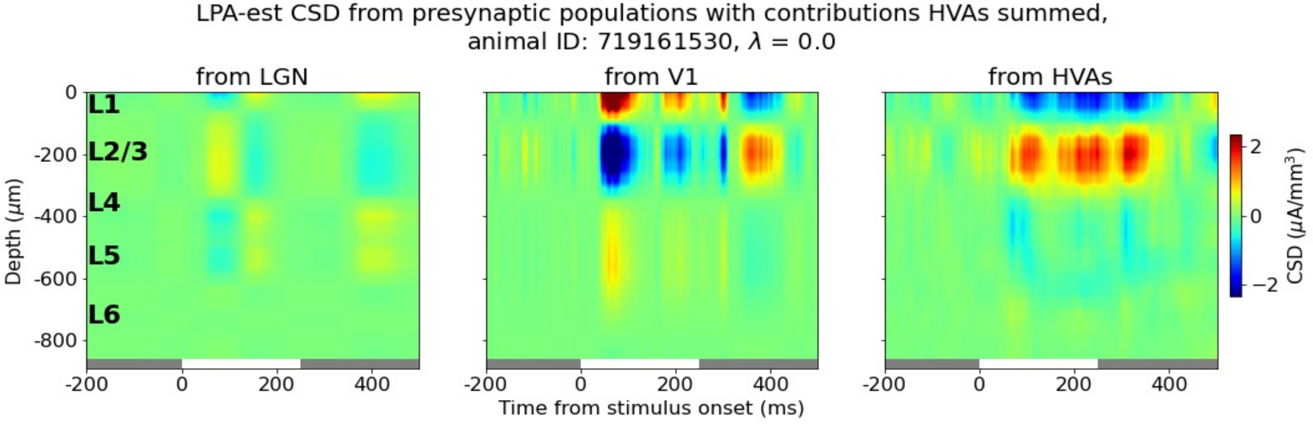

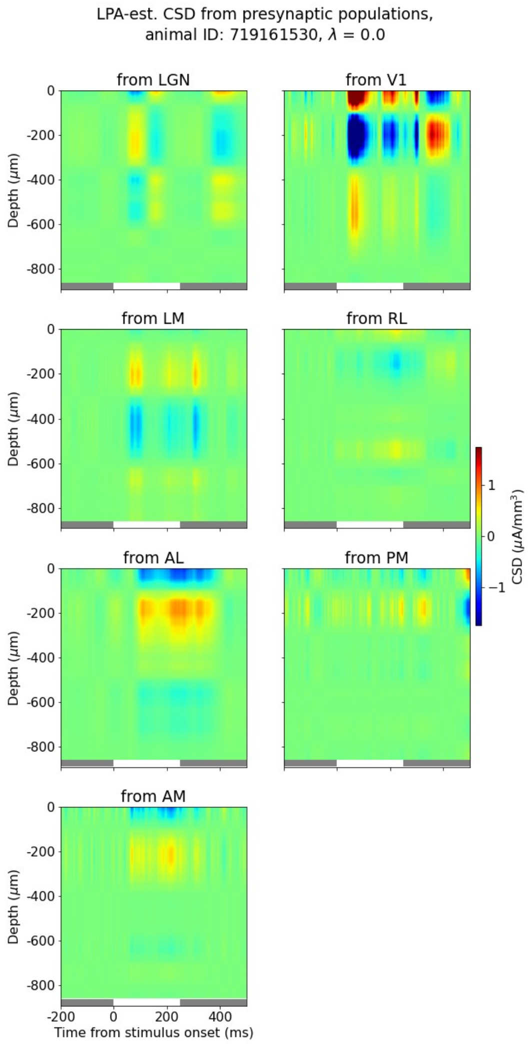

Session ID: 721173822

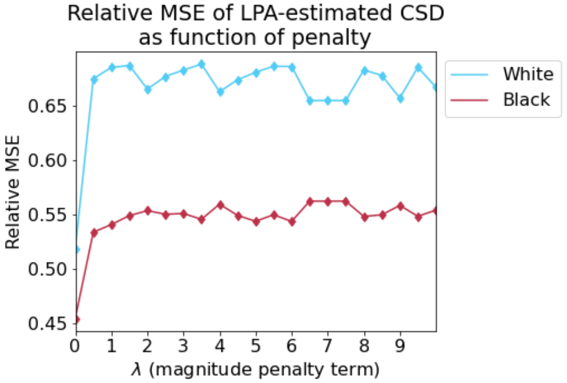

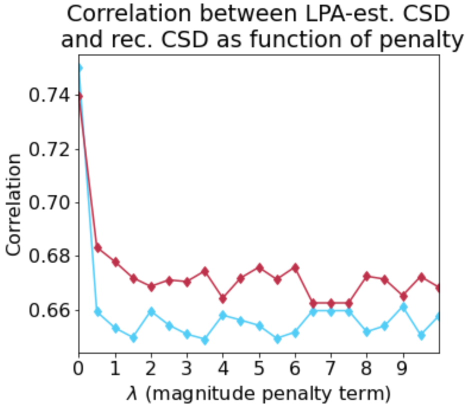

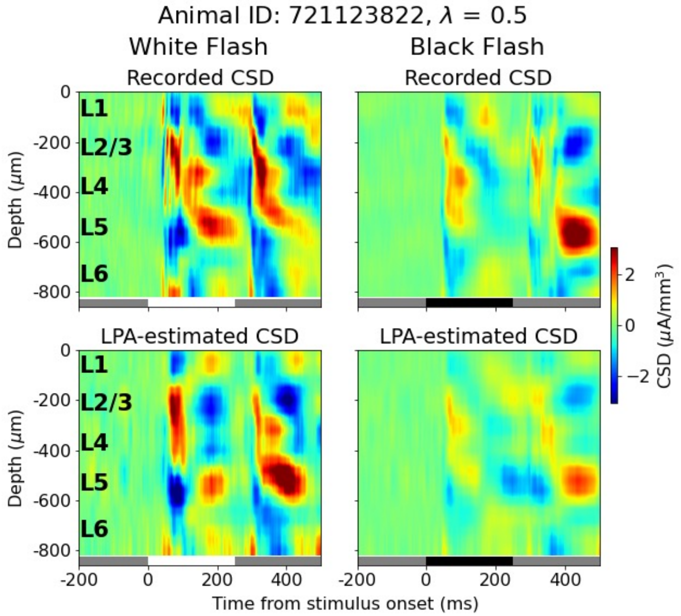

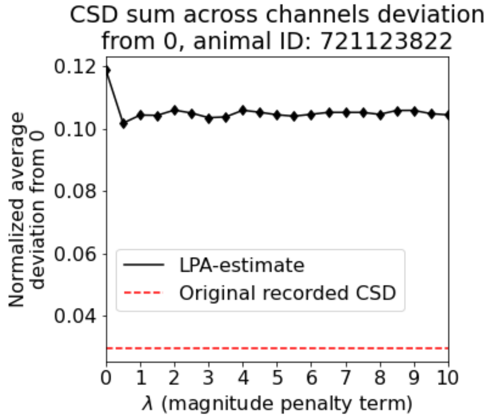

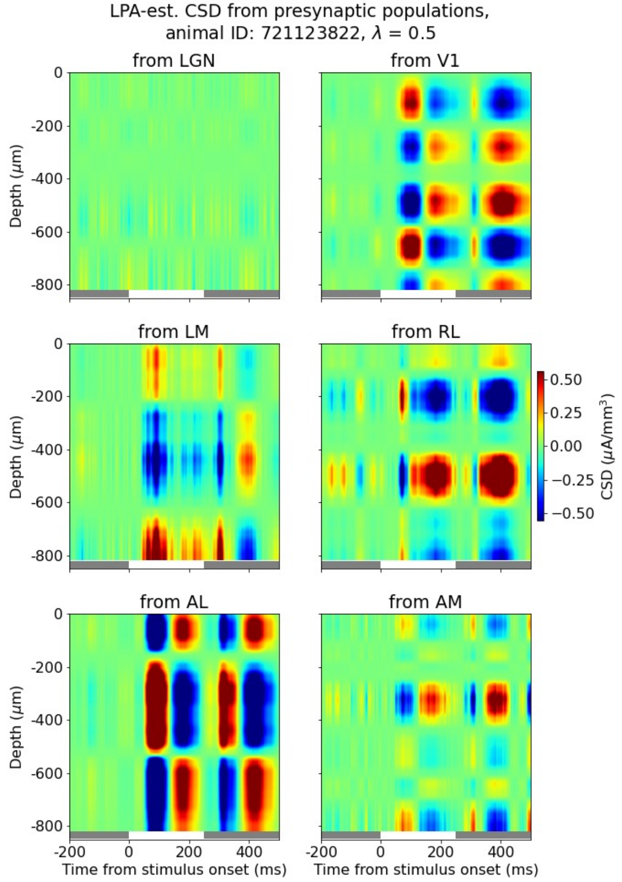

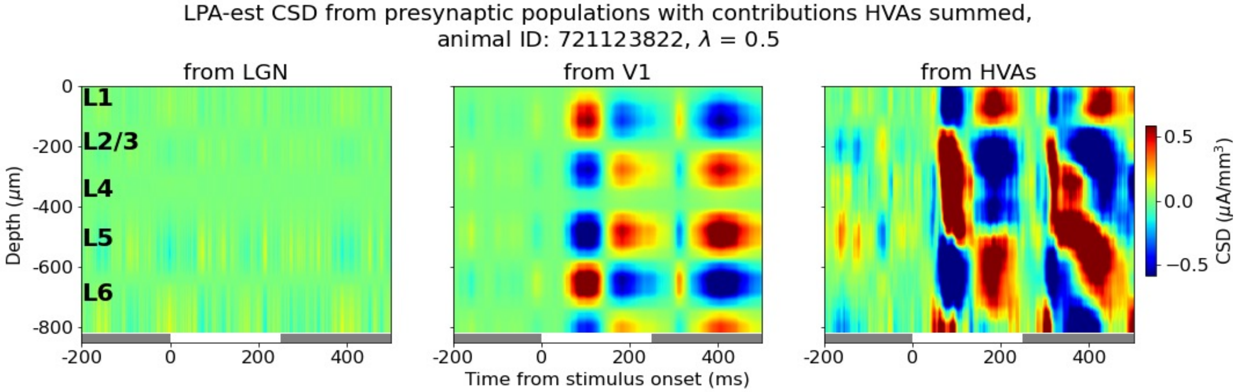

Session ID: 750749662

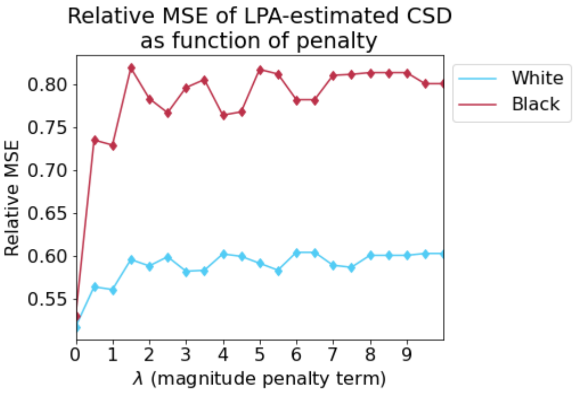

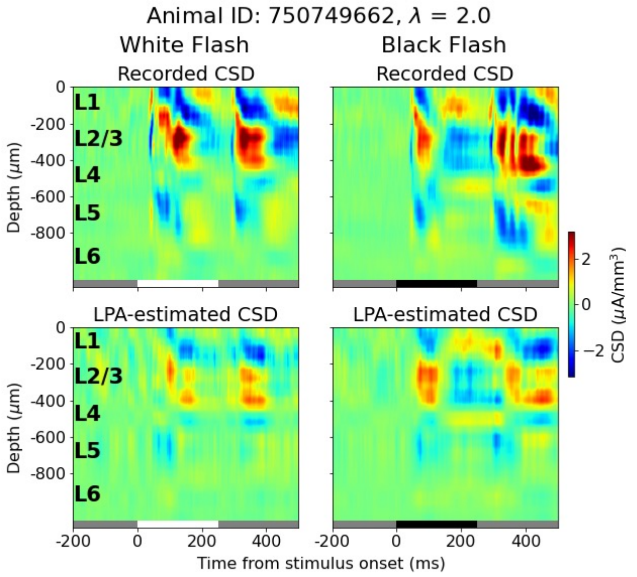

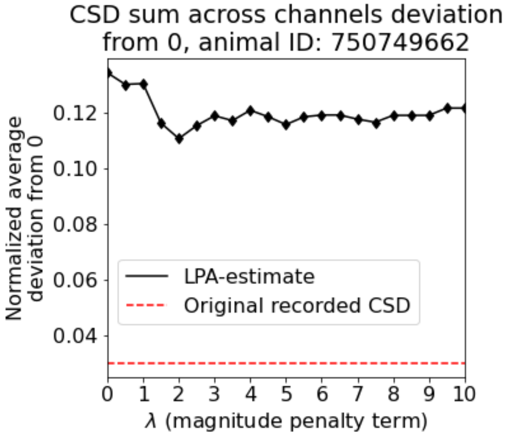

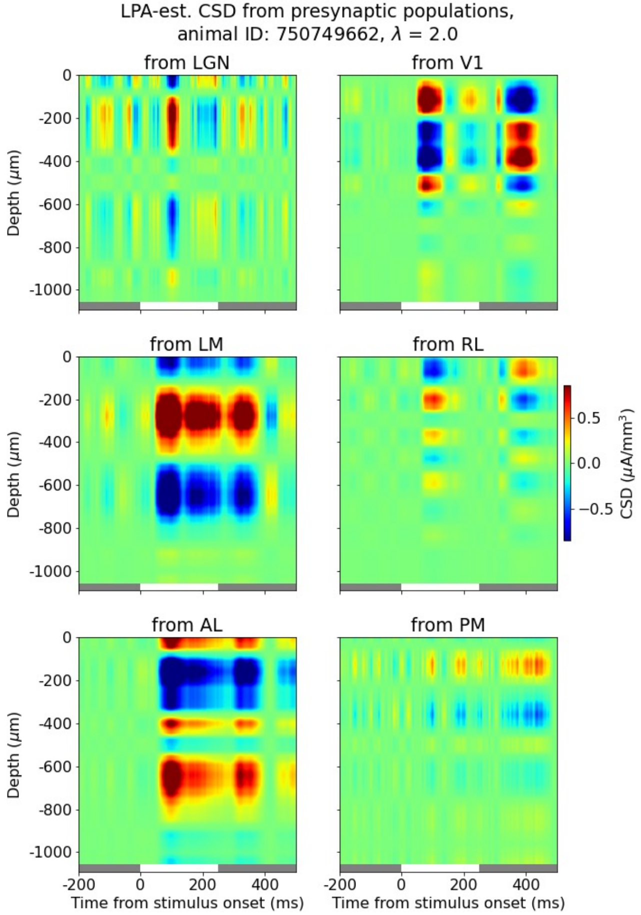

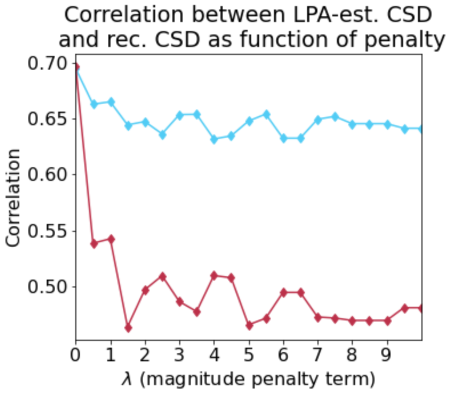

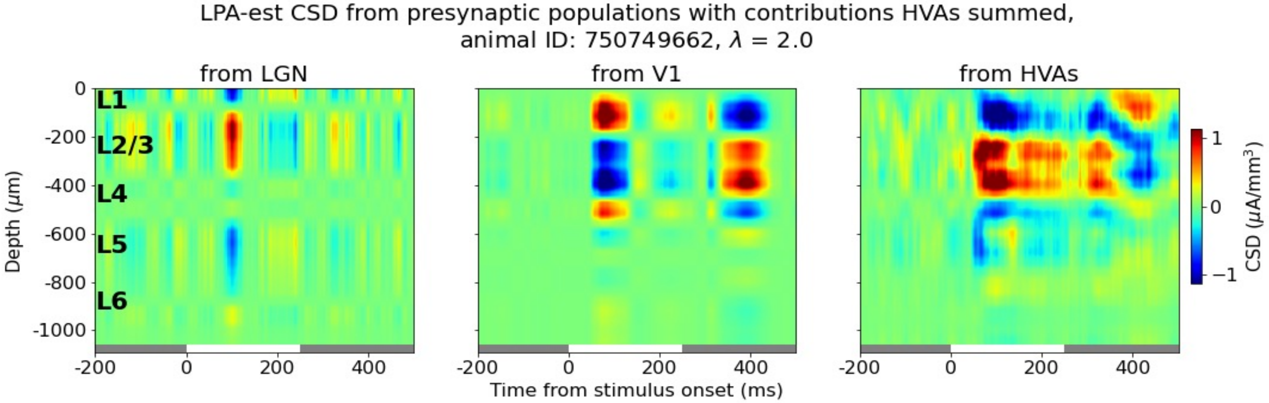

Session ID: 754312389

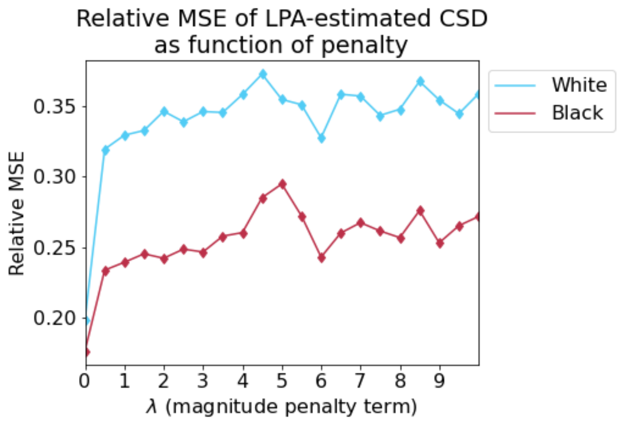

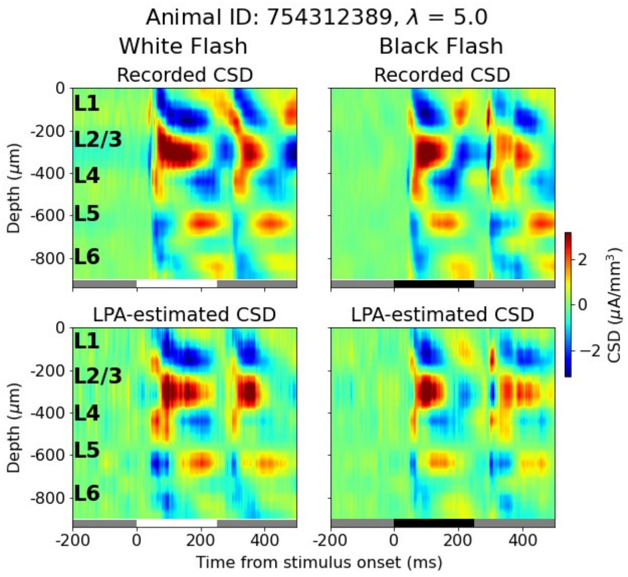

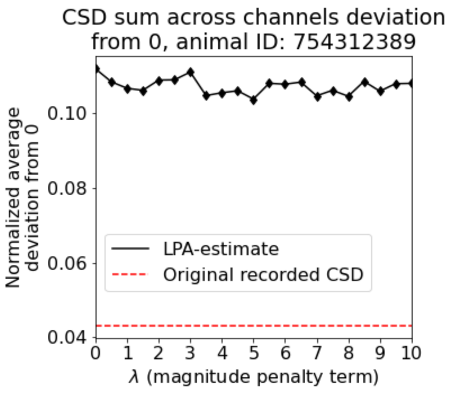

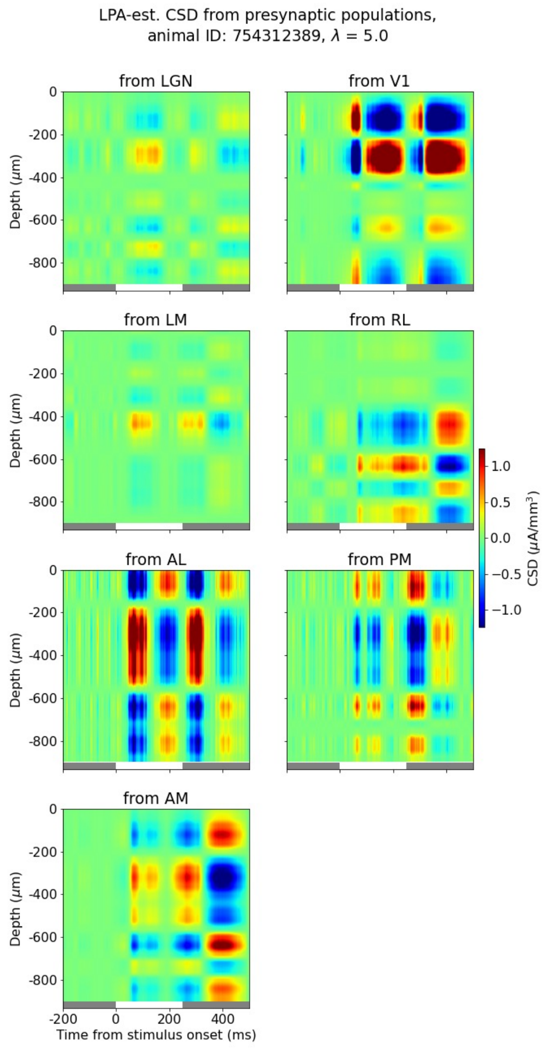

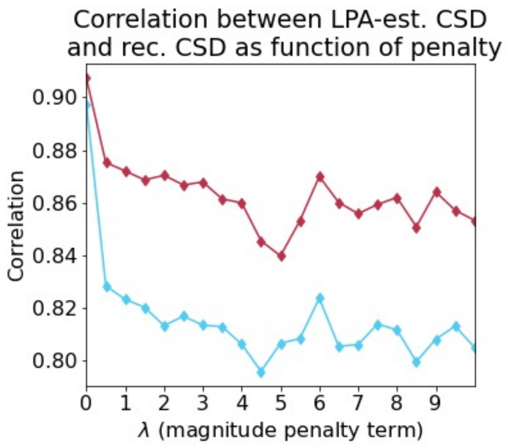

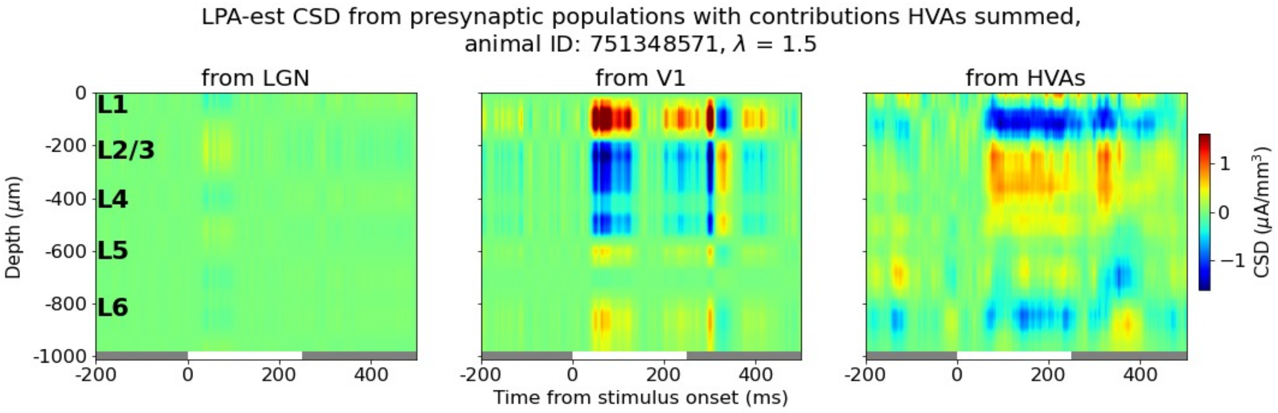

Session ID: 754829445

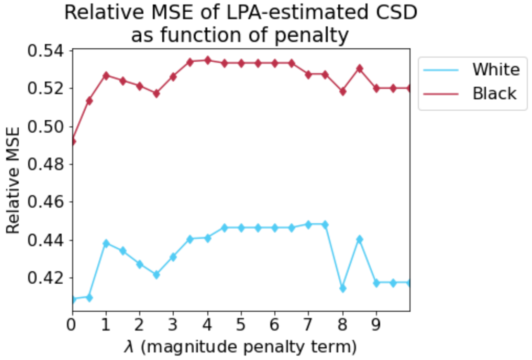

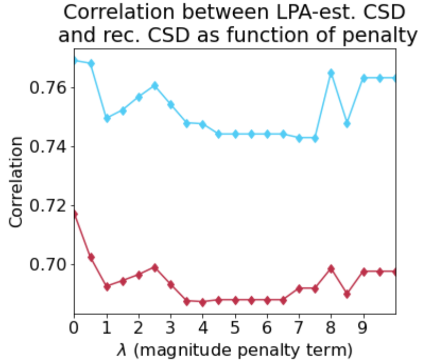

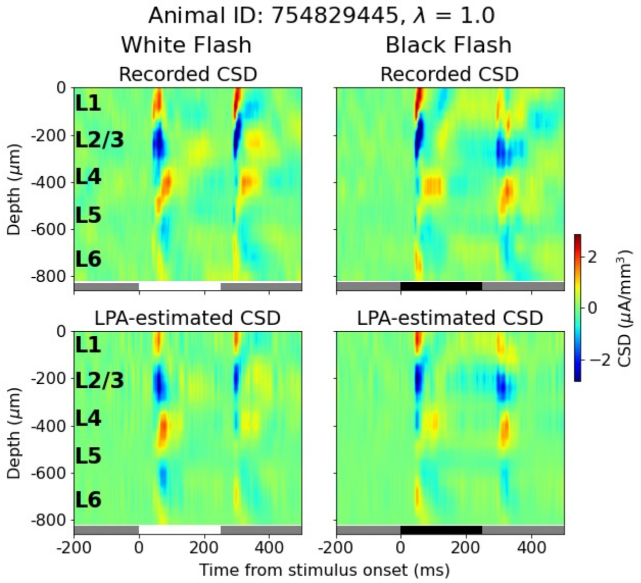

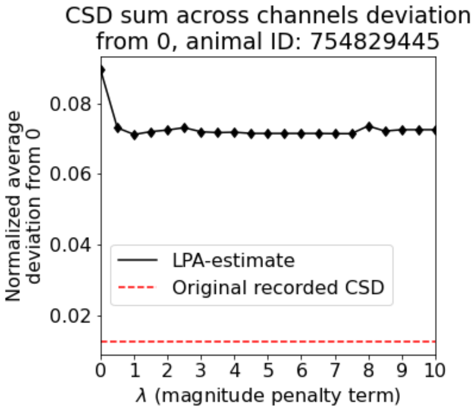

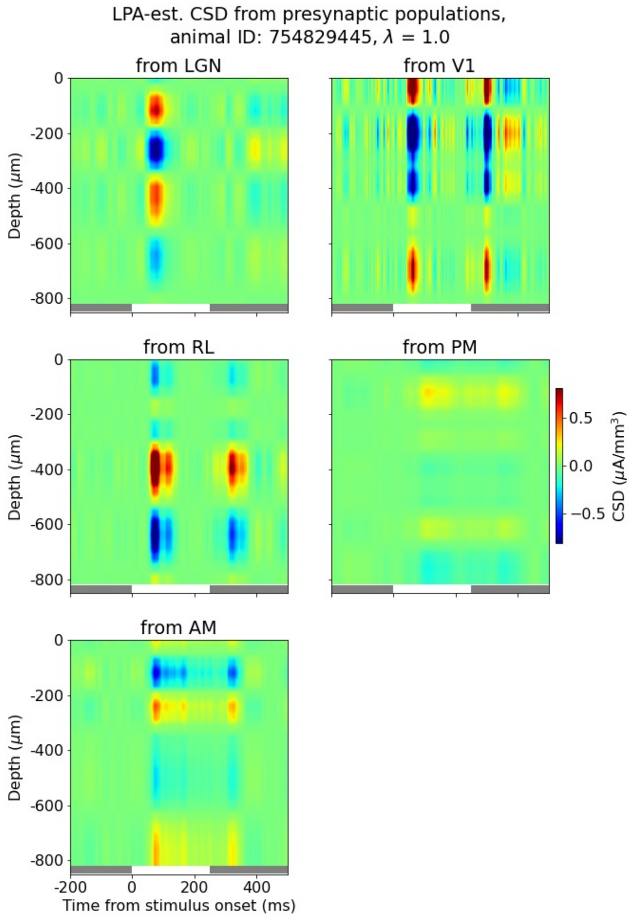

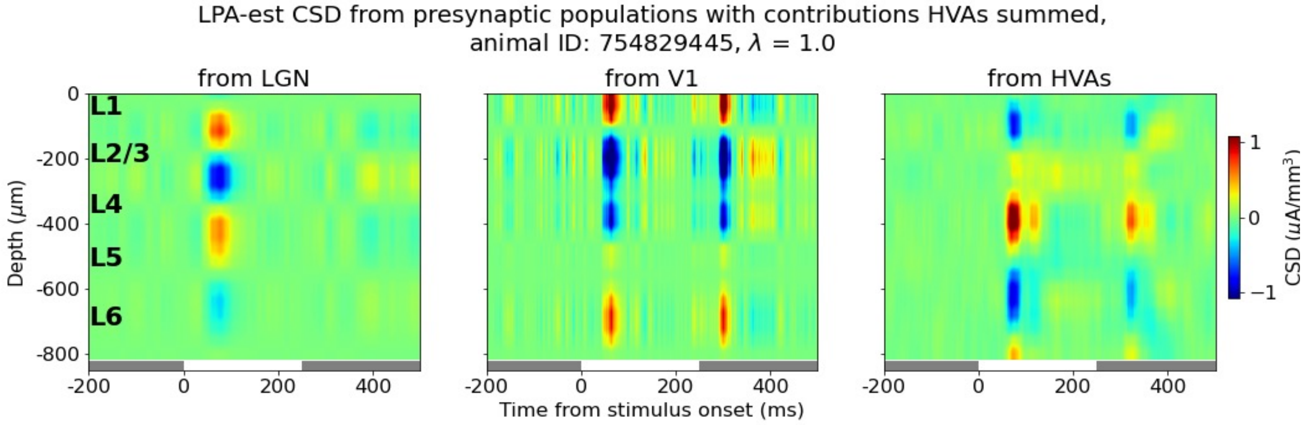

Session ID: 755434585

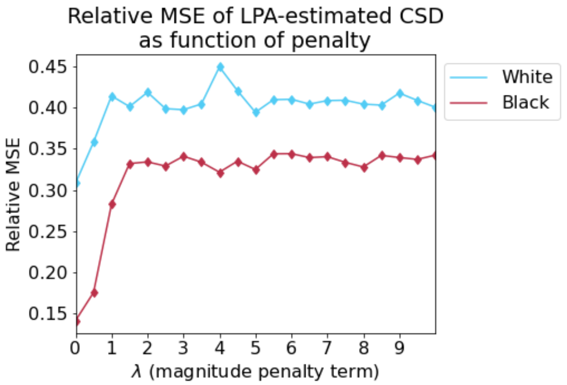

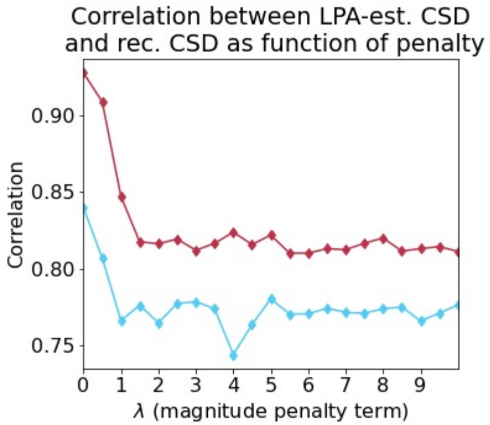

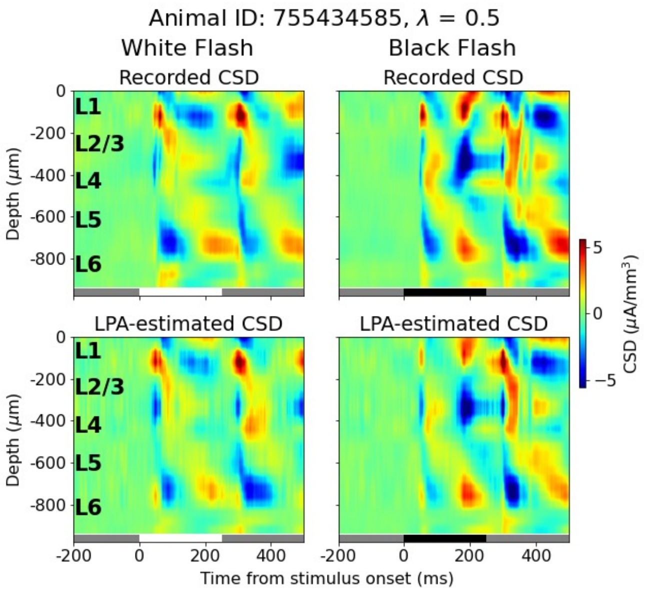

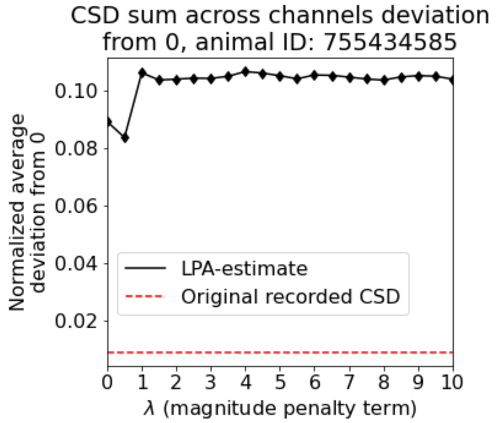

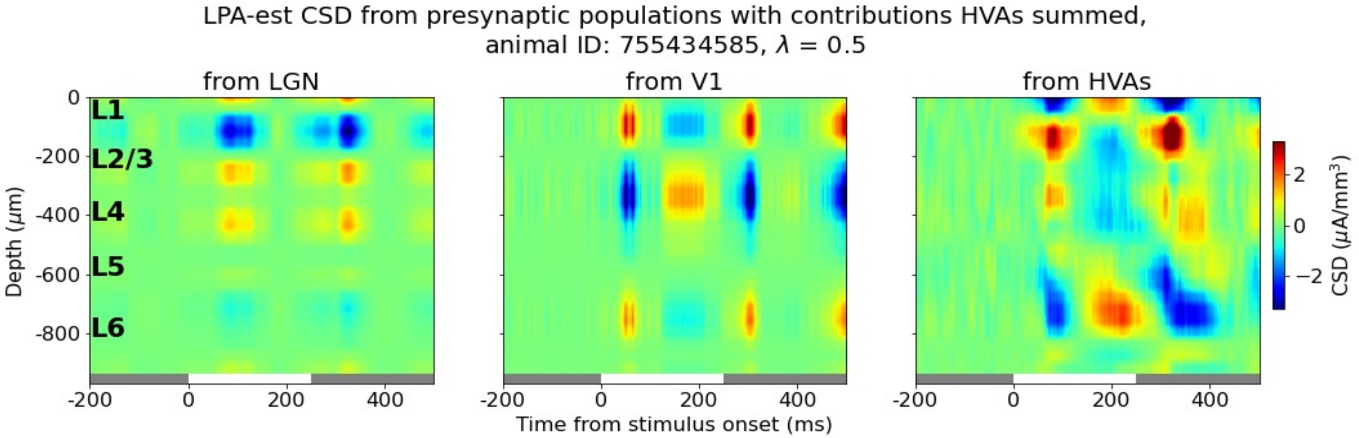

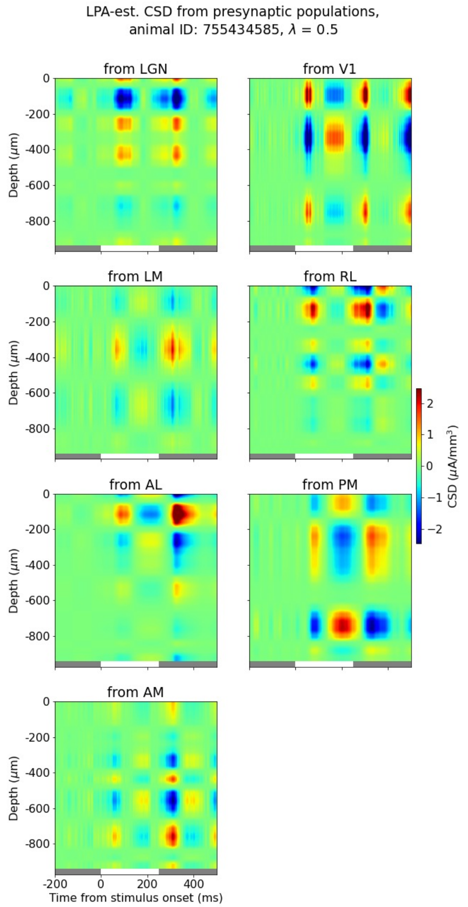

Session ID: 756029989

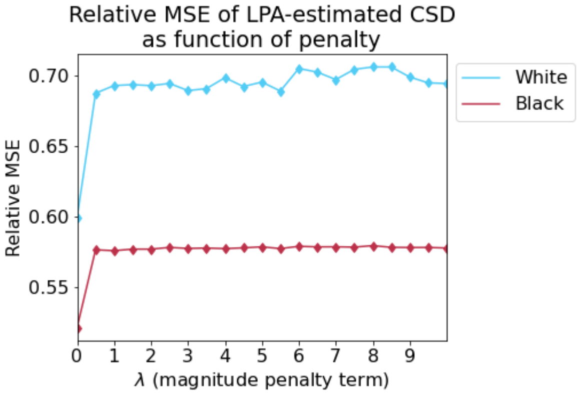

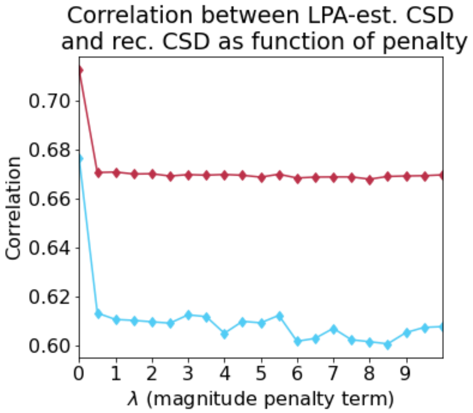

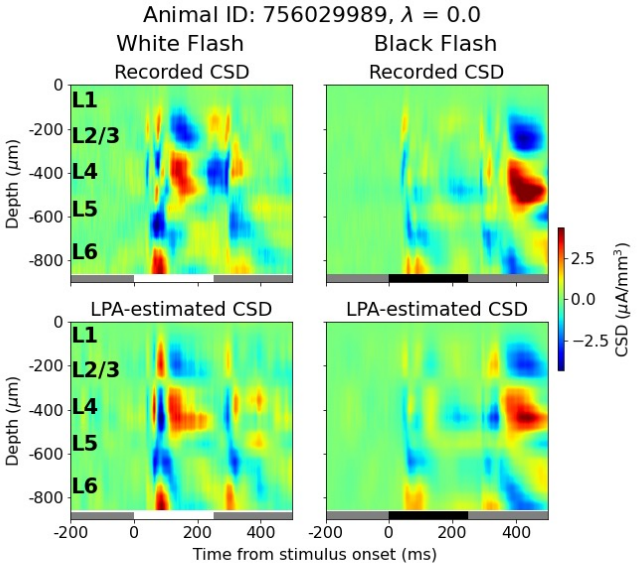

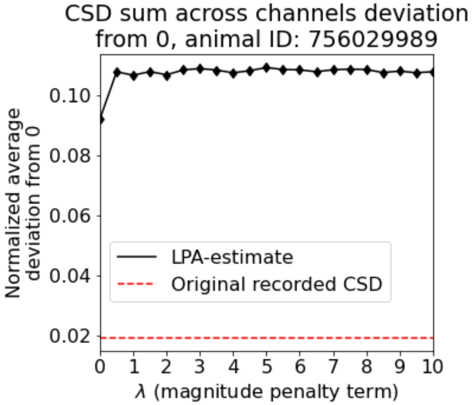

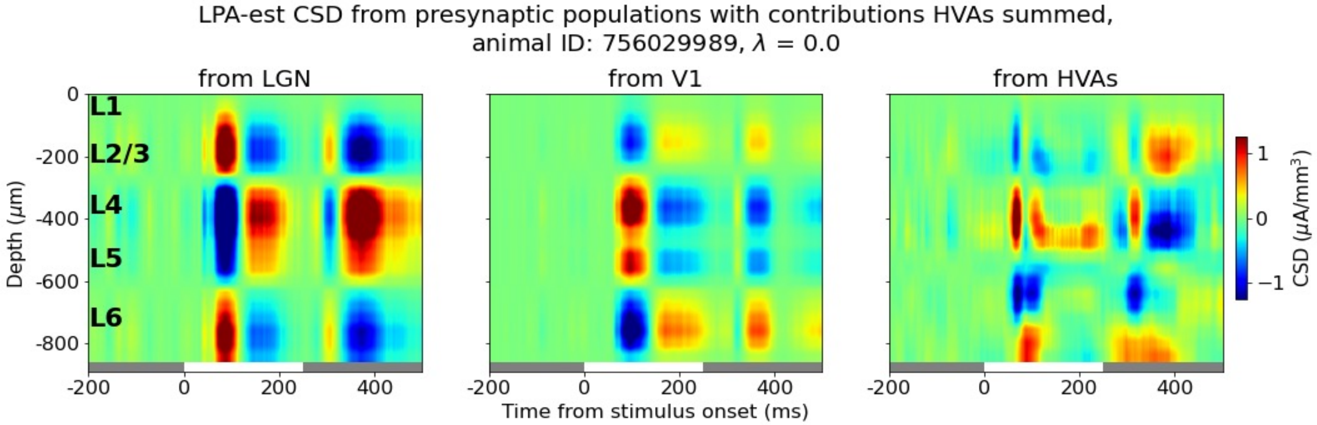

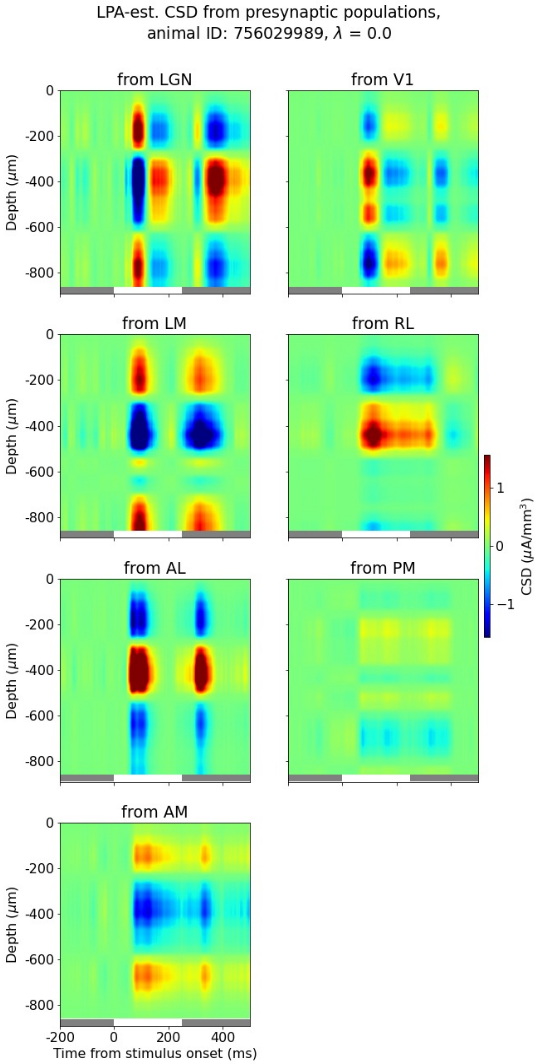

Session ID: 757970808

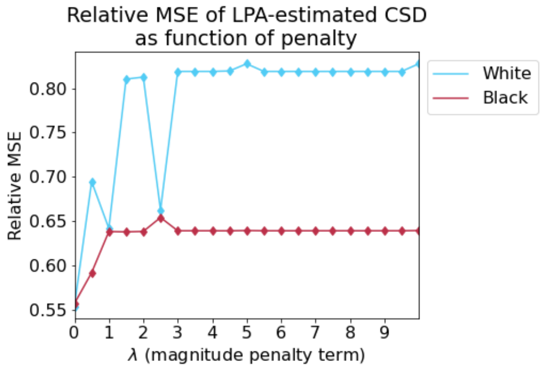

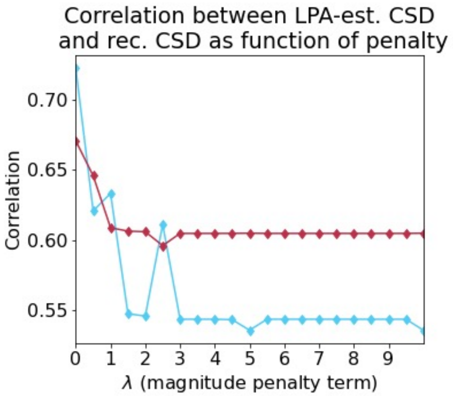

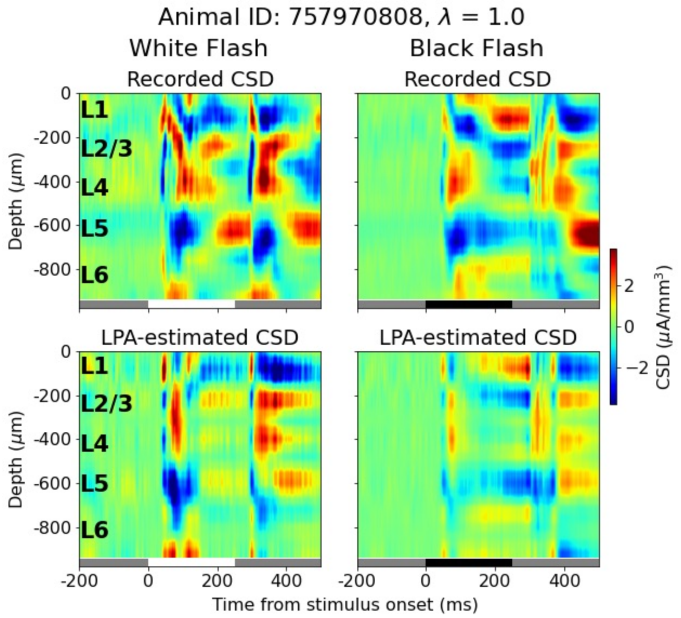

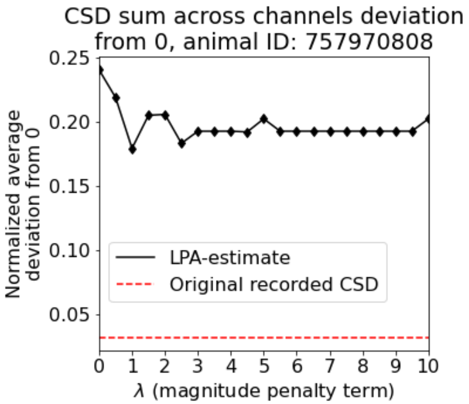

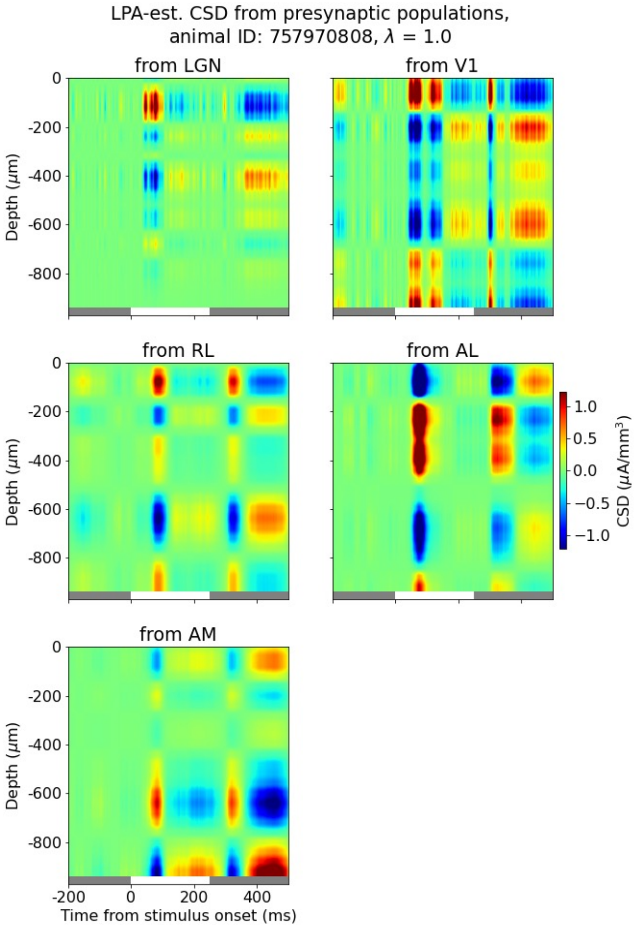

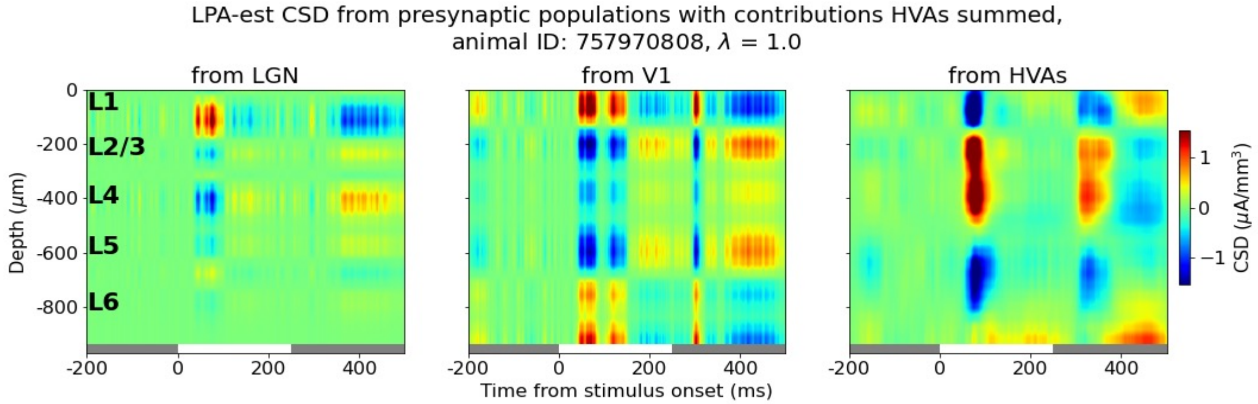

Session ID: 759883607

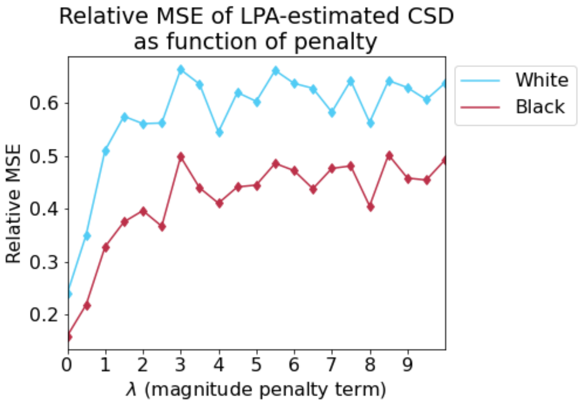

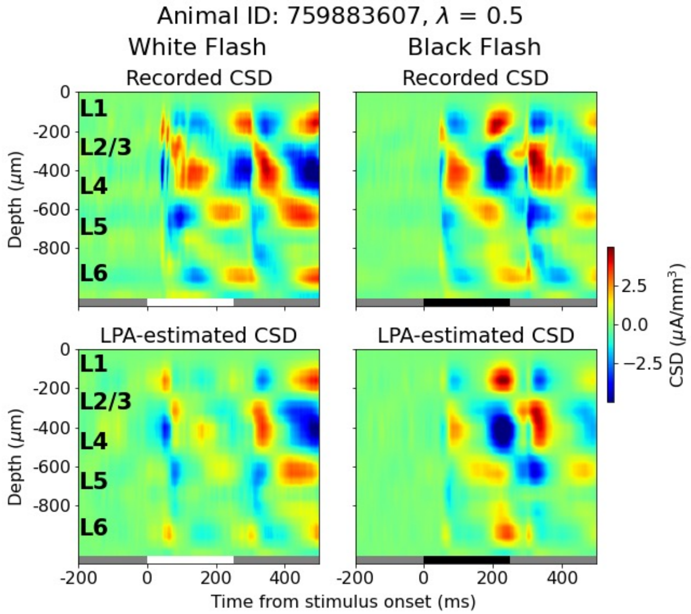

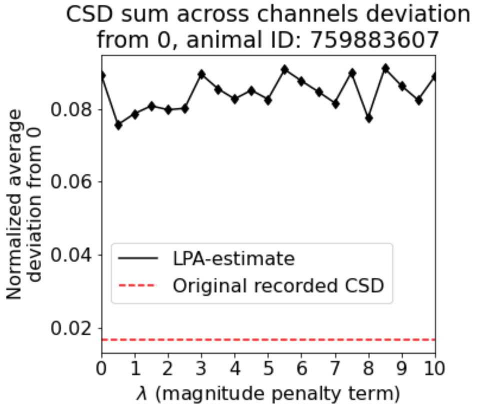

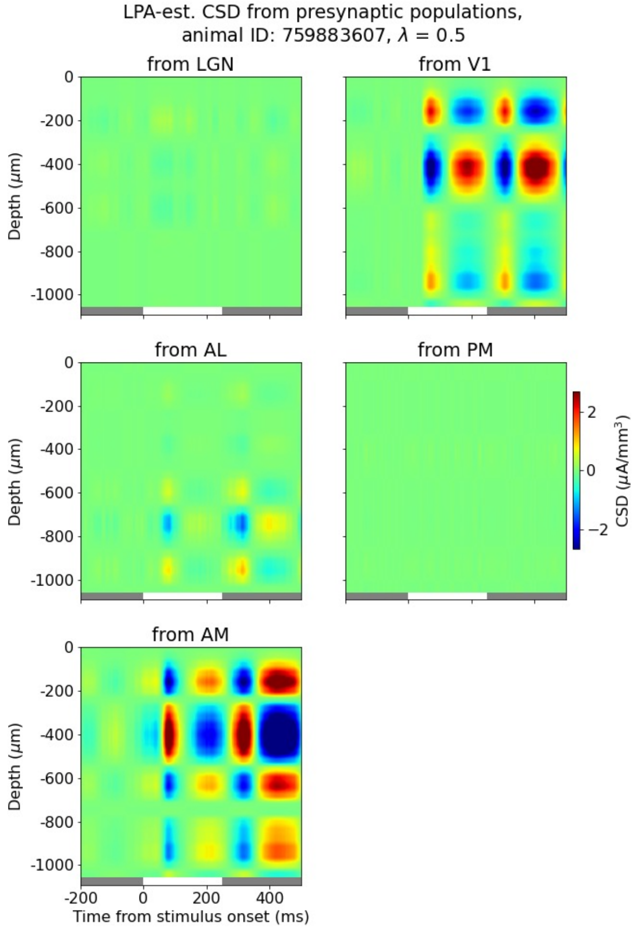

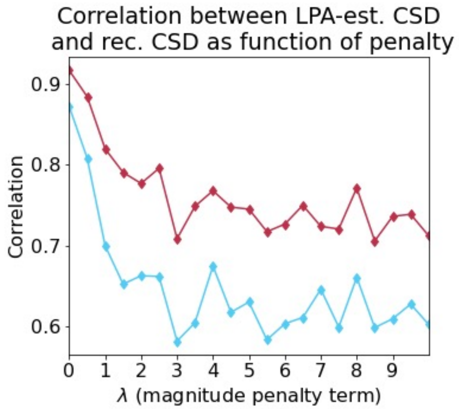

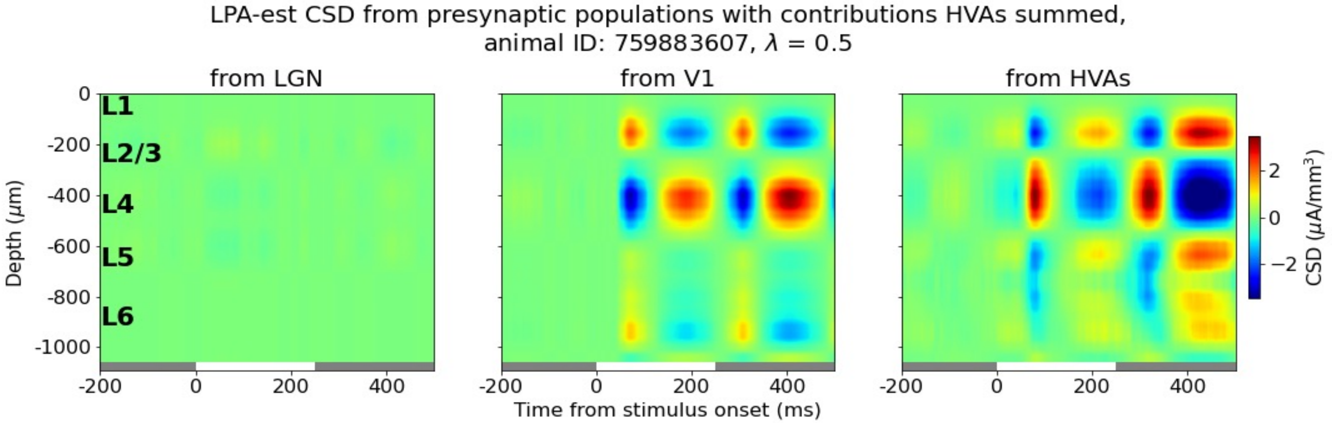

Session ID: 760345702

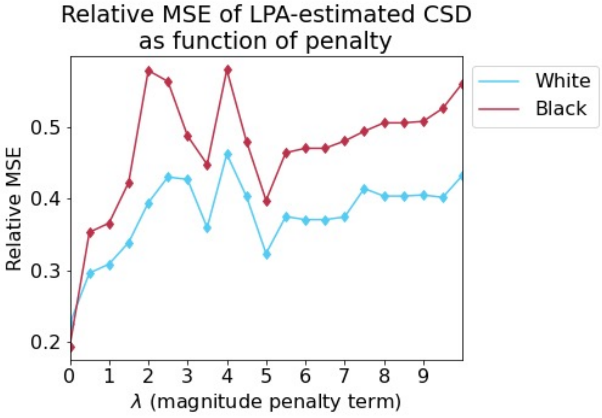

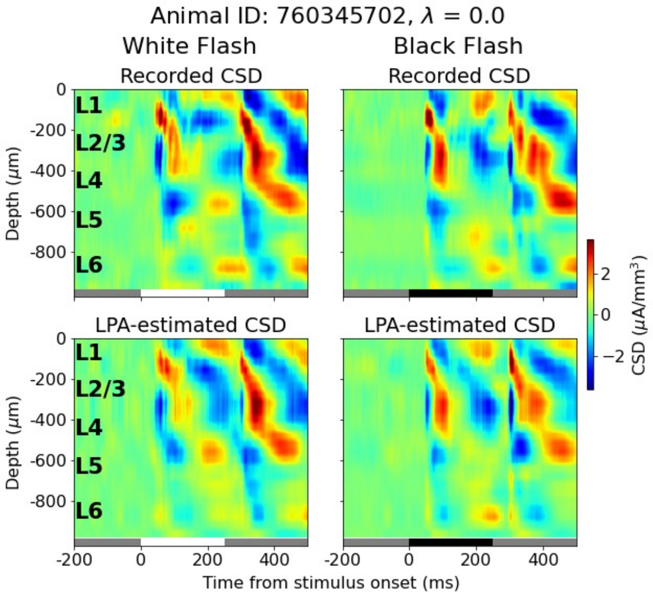

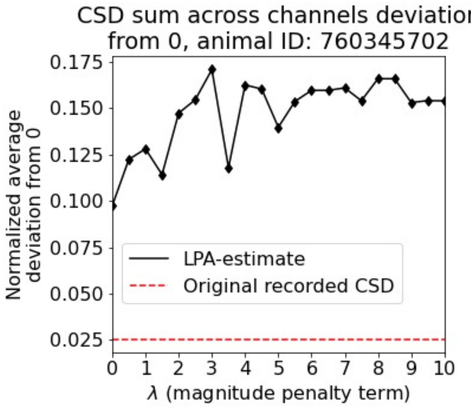

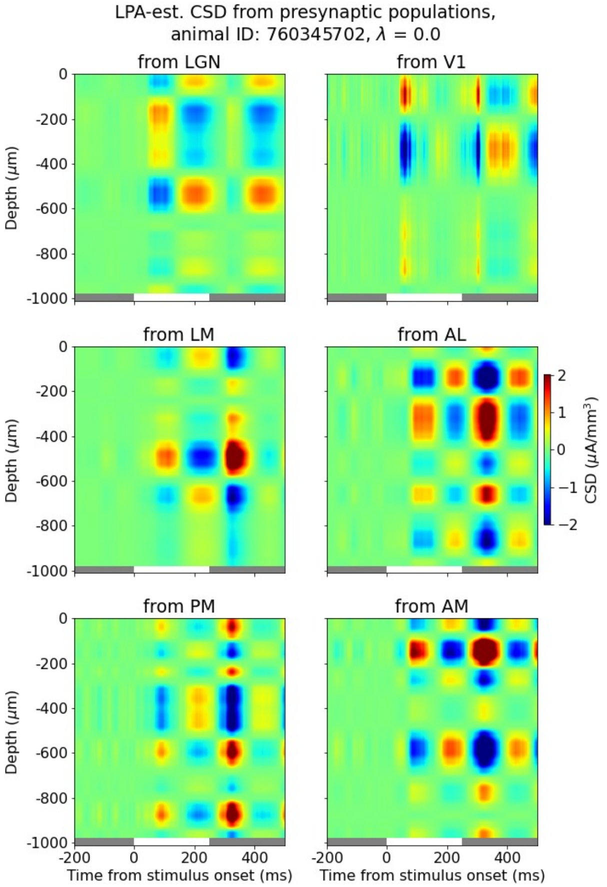

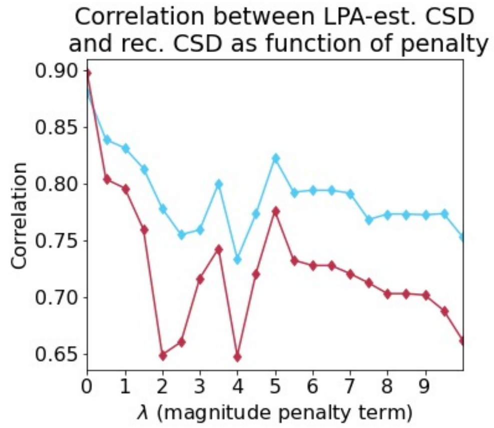

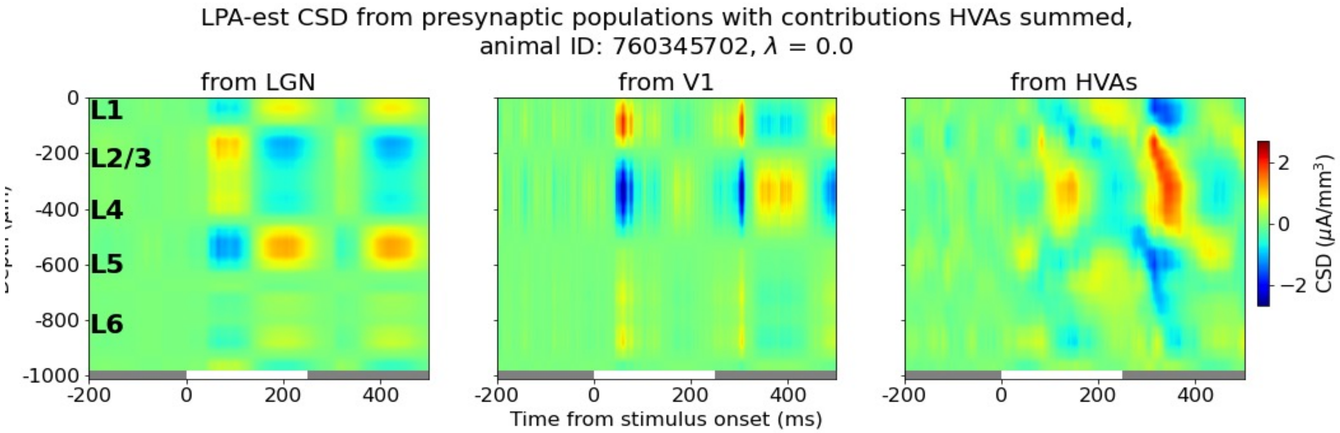

Session ID: 763673393

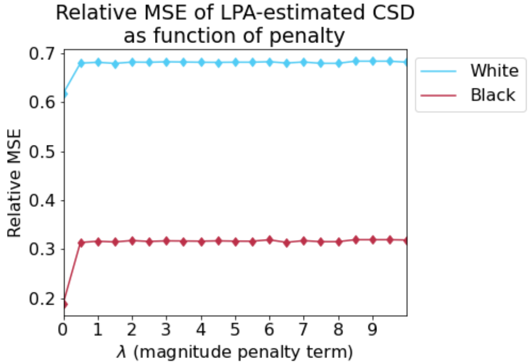

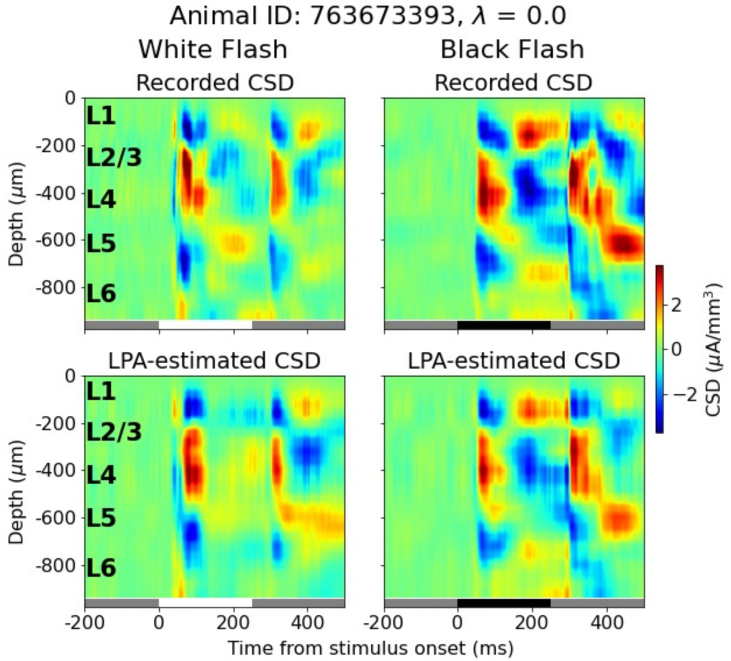

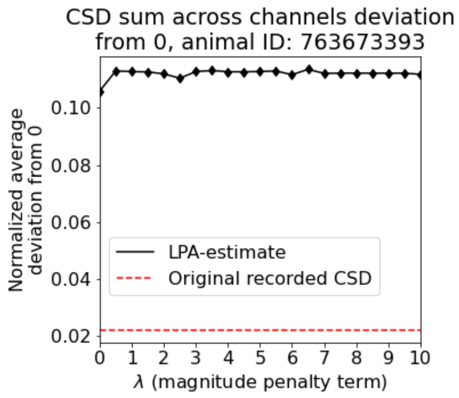

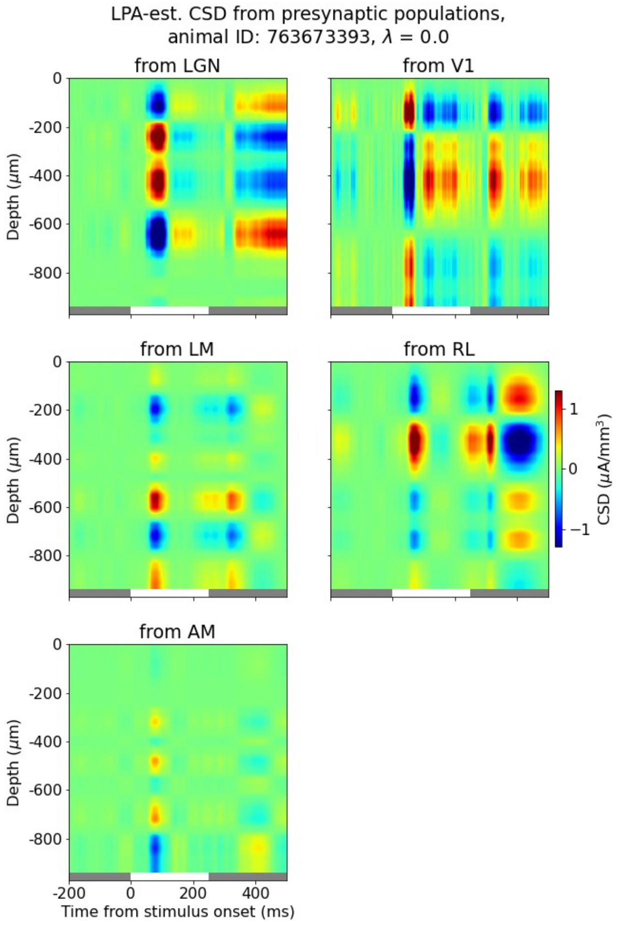

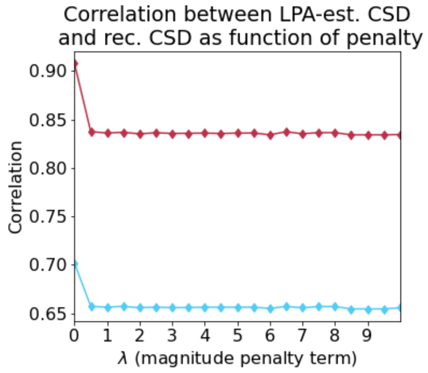

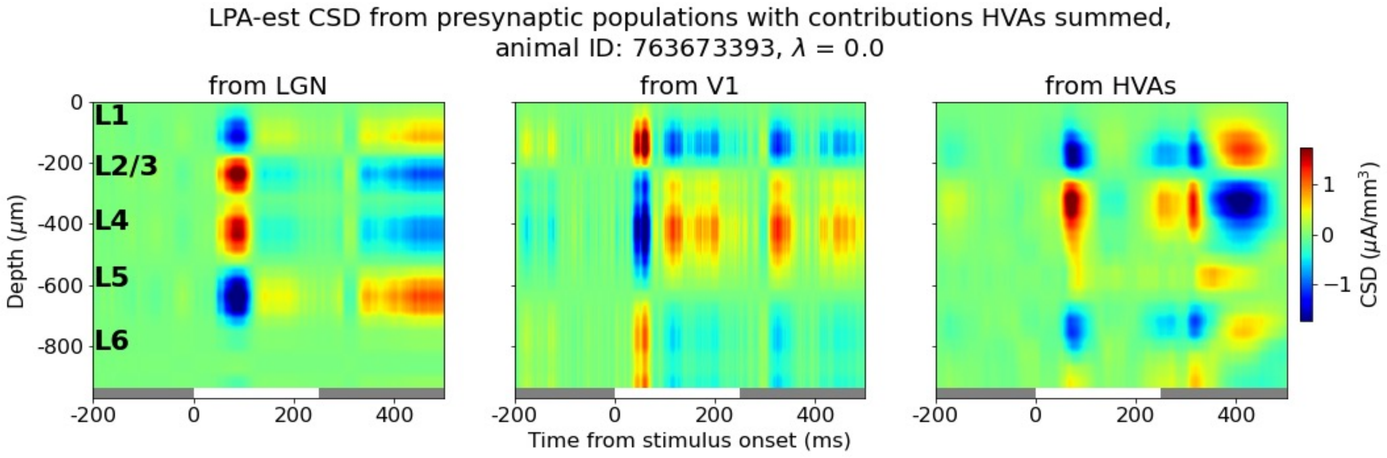

Session ID: 766640955

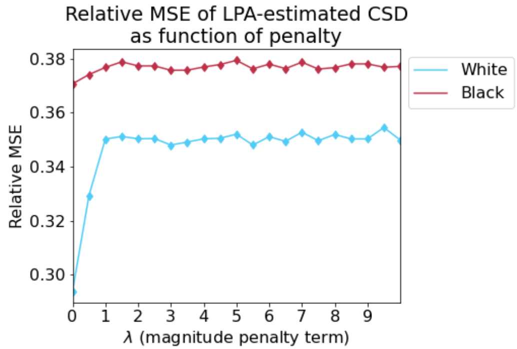

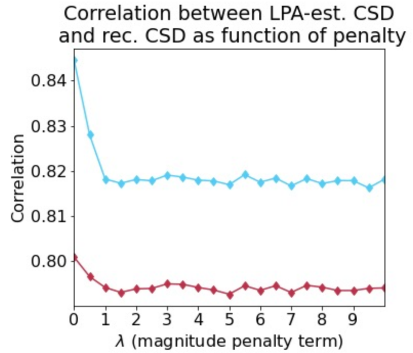

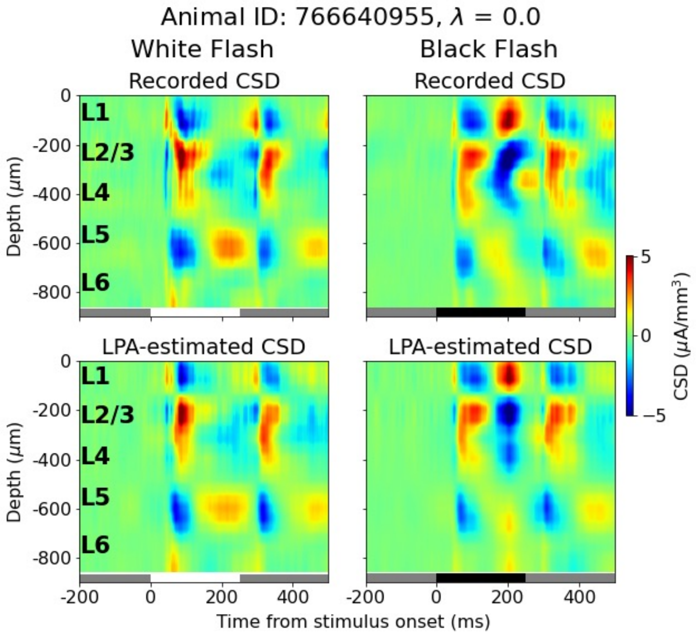

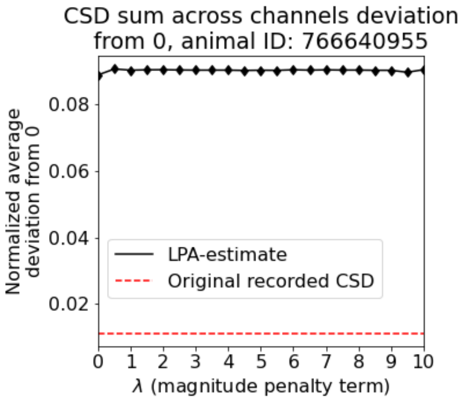

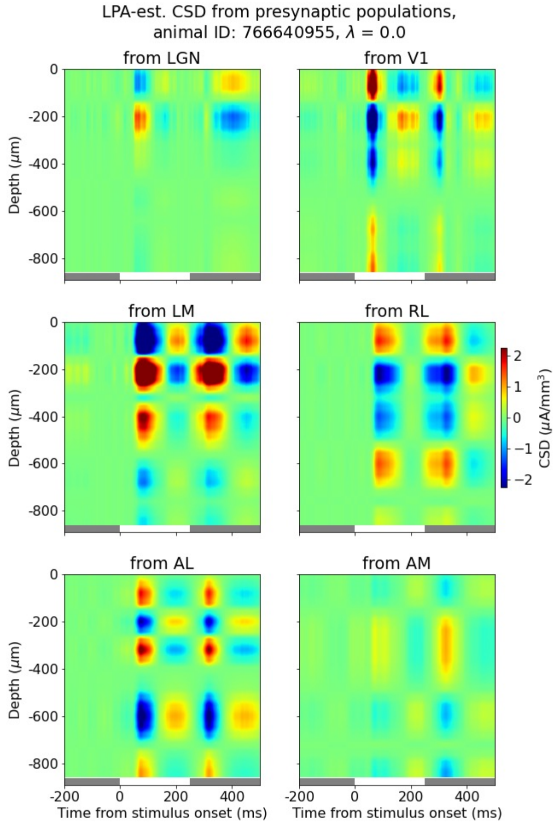

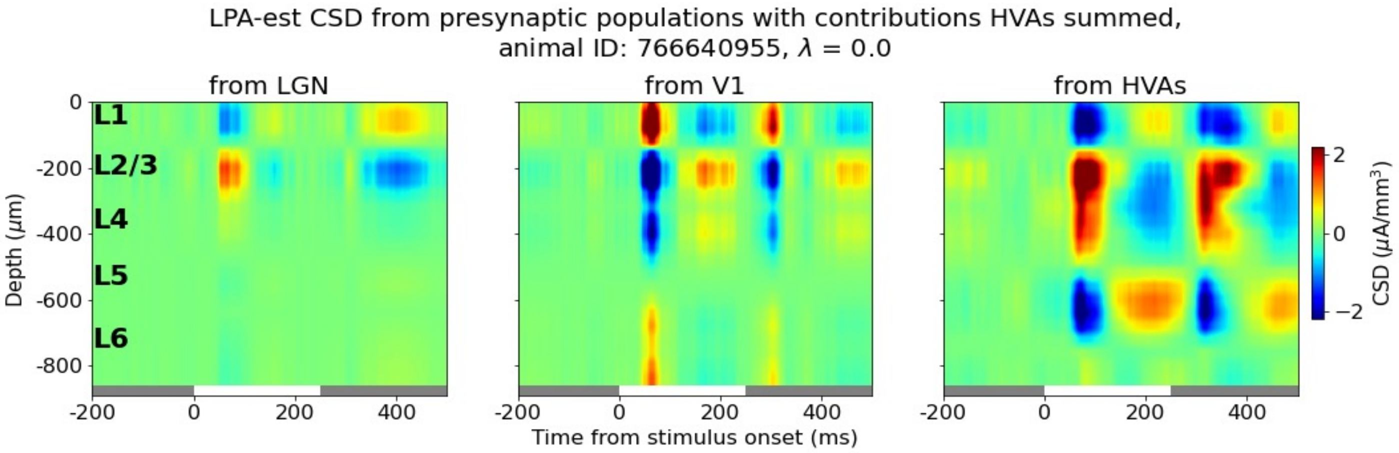

Session ID: 768515987

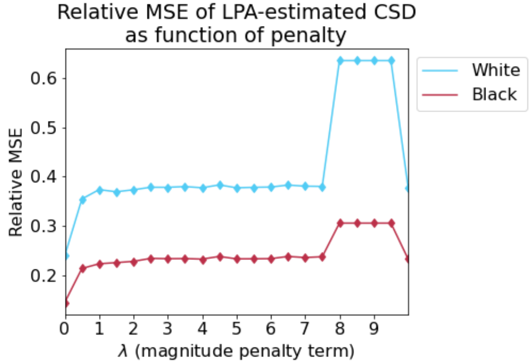

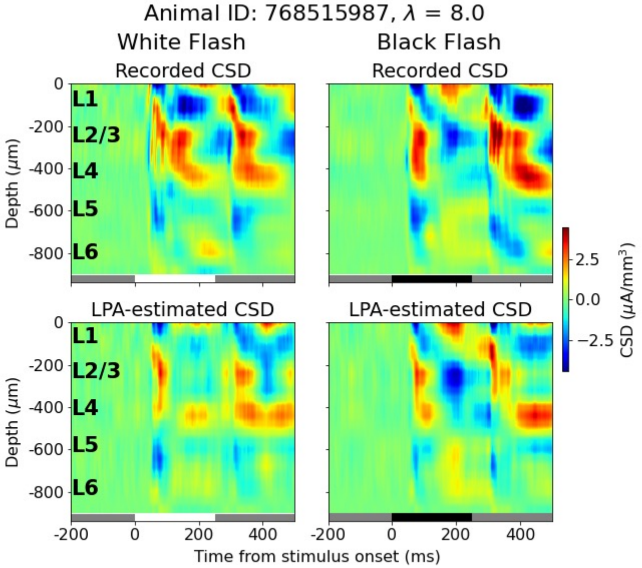

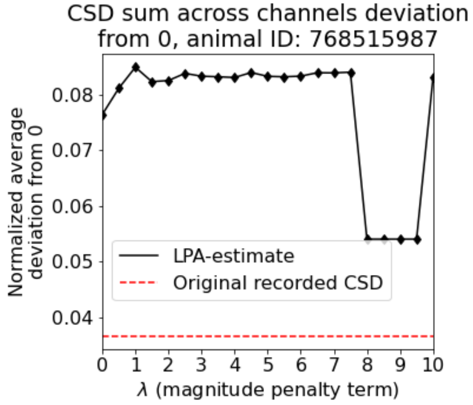

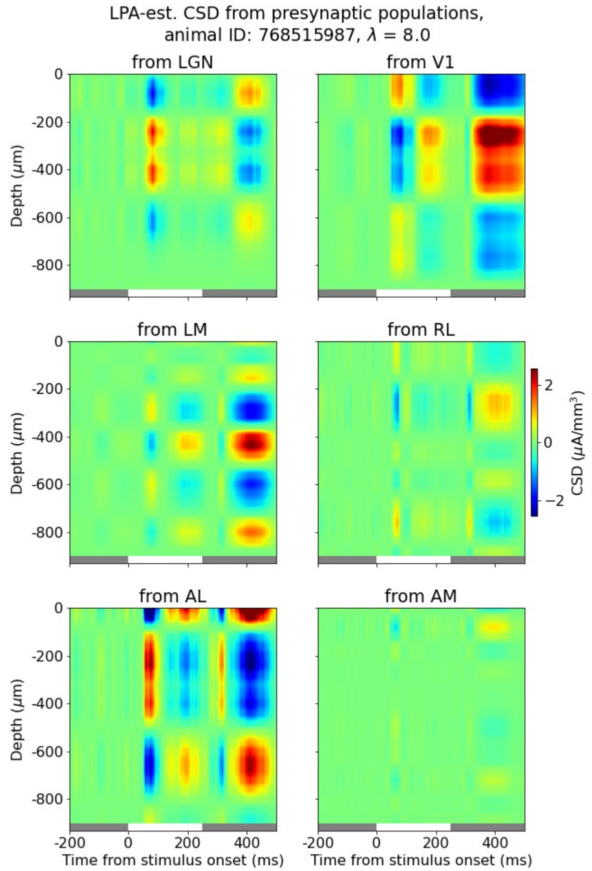

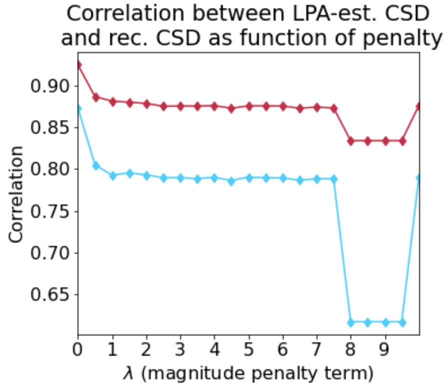

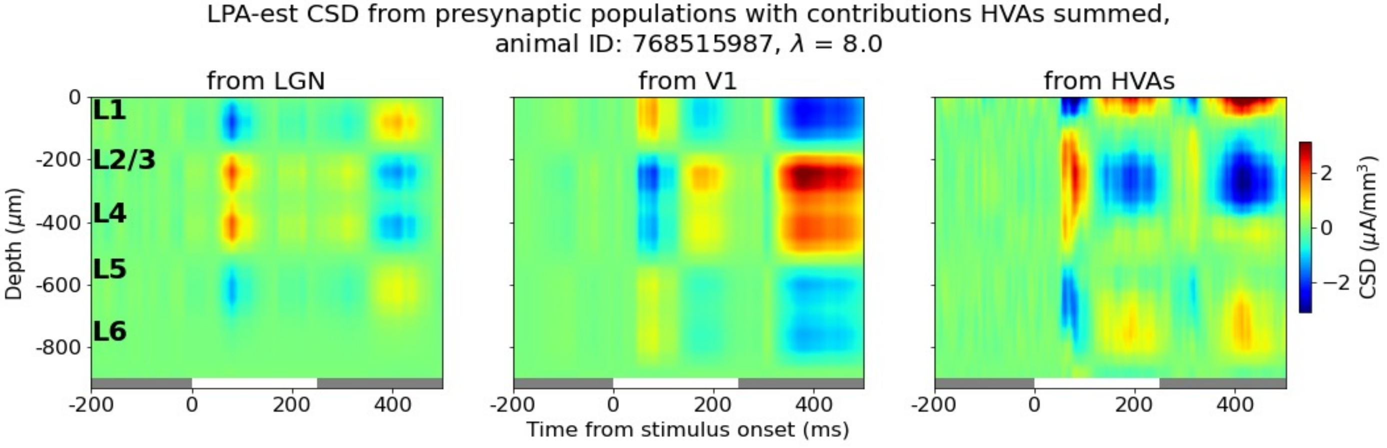

Session ID: 771160300

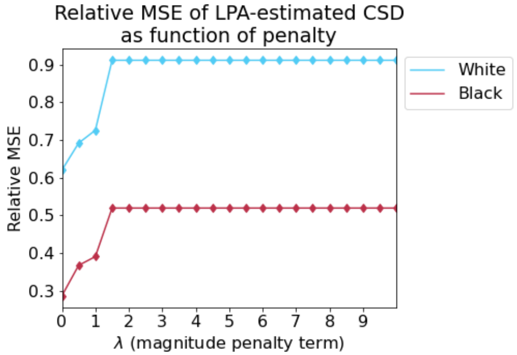

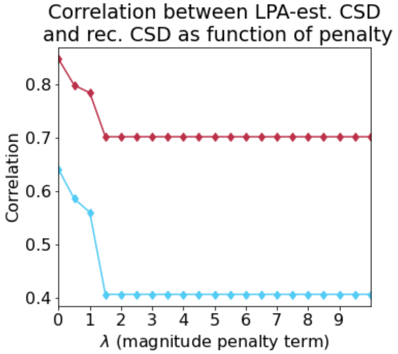

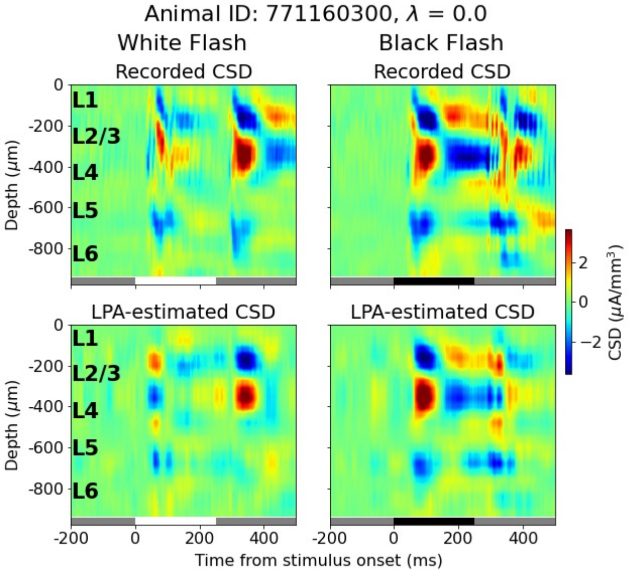

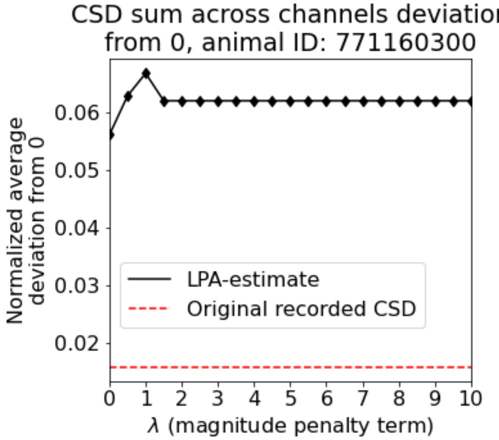

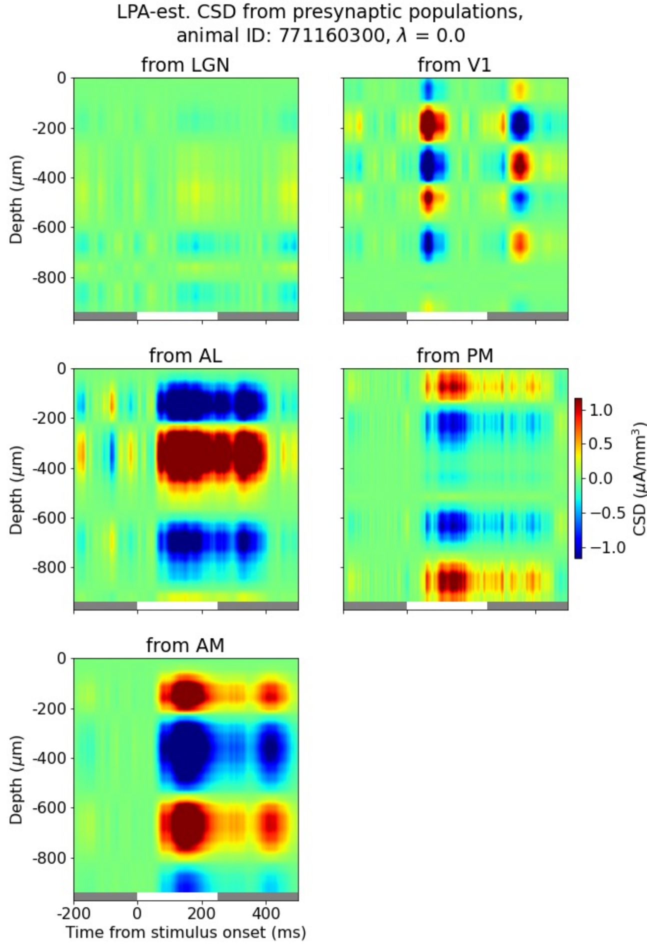

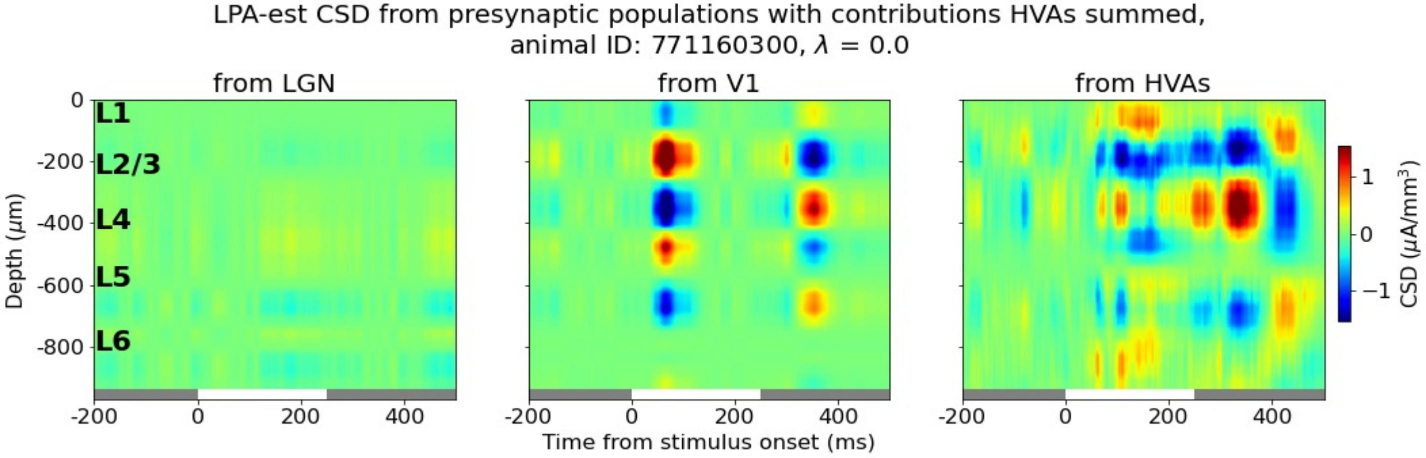

Session ID: 771990200

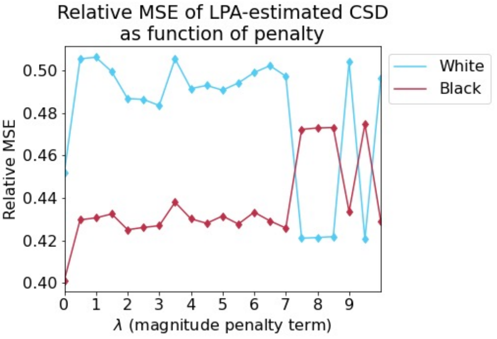

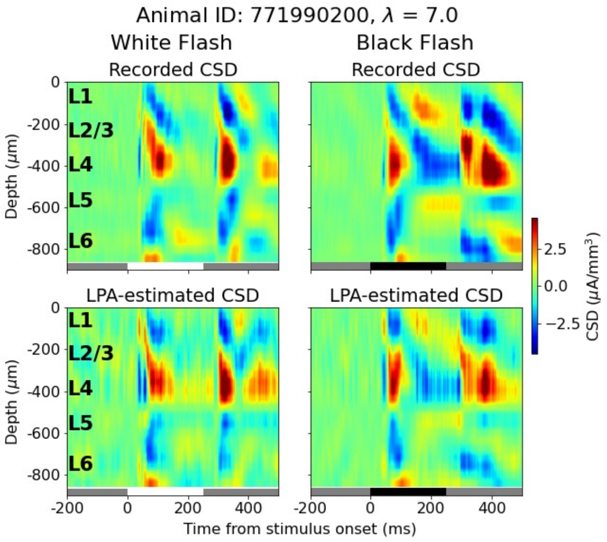

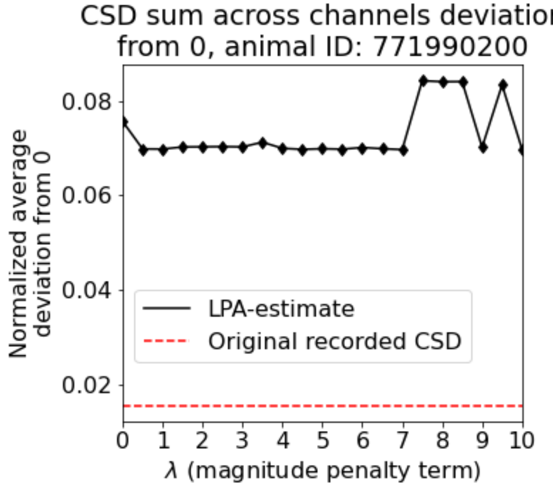

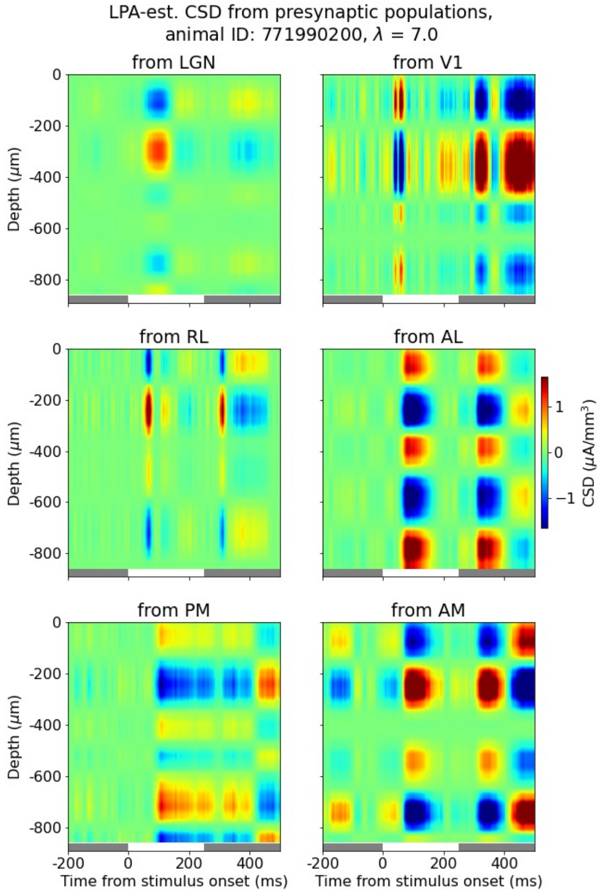

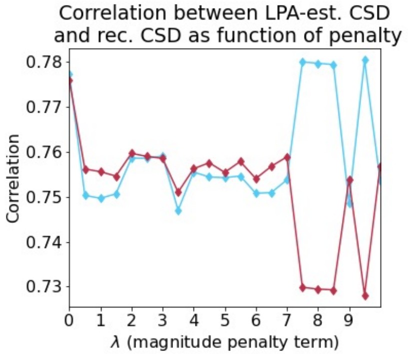

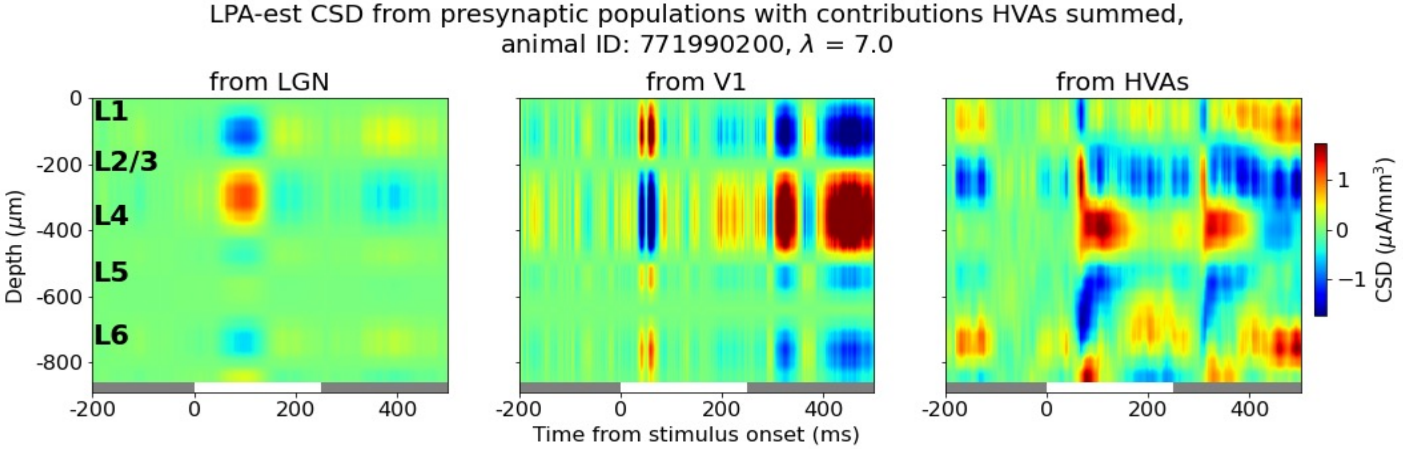

Session ID: 778240327

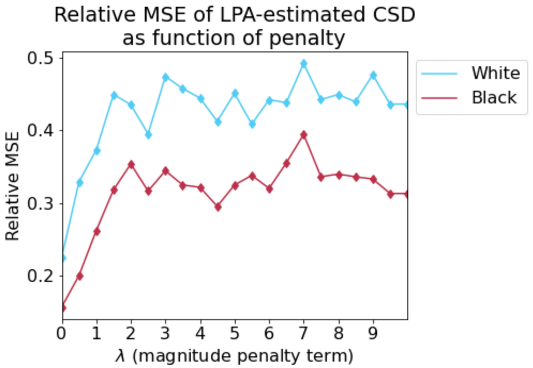

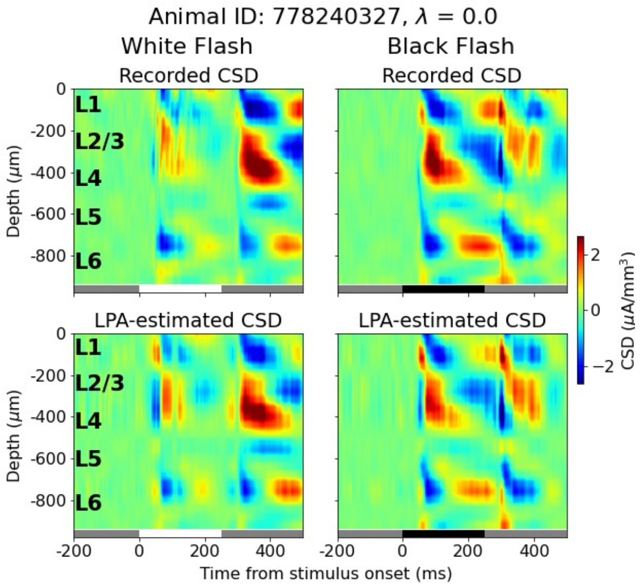

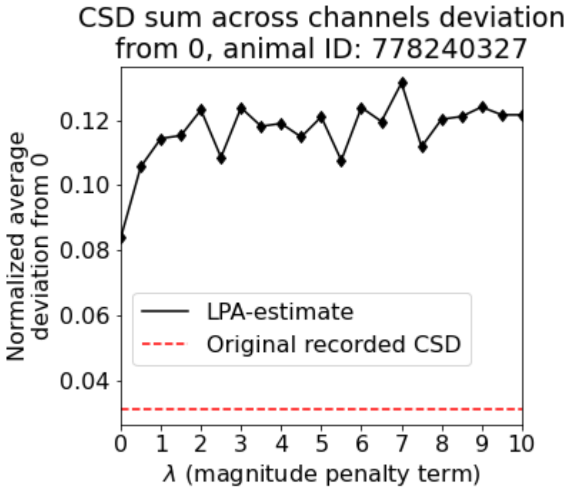

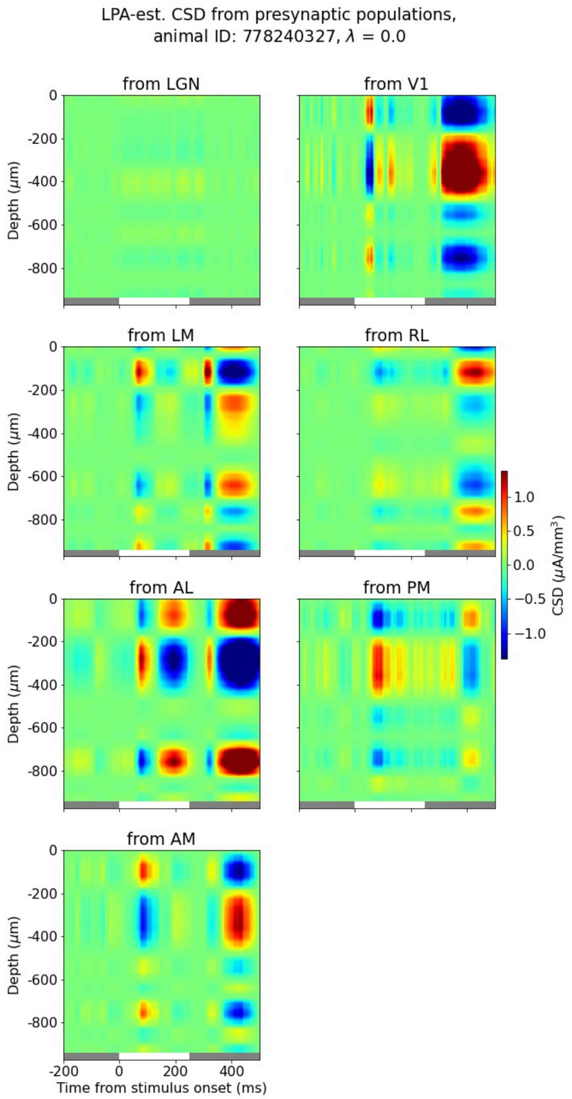

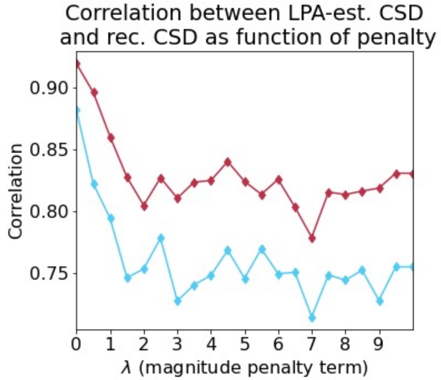

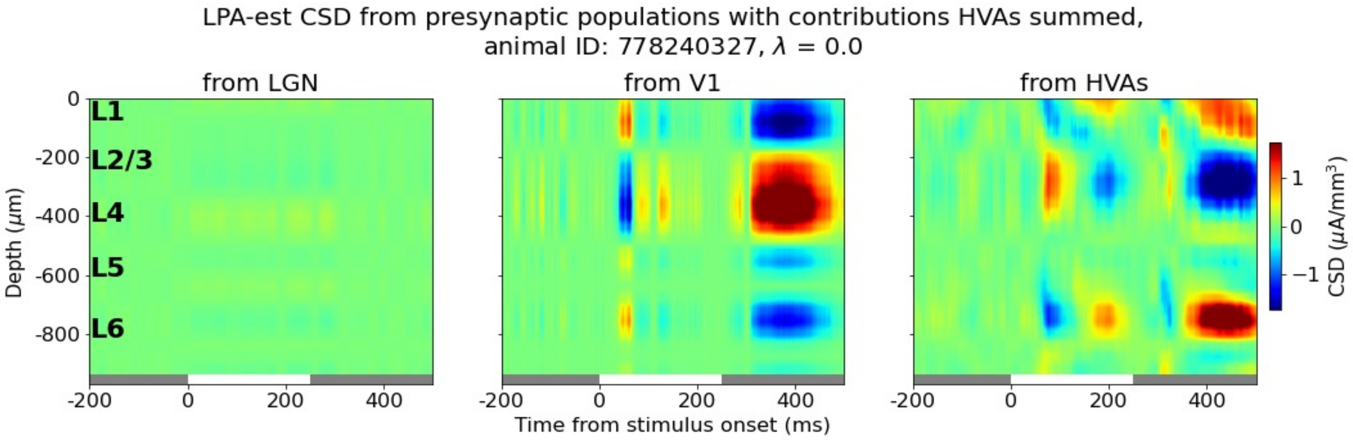

Session ID: 778240327

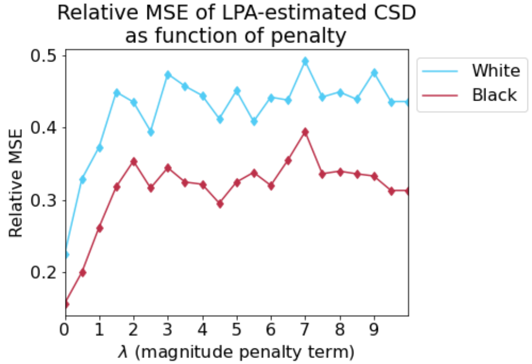

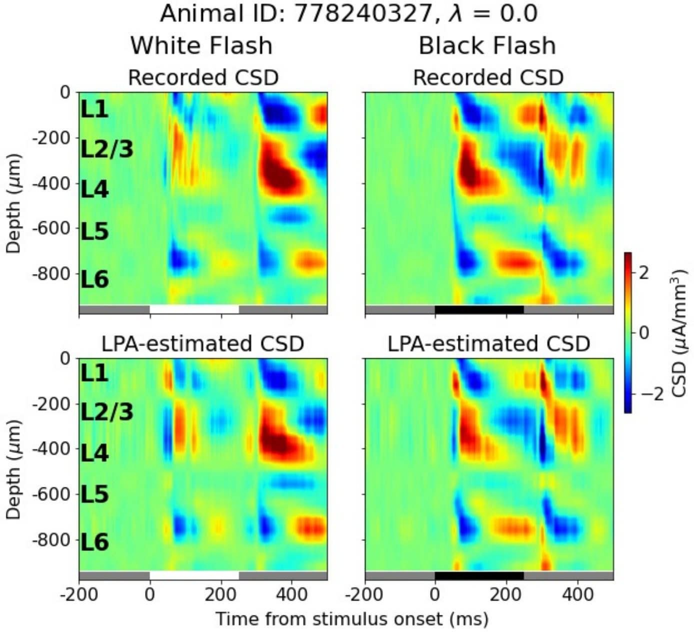

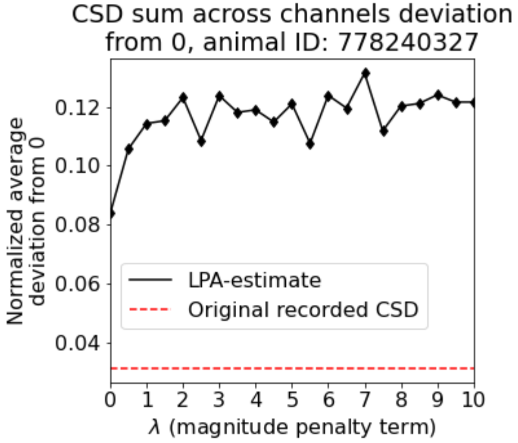

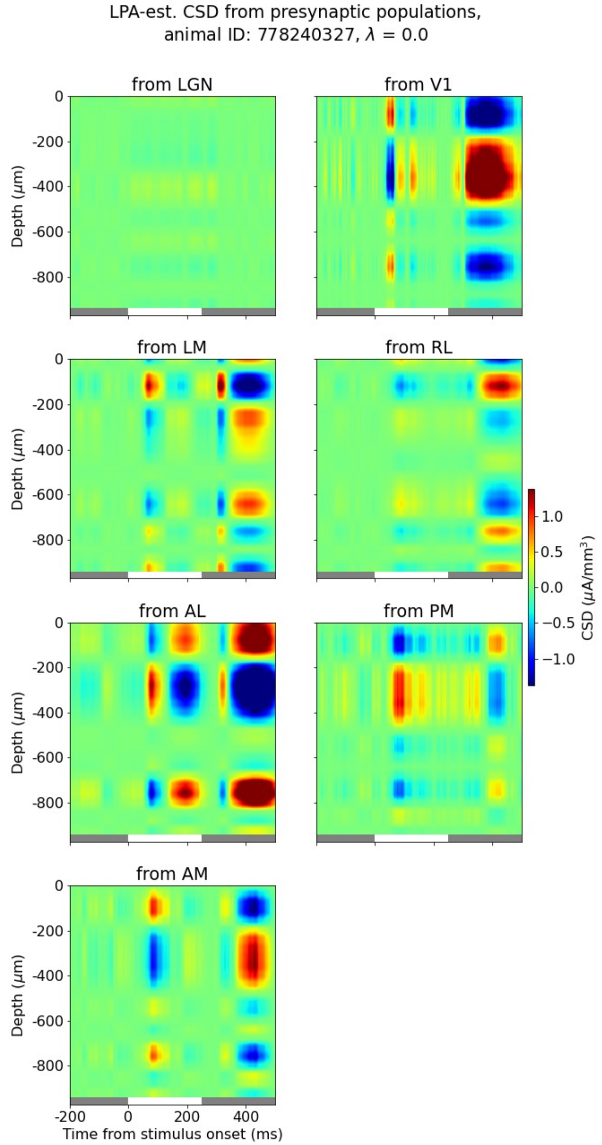

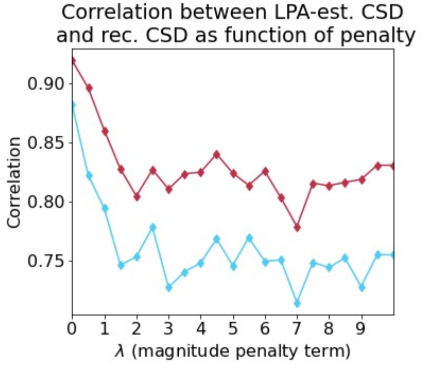

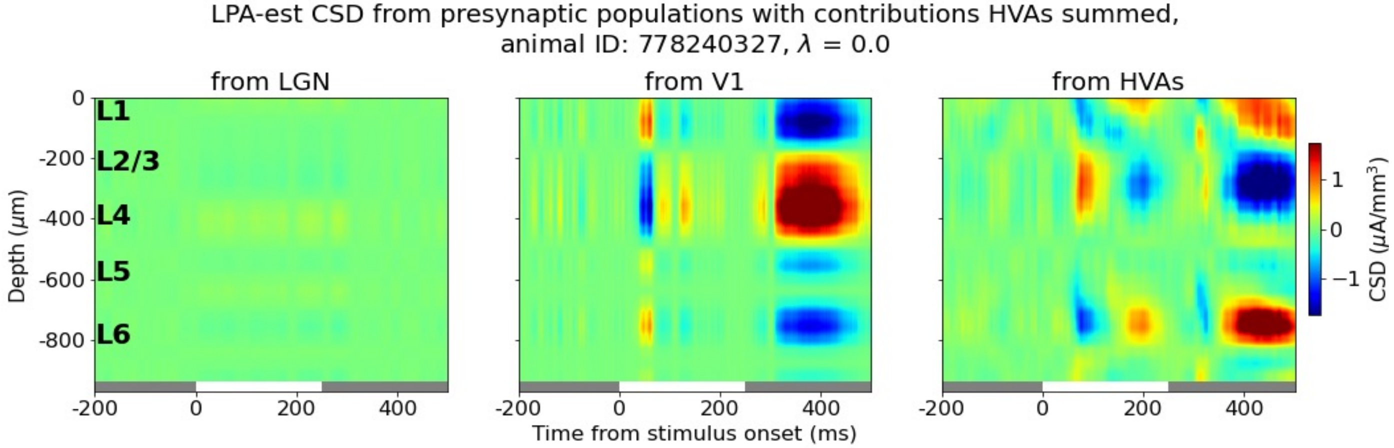

Session ID: 779839471

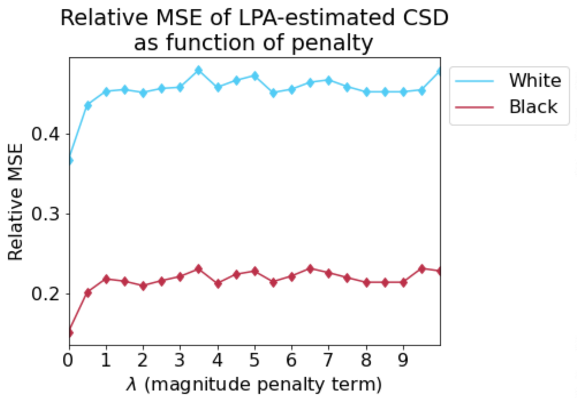

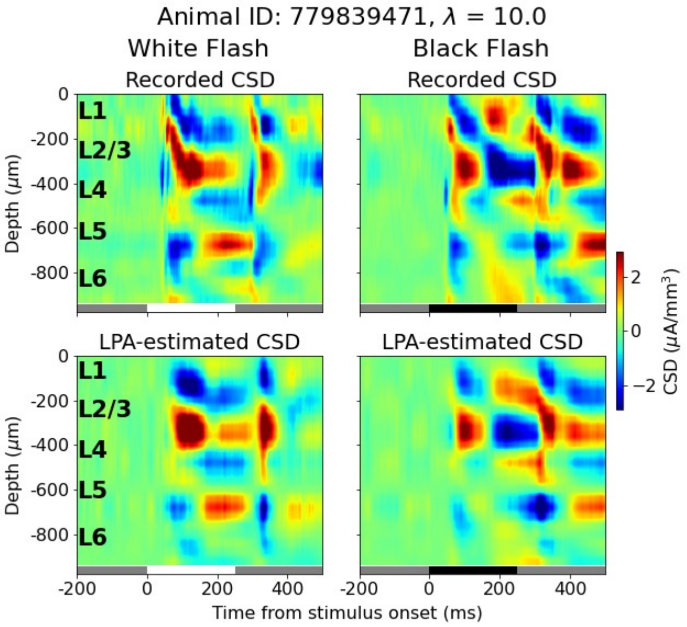

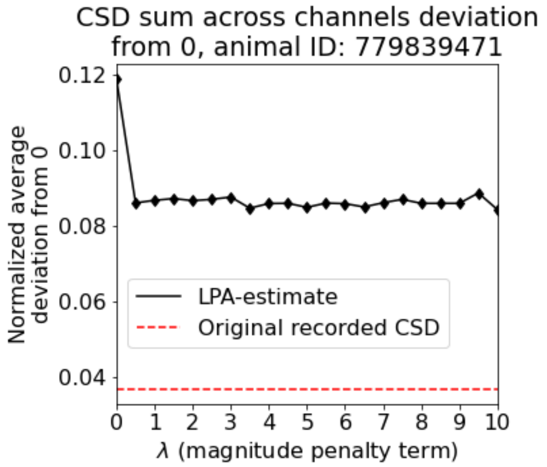

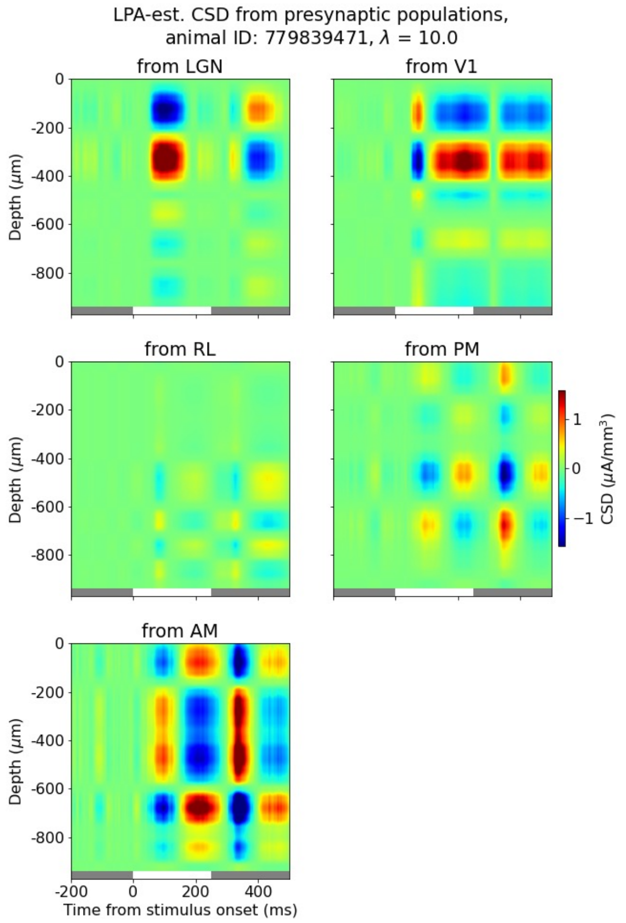

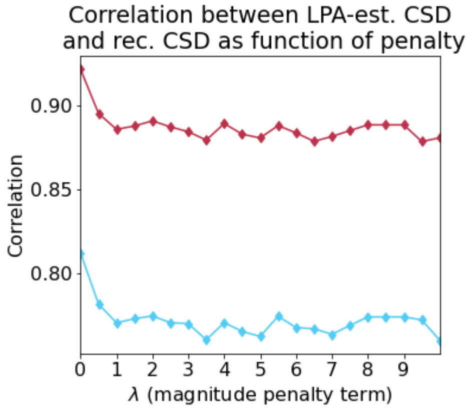

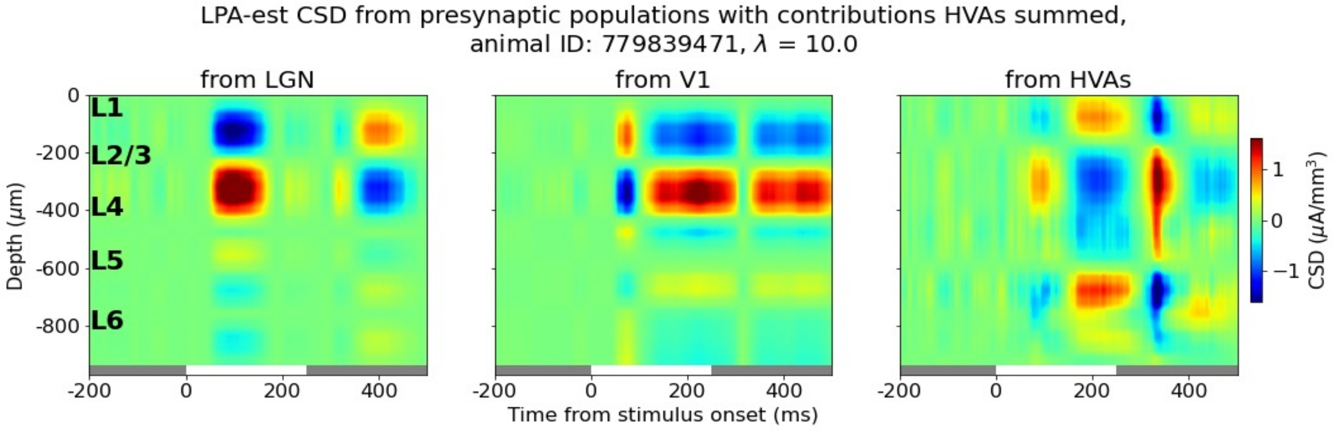

Session ID: 781842082

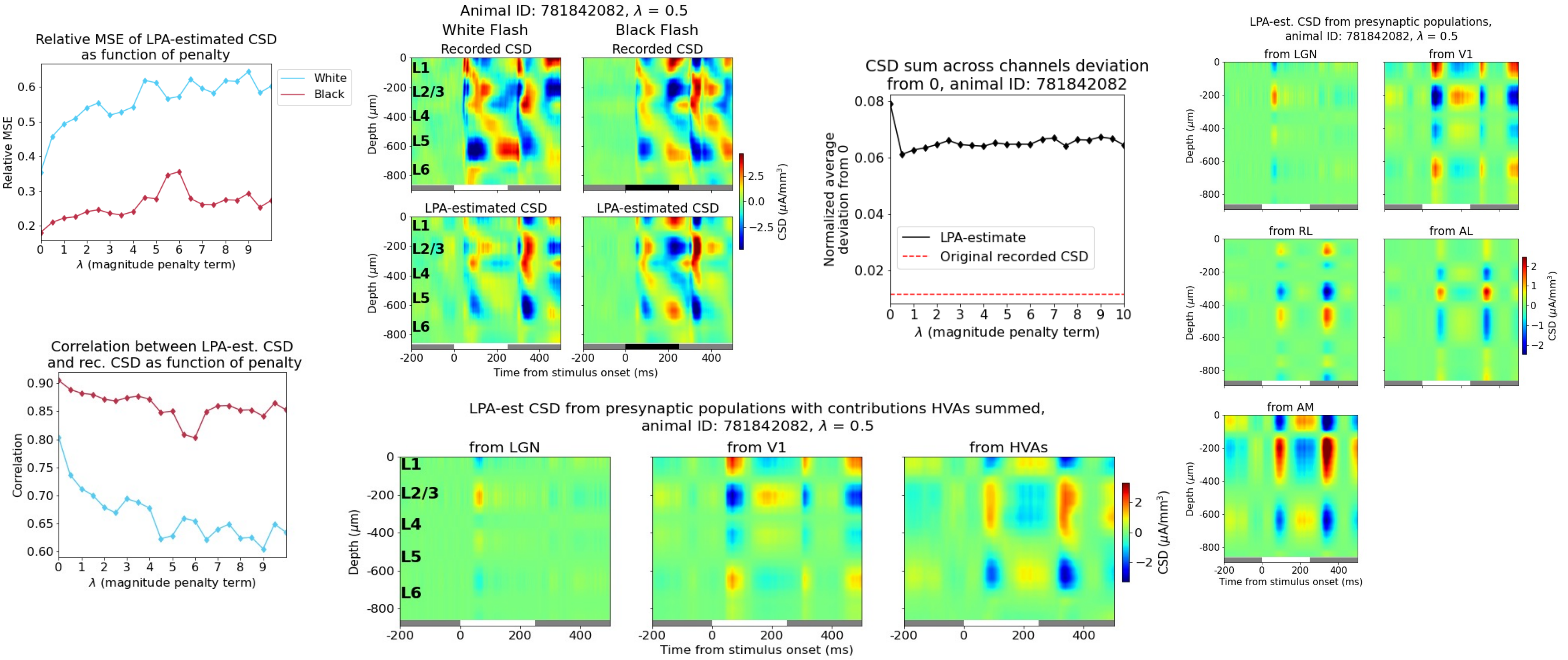

Session ID: 791319847

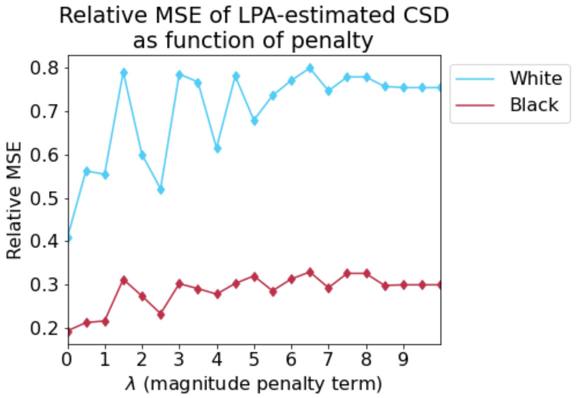

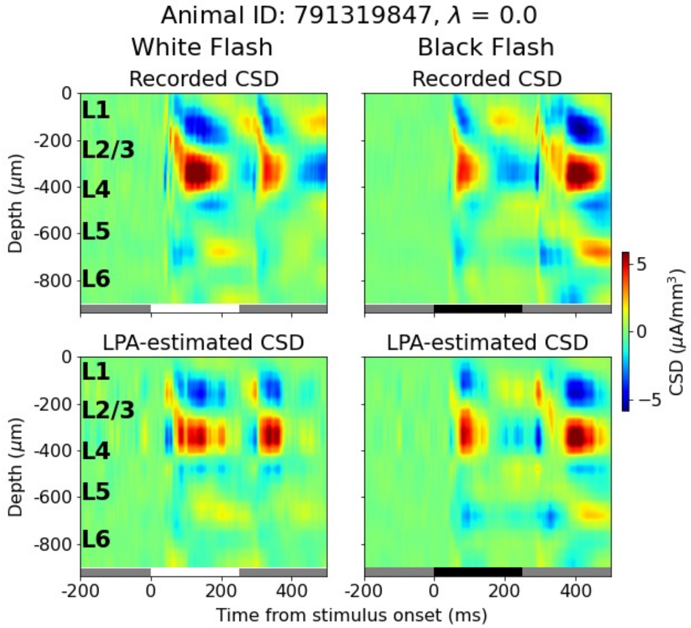

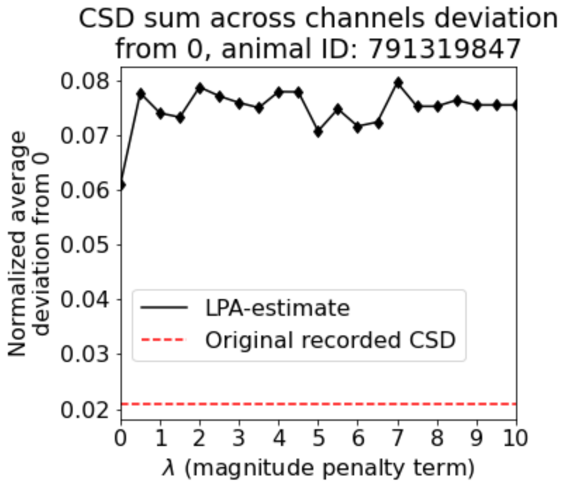

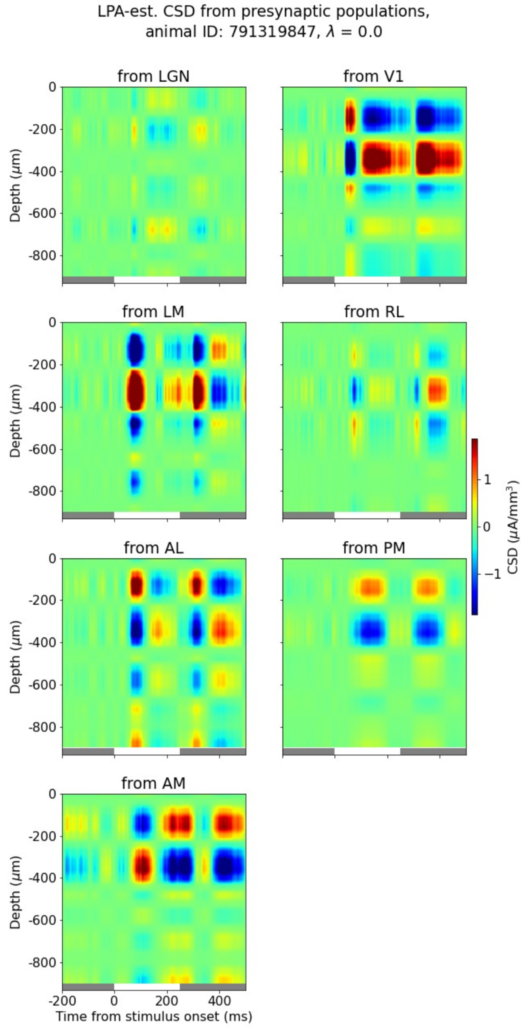

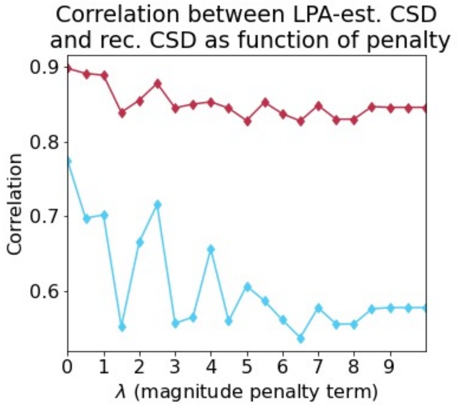

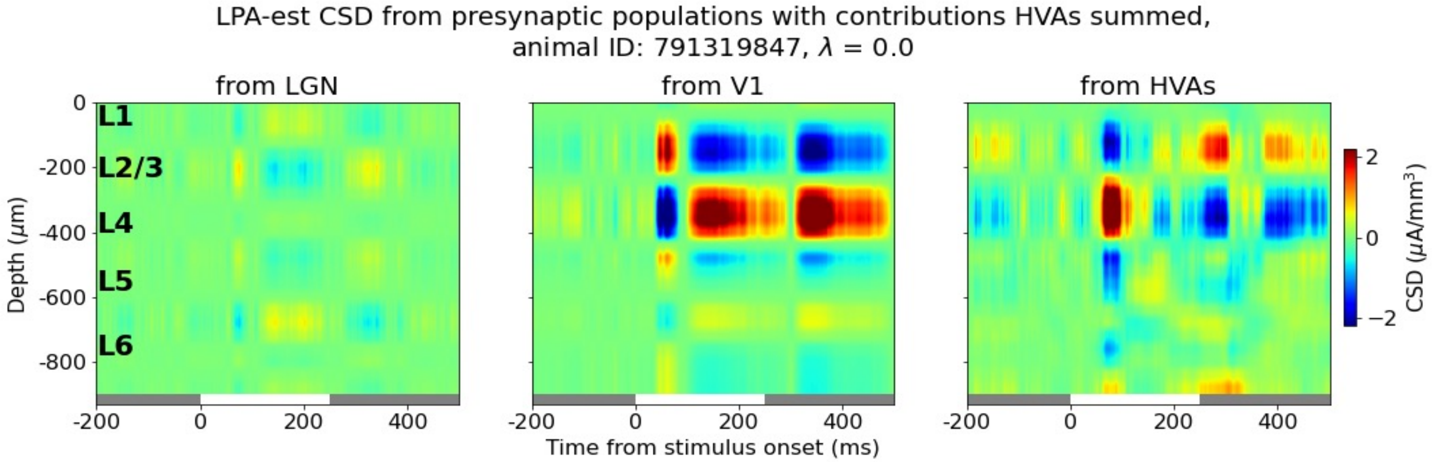

Session ID: 799864342

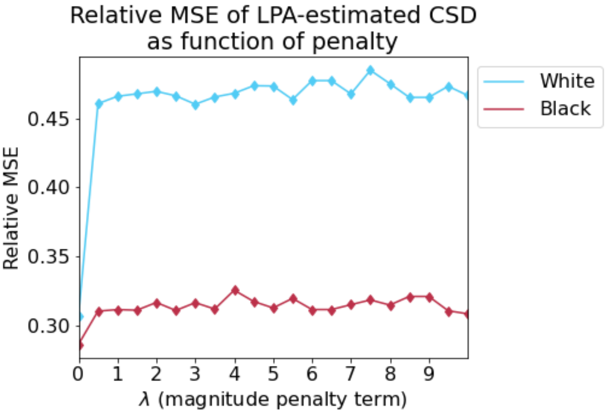

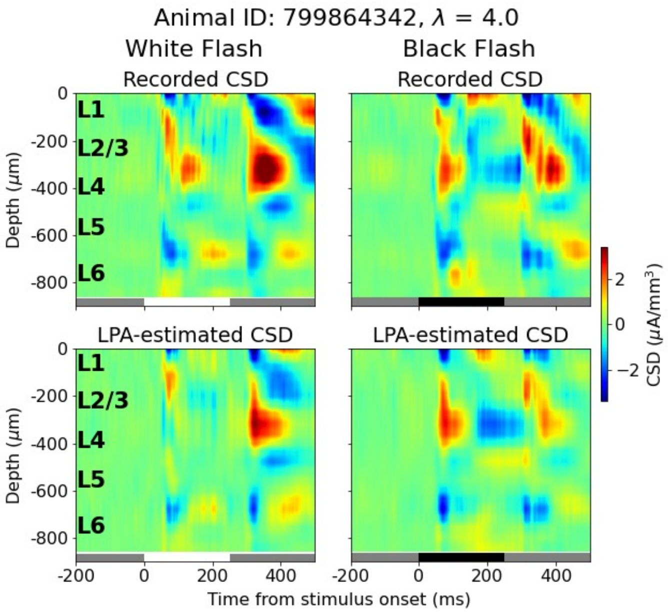

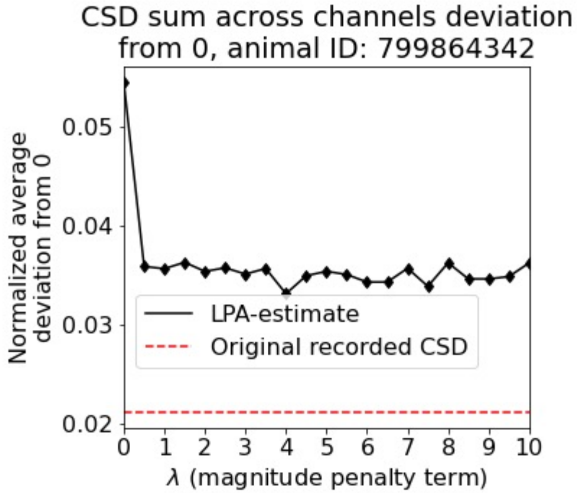

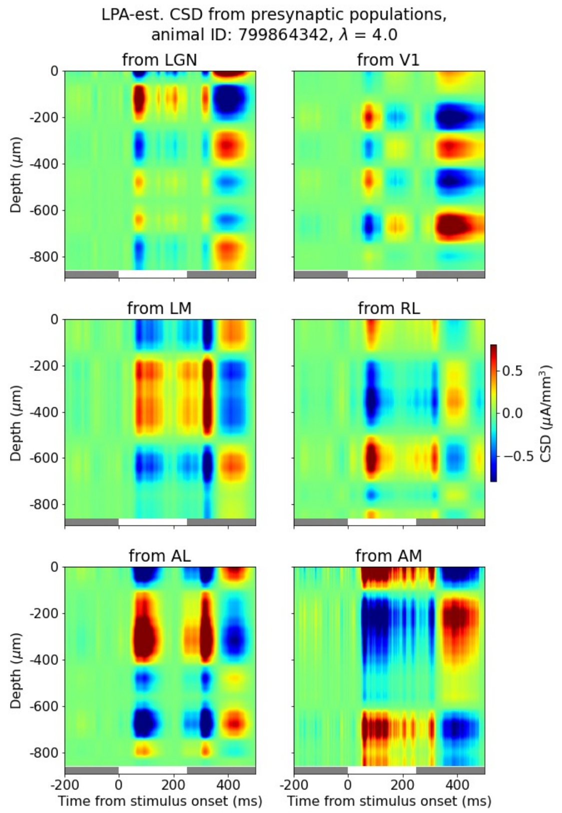

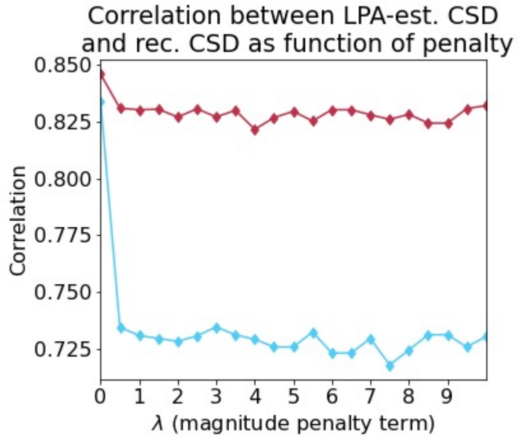

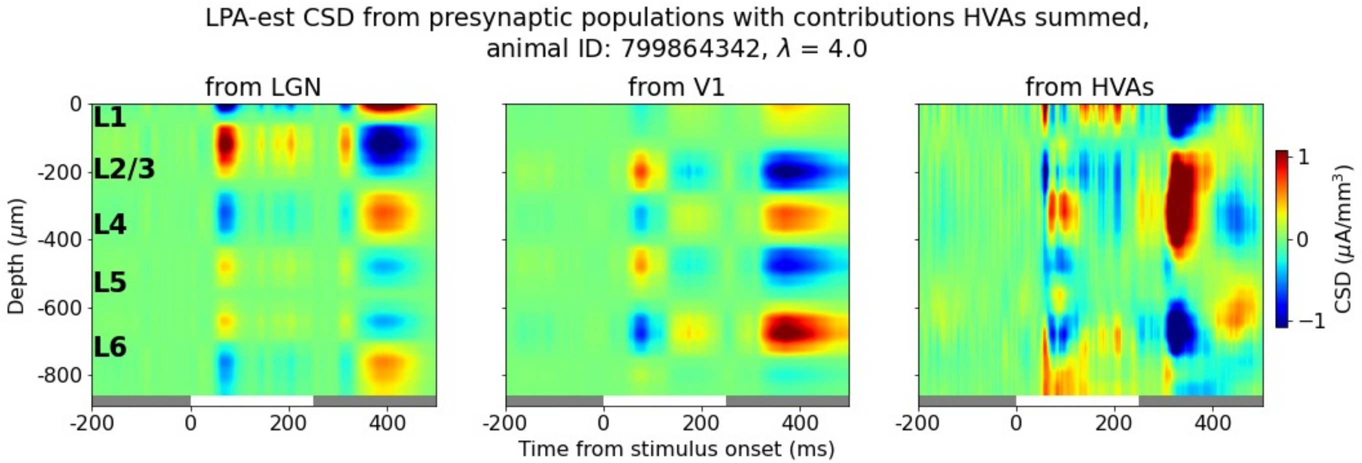

Session ID: 835479236

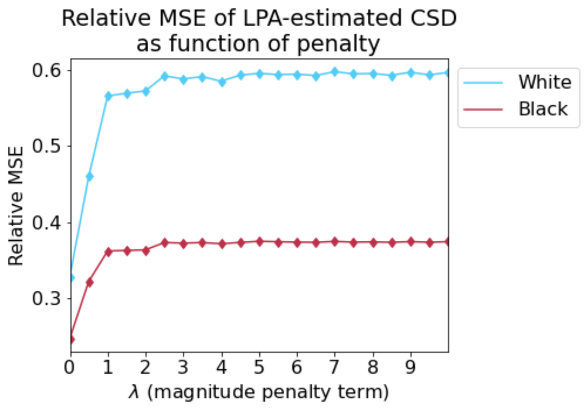

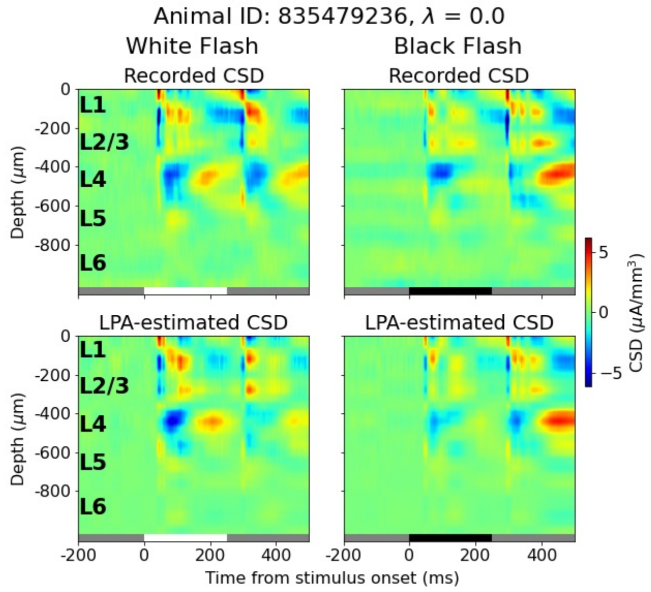

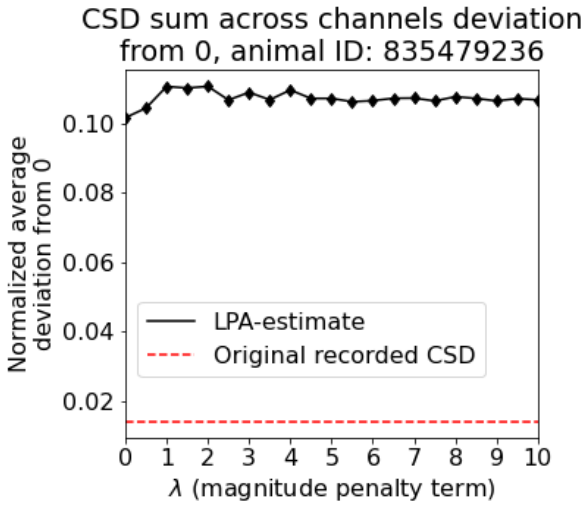

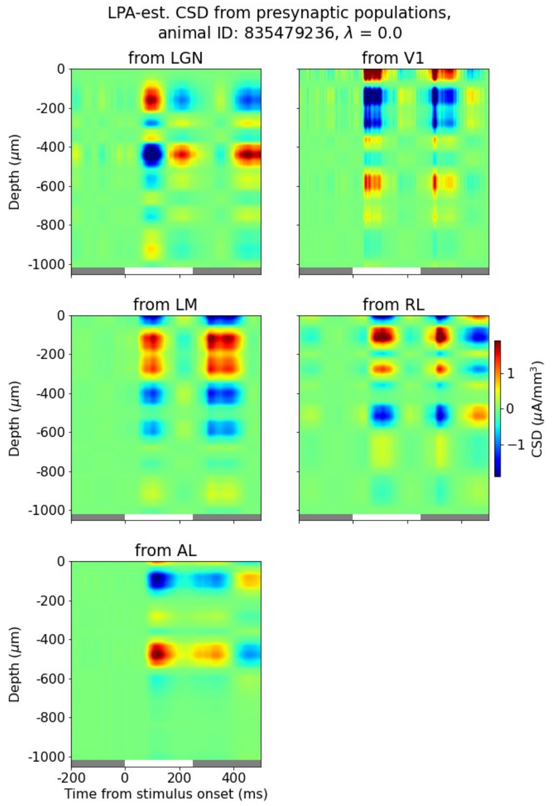

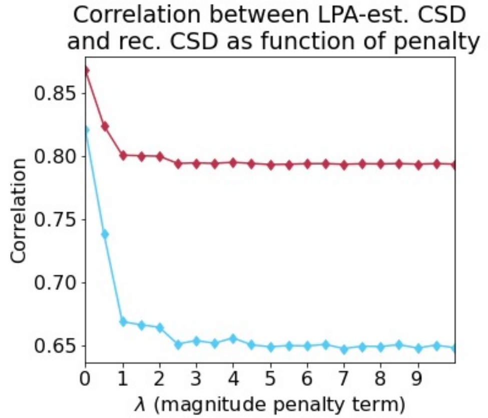

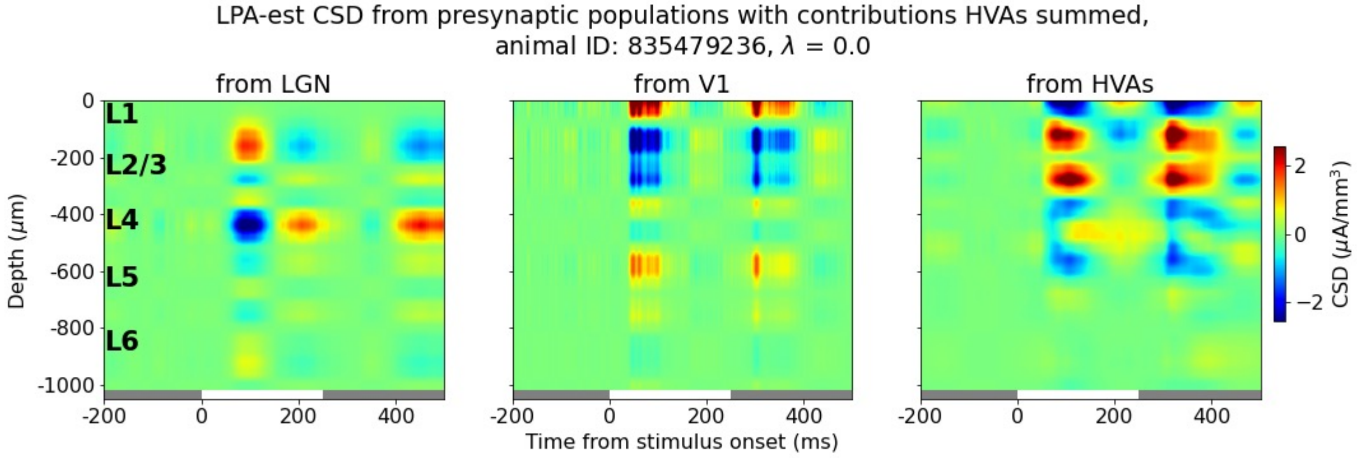

Session ID: 839068429

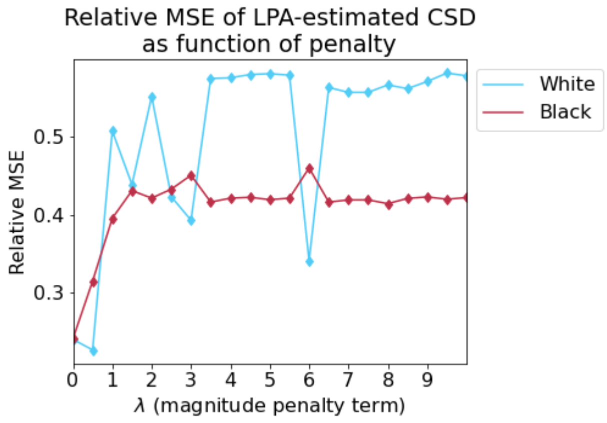

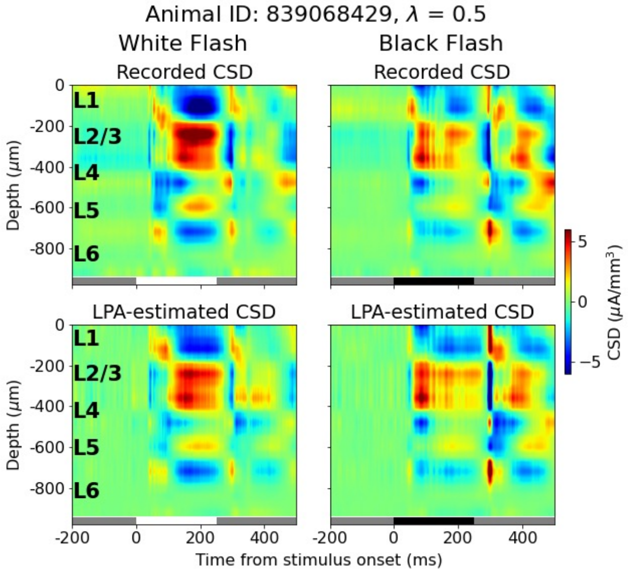

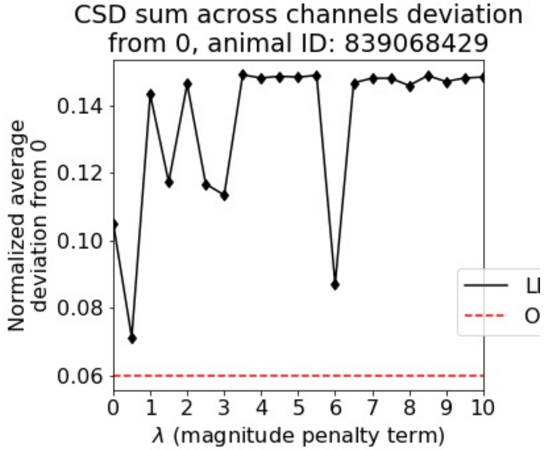

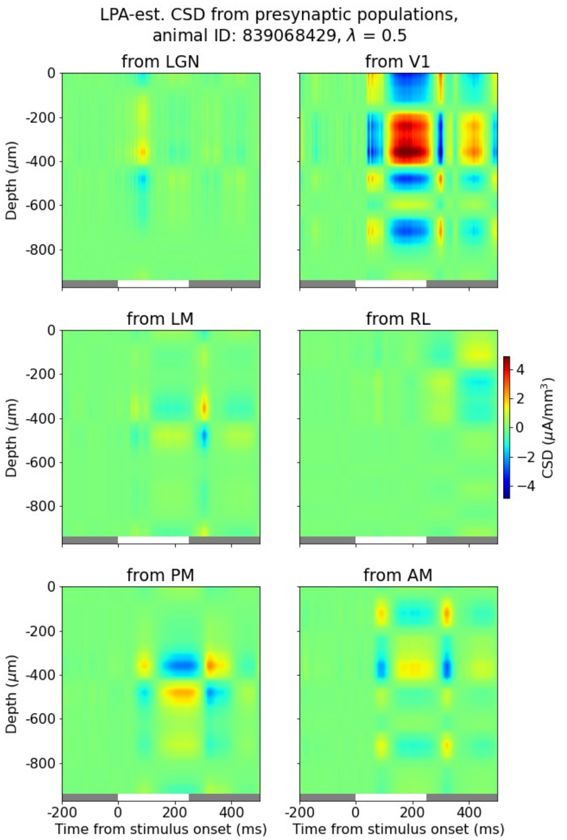

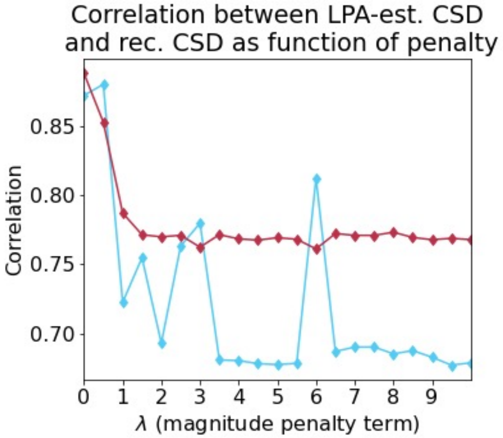

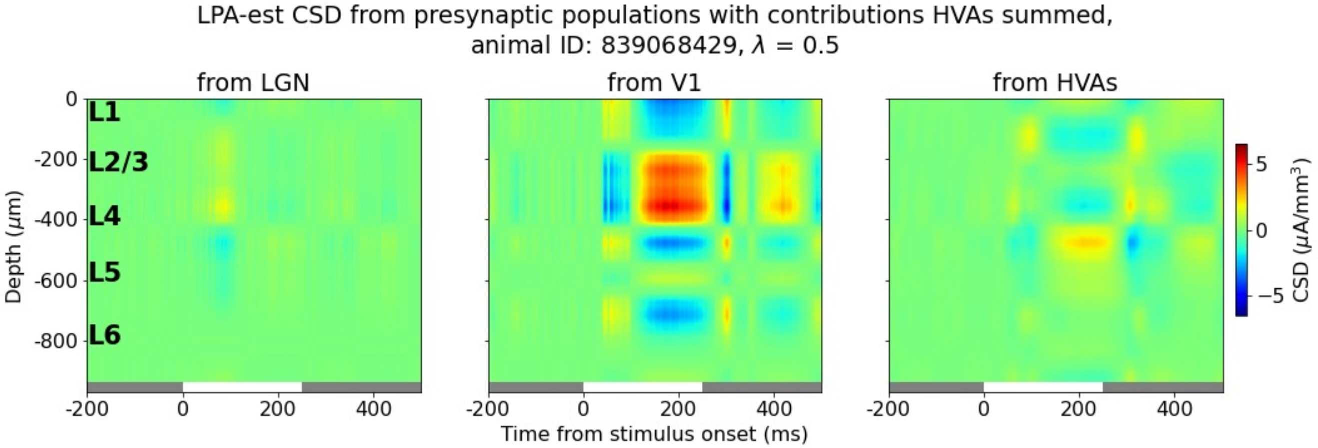

Supplement: S12 Fig — (PDF) [file pcbi.1011830.s012.pdf]
