# Supplementary material for: Uncovering population contributions to the extracellular potential in the mouse visual system using Laminar Population Analysis
Source: PLoS Comput Biol. 2024 Dec 12;20(12):e1011830. doi: 10.1371/journal.pcbi.1011830 (PMC11670981; doi:10.1371/journal.pcbi.1011830)
Supplement: Attachment — Submitted filename: Response_to_decision_letter_PLOS.docx [file pcbi.1011830.s013.docx]

Dr. Hermann Cuntz

Academic Editor and Group Leader at Ernst Strüngmann Institute (ESI)

Deutschordenstr. 46

60528 Frankfurt am Main, Germany

Dr. Thomas Serre

Section Editor and Professor at Brown University

185 Meeting Street

Providence, RI 02912, USA

*Dear editors,*

*Thank you for inviting us to submit a new version of our manuscript titled “Uncovering population contributions to the extracellular potential in the mouse visual system using Laminar Population Analysis”. In the following, we have listed key points in the reviewers’ critique (*in regular font*) and our responses to these points (in italics). Changes in the manuscript text are quoted in blue here and have also been marked in blue in the manuscript file.*

**Reviewer #1**: This is a very thorough manuscript, using advanced computational modeling and data analysis to investigate the biophysical properties of neural signals. The authors apply a method that they call “Laminar Population Analysis” (LPA) to disentangle and thereby better interpret the local field potential. For that, they use both simulated and recorded data from mouse primary visual cortex (V1). The work is clearly positioned in previous literature, the methods are rigorous and well described. The figures are clear, and the results are very carefully interpreted. The results are only partially successful, as stated by the authors: “The contributions from each laminar population could not be satisfactorily separated and identified with LPA.” This limited success is particularly the case for layer 4. I have two comments about this aspect of the work.

*We thank the reviewer for their assessment and want to express our appreciation for their insightful comments.*

1. First, neurons in V1 are thought to be driven preferentially by oriented edges, while the only stimuli used in this study are full-field flashed. The activation with full field flashes can be expected to be simpler, not leading to a differential effect between layer 4 and other layers, compared to when using patterned stimuli such as gratings, as also shown in studies in primates (see Schroeder et al. 1991 Vision Research, https://doi.org/10.1016/0042-6989(91)90040-c). It would therefore be relevant to at least compare the results to Gabor patches, which would be possible using the dataset from the study in mice that was used for the current work (Siegle et al. 2021 Nature).

*a.*  *We agree with the reviewer that patterned stimuli could potentially lead to greater differentiation in the response between different populations. However, with a single probe recording activity within a structure like V1, the LPA method is only suited for unpatterned stimuli. The reason for that is that for unpatterned stimuli, the spiking activity recorded from neurons near the probe can reasonably be assumed representative of the spiking activity of all neurons in the column. For patterned stimuli, this is unlikely to hold true because neurons close to the probe and those laterally further away are exposed to different stimulation patterns. In a multi-shank arrangement where spiking activity is recorded at multiple different locations in the column, patterned stimuli may also be appropriate for the application of the LPA method.*

2.	Second, layers in the mouse visual cortex are not so clearly differentiated as in primates, in particular lacking a clearly defined layer 4c (Lein et al. 2017; https://doi.org/10.1146/annurev-neuro-070815-013858; Jorstad, …, Lein 2023 https://doi.org/10.1126/science.adf6812). It would therefore be expected that LPA would work much better in primate V1. I can imagine that analyzing data from previous laminar studies from primate V1 would go beyond the aim of the current study. But it would be relevant to add that this difference between species could be a possible explanation for the lack of success in isolating layer 4 in mice.

*a.*   *This is a good point and would indeed constitute an interesting future direction for this work. We have added a discussion of the cross-species differences in laminar differentiation and its consequences for the application of LPA to identify layers on lines 642-650*:

The organization, cell type composition, thickness, and other properties of layers vary between different cortical structures and between species. For example, in primate V1, layer 4 comprises three different sublayers and makes up a larger proportion of V1 thickness than layer 4 in rodent V1 (Lein et al., 2017). The level of differentiation between layers, such as layer 4 vs. layer 5, etc., also varies across species, where layers in primate cortex are more distinct than in rodent cortex. One would therefore expect an even better layer identification, especially of layer 4, from primate data. Thus, an interesting future direction would be to test the application of LPA to ECP data recorded in other species, such as primates.

**Reviewer #2:** The correct interpretation of LFP in vivo has been the focus of many debates and studies. The authors here present a novel approach aiming at disentangling the contribution of different populations "based on physiological rather than statistical assumptions", building on their previous work and in particular on the Laminar Population Analysis published in 2007.

The aim of the work is clear and so are the description of the methods and the illustration of the results, including the figures. Also a "minor" element of the work as the idea of penalizing deviations from zero in the summed CSD has definitely a general interest.

*We are thankful for the reviewer’s comments, especially the observation that penalizing deviations from zero in the summed CSD has general interest.*

1. **A)** However, I think that there is a central point that might not have been properly assessed by the authors. One of the claims of the abstract is that the method is able to disentangle the contribution to LFP of local and remote inputs. But if I correctly understand, in order to do this the authors need to exploit the knowledge of the subcortical and remote cortical firing rates, which are available from the model selected as ground truth. Now, how can this be useful in experimental settings where these firing rates are typically not available?

***A)*** *The first part of the LPA algorithm, where laminar positions are identified from the MUA, can be applied even if there are no recorded data from structures that are external to the one investigated (e.g. V1). This application of the LPA method therefore has utility even in experiments with limited simultaneous recordings from different structures.*

*Furthermore, there are also conditions in which the effects of external inputs in LFP generation may be limited, such as when the animal is anesthetized (Lamme et al., 1998; Sellers et al., 2013; Keller et al., 2020). Indeed, in the original paper in which the LPA method was presented, it was applied to experimental data from rat barrel cortex where there was no MUA data for external structures. Those recordings were made while the rat was lightly anesthetized and the timing of the onset of evoked LFP responses and the MUA in the barrel cortex suggested that, in these experiments, most of the LFP was generated by recurrent activity within the barrel cortex.*

*However, the reviewer is correct that in experiments where data on firing rates/MUA from external structures are lacking and their contribution cannot be expected to be negligible, the utility of LPA in disentangling LFP contributions from local and remote inputs will be limited. It was for this reason that we set the criterion that an animal had to have ECP recordings in LGN and at least one higher visual area to be included in the demonstration of LPA on experimental data in our study.*

**B)** This also puts another claim at stake. The authors state that assumptions underlying ICA and PCA decomposition of LFP are not always valid, and I agree, but then claim that their method is superior. But could it be otherwise, given that the LPA approach is provided with data from recordings that are not included in the ICA and PCA decomposition? Should'nt the comparison be performed starting from the same set of recordings?

There is one sentence in the limitation section that might refer to this - but seems more focusing on the unlikelihood of having access to all the local excitatory firing rates, which is indeed a limitation but less severe than the unlikelihood of knowing excitatory firing rates also from all projecting regions.

Could the author please clarify this key issue?

***B)*** *We agree that the LPA method has an advantage over ICA and PCA in that data from external recordings can easily be included in the decomposition, but it is not given that LPA must necessarily perform better. For example, there may be neural data for which different sources of LFP generation are truly statistically independent. In these cases, ICA can disentangle LFP contributions as well as LPA, even if recordings from external structures are not utilized in the decomposition with ICA. Our claim is not that the LPA method has to produce a significantly better decomposition than ICA in all conditions and structures, only that it might have wider applicability (provided that necessary data from external structures are available). The mouse primary visual cortex, under these simulated and experimental conditions, is a structure for which the LPA method is better suited to decompose LFP than ICA.*

**Reviewer #3**: This study describes and evaluates Laminar Population Analysis, a decomposition algorithm which facilitates interpretation of extracellular signals, an important research area. The algorithm is evaluated on the Allen Brain V1 model and experimental data. The choice of model is very strong as it is highly detailed and has been thoroughly validated against experimental data (including recently LFP/CSD patterns in a recent publication). Similarly, the experimental datasets used are high quality and provide abundant data for statistical validation. The authors demonstrate the usefulness of the algorithm to 1) identify laminar positions and temporal profiles of population rates, and 2) estimate CSD sinks and sources generated by long-range inputs and V1 recurrent activity, outperforming previous PCA and ICA methods. Although the algorithm's performance separating the contribution of different V1 layers is limited, I believe this is an important negative result to report. It can lead to a better understanding of the system -- e.g. high synchrony is limiting this separation, as pointed out by the authors -- and sets a benchmark for future improved versions or other algorithms. The authors also demonstrate how to apply the algorithm to real V1 in vivo data, making this study substantially more impactful.

*We are grateful for the reviewer’s perspectives and encouraging remarks.*

Below I list some concerns and suggestions to address before publication of the manuscript:

1.	Abstract: It would be useful to mention in the abstract the negative results regarding estimation of generators in cortical layers, and maybe the high synchrony cause -- I understand there is limited space, but one sentence or less would probably be enough.

*We agree and have included the following sentence in the abstract: The contributions from different cortical layers within V1 could, however, not be robustly separated and identified with LPA. This is likely due to substantial synchrony in population firing rates across layers, which may be reduced with other stimulus protocols in the future.*

2.	Line 32: a brief explanation of return currents might be useful for those not familiar with the topic

a.	*The following explanation (in blue) has been added to the sentence on lines 29-37 in the manuscript:
Despite being easier to evaluate than the LFP, however, the interpretation of current sinks and sources is also not straightforward. Current conservation dictates that any inward current must be balanced by an outward current. Thus, a synaptic input current is balanced by a stream of ions leaving the neurons (Halnes et al., 2024), and the balancing outward current during synaptic input is referred to as the return current. The return current during an excitatory synaptic input would be made up of positive ions leaving the neuron and show up as a current source in a CSD plot. However, an inhibitory synaptic input current will also produce a current source in a CSD plot, and there is typically no easy way to disambiguate the two potential origins.*

3.	Line 173: "the correlations between the LPA-estimated and the simulated MUA" - I think this first results section could benefit from some more details of how the algorithm works and what it is trying to do. For example, here it might be confusing to readers why the algorithm is generating an estimate of the MUA if the MUA in itself is an input to the model; maybe clarify that it is trying to decompose the MUA into its components/populations.

a.	*The following sentences have been added on lines 174-182:
The purpose of applying LPA to the MUA is to identify the positions of populations in a structure and to estimate the temporal profile of their population firing rates. In the optimization procedure, the total MUA calculated from the estimated population positions and temporal profiles is compared to the total, original MUA to which LPA is applied. The procedure is stopped when the difference between the original MUA and the MUA calculated from the LPA-estimated positions and temporal profiles is minimized. We here report the correlation and relative mean square error between the original, simulated MUA and the MUA calculated from the final, optimized LPA estimate.*

4.	Line 184: "whether the layer position of each recording electrode determined by LPA corresponds to the true layer position of that electrode" - similarly, here it is unclear how the algorithm is how the electrode location is determined by LPA; there is a lot of detail about the Metrics (which could be partly moved to Methods), but it is missing more intuitive insights into what LPA is doing / encoding

*a.*   *The LPA algorithm predicts the location of the layers, but the predicted location can only be assessed at the position of the electrode contacts – that is where we have data points. We have added this explanation on lines 196-198 in the manuscript.*

5.	Line 251: would be useful to clarify if 1) the population temporal profiles include both excitatory and inhibitory neurons, and if so, 2) if they are multiplied by the same kernel given that E vs I would have opposite effects on the CSD ?

*This is mentioned in the limitations section of the Discussion. The population profiles likely include spikes from both inhibitory and excitatory neurons, but the spikes from excitatory neurons can be expected to dominate the estimate as the MUA from excitatory populations make up 88.5 % of the total MUA variance (see Fig. S1). However, this point could have been made sooner to enhance clarity and readability. Therefore, we added this point on lines 261-273 in the manuscript:*

*As noted above, the temporal profiles estimated from MUA will likely predominantly reflect the firing of excitatory neurons. However, unless excitatory and inhibitory cells happen to be spatially separated, which they are not here, their contributions to the MUA cannot be separated and there will likely be minor contributions from inhibitory spikes in the temporal profiles estimated for each population. Spikes from excitatory and inhibitory neurons will generate currents with opposite signs in the postsynaptic cells; excitatory synaptic input will generate a current sink while inhibitory synaptic input will generate a current source. These opposite effects will not be captured when a single temporal profile is used for a whole laminar population that contains both inhibitory and excitatory neurons. However, since the MUA generated from excitatory spikes make up the vast majority of the total MUA (88.5 %; see Supplementary Fig. S1), the error from this simplification can be expected to be minor.*

6.	Fig 3 - some font sizes too small

a.	*The font sizes have been increased in the revised manuscript.*

7.	Line 348: "did not improve" - this was surprising to me. Any intuition as to why firing rates provide a worse performance that MUA? intuitively it seems it should be the other way around?

 Line 371: "We continued to use the firing rates" - at this point, this choice was not clear since before you had shown worse performance.

 Line 402: "increased from 0.46 to 0.6" - this is using the firing rates instead of the MUA; but comparing MUA-based results both the LGN and FB decreased significantly. I guess this explains the choice of firing rates method? This should all be clarified to avoid confusing the reader.

 Line 465: It was great to see how the approach was iteratively improved. I was still surprised to see that using MUA the LGN result was very low (0.03) -- any explanation for this? Again these results justify the use of firing rates and not MUA. However, when applying to experimental data, one would need to use MUA? Should the MUA results be therefore reported instead of the firing rate ones? And since the MUA results are worse, does this reduce the applicability of the algorithm?

*Our reason for using model firing rates rather than firing rates estimated from MUA with LPA for V1 was not based on the results. That is, the choice was not made because the results were necessarily better with model firing rates. Rather, it was done because it is not clear whether the firing rates calculated from the spikes in the external input to the model can be directly combined with the firing rates estimated from MUA in V1.*

*We do not simulate the ECP for the structures providing external input to the model – the LGN, LM, and background. Therefore, the temporal profile of the firing rates in these structures cannot be estimated from MUA in the model; the firing rates used in the LPA decomposition are simply calculated from the spikes in the input from these structures. The magnitudes of the temporal profiles of firing rates estimated from MUA in V1 are not directly comparable to the magnitudes of the firing rates calculated from spikes in the external structures. Thus, if the firing rates calculated from spikes in external structures are used together with temporal profiles of firing rates estimated from MUA in V1, this may result in a misestimation of the LFP contribution from V1 relative to that generated by the external input. This might also be the explanation for the relatively low correlation for LGN when using firing rates estimated from MUA in the decomposition of model data. As the temporal profiles of the firing rates can be estimated from the MUA for all structures (for which we have data) in the experiments, this issue of lack of compatibility in firing rate estimates does not affect the application of LPA to experimental data.*

*This justification was given on lines 330-340 in the original manuscript (from line 355 in the revised manuscript), but we acknowledge that more emphasis could have been put on it and that the point could have been made clearer. We have therefore expanded on the original justification on lines 358-374 in the revised manuscript with the details provided above.*

8.	Line 568: This results subsection would benefit from a few final sentences clarifying the meaning of the results and any issues, as was done in previous subsections.

*The following summarizing sentences have been added on lines 602-614:*

*The prediction of a sink generated by feedback in the upper layers, L1 and L2/3, with a source below it in L4 and parts of L5, aligns with the predicted CSD contribution from feedback in the model. The magnitude of the estimated major sink in L5 generated by feedback in the experiment deviates from the magnitude predicted by simulations, but the experimental prediction may reflect synaptic innervation from HVAs in this layer, which is suggested by anatomical data (Shen et al., 2022).* *In summary, while there are important discrepancies, the LPA-estimated positions of prominent sinks and sources generated by different populations largely align with their expected distributions based on anatomical data and the predictions made with the model. More detailed data are required to determine the validity of the LPA estimates for experimental data, but the preliminary comparison to model predictions and anatomical data is at least an indication that the estimated population contributions are reasonable.*

9.	Line 626: It would be interesting to demonstrate that high synchrony is causing the issue, perhaps using a V1 simulation with different inputs, even if not realistic. However, this is just a suggestion and not strictly required for this revision, perhaps to be done in future work.

*This would indeed be an interesting avenue to explore. However, we leave it to future studies of the LPA method.*

**References**

Halnes G, Ness TV, N’ss S, Næss S, Hagen E, Pettersen KH, et al. Electric Brain

Signals: Foundations and Applications of Biophysical Modeling, Chapter 8: Local Field Potentials. Cambridge University Press; 2024.

Keller AJ, Roth MM, Scanziani M. Feedback generates a second receptive field in

neurons of the visual cortex. Nature. 2020;582(7813):545–549.

Lein ES, Belgard TG, Hawrylycz M, Molnár Z. Transcriptomic perspectives on

neocortical structure, development, evolution, and disease. Annual Review of

Neuroscience. 2017;40(1):629–652

Lamme VA, Zipser K, Spekreijse H. Figure-ground activity in primary visual

cortex is suppressed by anesthesia. Proceedings of the National Academy of

Sciences. 1998;95(6):3263–3268.

Sellers KK, Bennett DV, Hutt A, Fröhlich F. Anesthesia differentially modulates

spontaneous network dynamics by cortical area and layer. Journal of

neurophysiology. 2013;110(12):2739–2751.

Shen S, Jiang X, Scala F, Fu J, Fahey P, Kobak D, et al. Distinct organization of

two cortico-cortical feedback pathways. Nature Communications. 2022;13(1):6389.
