# Supplementary material for: Uncovering population contributions to the extracellular potential in the mouse visual system using Laminar Population Analysis
Source: PLoS Comput Biol. 2024 Dec 12;20(12):e1011830. doi: 10.1371/journal.pcbi.1011830 (PMC11670981; doi:10.1371/journal.pcbi.1011830)
Supplement: Attachment — Submitted filename: Response_to_decision_letter_PLOS_round_2.docx [file pcbi.1011830.s014.docx]

Dr. Hermann Cuntz

Academic Editor and Group Leader at Ernst Strüngmann Institute (ESI)

Deutschordenstr. 46

60528 Frankfurt am Main, Germany

Dr. Thomas Serre

Section Editor and Professor at Brown University

185 Meeting Street

Providence, RI 02912, USA

*Dear editors,*

*Thank you for inviting us to submit a new version of our manuscript titled “Uncovering population contributions to the extracellular potential in the mouse visual system using Laminar Population Analysis”. In the following, we have listed key points in the reviewers’ critique (*in regular font*) and our responses to these points (in italics). Changes in the manuscript text are quoted in blue here and have also been marked in blue in the manuscript file.*

**Reviewer #1**: I had two comments. The second was well answered by the authors, but the first was not. In response to my first comment, the authors wrote :
"We agree with the reviewer that patterned stimuli could potentially leadto greater differentiation in the response between different populations. However, with a single probe recording activity within a structure like V1, the LPA method is only suited for unpatterned stimuli. The reason for that is that for unpatterned stimuli, the spiking activity recorded from neurons near the probe can reasonably be assumed representative of the spiking activity of all neurons in the column. For patterned stimuli, this is unlikely to hold true because neurons close to the probe and those laterally further away are exposed to different stimulation patterns. In a multi-shank arrangement where spiking activity is recorded at multiple different locations in the column, patterned stimuli may also be appropriate for the application of the LPA method." This point might be true for complicated natural stimuli. However, I suggested to use gratings, which are one dimensional stimuli and would therefore give very similar input to neighboring columns. One-dimensional gratings is what has been most commonly used to measure CSD responses in V1 (together with checkerboard stimuli, so 2-dimensional gratings). Finally, measuring the responses using either drifting gratings or Gabor patches would be possible using the dataset that was used for the current work (Siegle et al. 2021 Nature).

*We thank the reviewer for the suggestion. However, we do not see how gratings can be used with the present version of LPA and the data from the Allen Institute (Siegle et al, 2021, Nature). We concur that this limitation could have been clearly outlined in the manuscript and added the following paragraph on lines 886-896 (in the revised manuscript) to clarify this point: Lastly, since the spiking of all neurons in the column can in principle contribute to the measured LFP through their afferent connections, LPA requires an estimate for the spiking for all neurons in the column. The present application of the LPA method was thus limited to full-field flashes, that is, a spatially unpatterned stimuli. In this situation the spiking activity recorded from neurons near the probe in a particular layer can reasonably be assumed to be representative of the spiking activity of all neurons in this layer in the column. With spatially patterned stimuli, even as simple as a grating, this is unlikely to hold true because neurons close to the probe and those laterally further way will in general have different firing activities. In a multi-shank arrangement where spiking activity is recorded at multiple different locations in the column, patterned stimuli may, however, also be appropriate for the application of the LPA method.*

Reviewer #2: The answers provided to my comments by the authors are correct and complete. Still, no improvement was made in the Discussion or at least in the Methods to make these two crucial points clearer to the reader. I definitely recommend to modify the manuscript to include the clarifications proposed by authors in the reply.

*We thank the reviewer for the recommendation and have now added the clarifications to the manuscript.*

*To make the first point raised by the reviewer clearer to the reader, we added the next two paragraphs on lines 860-885 (in the revised manuscript):*

*The inclusion of input from external structures in the application of LPA of course necessitates that the activity in those structures has been recorded simultaneously with the structure that LPA is applied to. In the application to simulated data, we had full knowledge of the activity in all structures providing input to V1. In the application to experimental data, however, there may be structures that provide a non-negligible input that are not included in the analysis due to a lack of recordings in those areas. This will be a source of error as the contributions from those structures will falsely be attributed to the input from the structures for which we happened to have recordings. In some experimental conditions, such as when the animal is anesthetized, the effects of external inputs in LFP generation may be more limited (Lamme et al., 1998, Sellers et al., 2013, Keller et al., 2020).*

*In those conditions, the LPA method can have utility even if there are no recordings from external structures. Indeed, in the original presentation of the LPA method, it was applied to experimental data from rat barrel cortex where there was no MUA data for other areas (Einevoll et al., 2007).. Those recordings were made while the rat was lightly anesthetized and the timing of the onset of evoked LFP responses and the MUA in the barrel cortex suggested that, in these experiments, most of the LFP was generated by recurrent activity within the barrel cortex. Additionally, the first part of the LPA algorithm, where laminar positions are identified from the MUA, can be applied even if when there are no recorded data in external structures and there are no experimental conditions that limit their influence on the structure in question. The identification of laminar positions from MUA data only depends on differing MUA magnitudes across layers, and this application of the LPA method therefore has utility even in experiments with limited simultaneous recordings from different structures. If there are no conditions that can be expected to limit the effects of input from external structures, however, then a lack of simultaneous recordings from those structures would constitute a limitation in which experimental LFP data LPA can be applied to.*

*To address the second point raised by the reviewer, we have added the following paragraph on lines 763-770 (in the revised manuscript): It should be noted that even though the LPA method has an advantage over ICA and PCA in that data from external recordings can easily be included in the decomposition, it is not given that LPA must necessarily perform better in all conditions and structures. For example, there may be neural data for which different sources of LFP generation are truly statistically independent. In these cases, ICA can disentangle LFP contributions as well as LPA, even if recordings from external structures are not utilized in the decomposition with ICA.*

Reviewer #3: All comments have been successfully addressed. Congratulations on this important work.

**References**

Einevoll G, Pettersen K, Devor A, Ulbert I, Halgren E, Dale A. Laminar

Population Analysis: Estimating Firing Rates and Evoked Synaptic Activity

From Multielectrode Recordings in Rat Barrel Cortex. Journal of

neurophysiology. 2007;97:2174–90. doi:10.1152/jn.00845.2006

Halnes G, Ness TV, N’ss S, Næss S, Hagen E, Pettersen KH, et al. Electric Brain

Signals: Foundations and Applications of Biophysical Modeling, Chapter 8: Local Field Potentials. Cambridge University Press; 2024.

Keller AJ, Roth MM, Scanziani M. Feedback generates a second receptive field in

neurons of the visual cortex. Nature. 2020;582(7813):545–549.

Lein ES, Belgard TG, Hawrylycz M, Molnár Z. Transcriptomic perspectives on

neocortical structure, development, evolution, and disease. Annual Review of

Neuroscience. 2017;40(1):629–652

Lamme VA, Zipser K, Spekreijse H. Figure-ground activity in primary visual

cortex is suppressed by anesthesia. Proceedings of the National Academy of

Sciences. 1998;95(6):3263–3268.

Sellers KK, Bennett DV, Hutt A, Fröhlich F. Anesthesia differentially modulates

spontaneous network dynamics by cortical area and layer. Journal of

neurophysiology. 2013;110(12):2739–2751.

Shen S, Jiang X, Scala F, Fu J, Fahey P, Kobak D, et al. Distinct organization of

two cortico-cortical feedback pathways. Nature Communications. 2022;13(1):6389.
